# Genome-wide characterisation and analysis of bHLH transcription factors related to tanshinone biosynthesis in *Salvia miltiorrhiza*

**DOI:** 10.1038/srep11244

**Published:** 2015-07-15

**Authors:** Xin Zhang, Hongmei Luo, Zhichao Xu, Yingjie Zhu, Aijia Ji, Jingyuan Song, Shilin Chen

**Affiliations:** 1 Institute of Medicinal Plant Development, Chinese Academy of Medical Sciences & Peking Union Medical College, Beijing 100193, China; 2 Institute of Chinese Materia Medica, China Academy of Chinese Medical Sciences, Beijing 100700, China; 3 Chongqing Institute of Medicinal Plant Cultivation, Chongqing 408435, China

## Abstract

*Salvia miltiorrhiza* Bunge (Labiatae) is an emerging model plant for traditional medicine, and tanshinones are among the pharmacologically active constituents of this plant. Although extensive chemical and pharmaceutical studies of these compounds have been performed, studies on the basic helix-loop-helix (bHLH) transcription factors that regulate tanshinone biosynthesis are limited. In our study, 127 *bHLH* transcription factor genes were identified in the genome of *S. miltiorrhiza,* and phylogenetic analysis indicated that these *SmbHLHs* could be classified into 25 subfamilies. A total of 19 sequencing libraries were constructed for expression pattern analyses using RNA-Seq. Based on gene-specific expression patterns and up-regulated expression patterns in response to MeJA treatment, 7 *bHLH* genes were revealed as potentially involved in the regulation of tanshinone biosynthesis. Among them, the gene expression of *SmbHLH37*, *SmbHLH74* and *SmbHLH92* perfectly matches the accumulation pattern of tanshinone biosynthesis in *S. miltiorrhiza*. Our results provide a foundation for understanding the molecular basis and regulatory mechanisms of bHLH transcription factors in *S. miltiorrhiza*.

The basic helix-loop-helix (bHLH) transcription factor family is one of the largest transcription factor families in both animals and plants. More than 630 *bHLH* transcription factors have been identified in several important food crops^1^, and based on genome-wide analyses, 167, 177, 190 and at least 191 *bHLH* transcription factors have been predicted in the *Arabidopsis thaliana* L., *Oryza sativa* L., *Nicotiana tabacum* L. and *Vitis vinifera* L. genomes, respectively^1^,^2^,^3^. Interestingly, more *bHLH* transcription factors have been identified in the *A. thaliana* genome than in most animal species^4^. The completion of the *Salvia miltiorrhiza* Bunge genome sequence has facilitated studies on *bHLH* transcription factors in *S. miltiorrhiza*, a plant belonging to the Labiatae family that is an emerging model plant for traditional medicine^5^. For thousands of years, *S. miltiorrhiza* has been widely used for the treatment of cardiovascular disease, amenorrhea and dysmenorrhea^6^. Tanshinones, a major group of pharmacologically active constituents of this plant, mainly accumulate in the root periderm, and their biosynthesis is regulated by MeJA^7^,^8^,^9^. Although numerous chemical and pharmaceutical studies of *S. miltiorrhiza* have been conducted, investigations of transcription factors are limited, especially with regard to the regulation of transcription factors involved in tanshinone biosynthesis. Sequencing data show that are 15 members of the *SPL* transcription factor family in the *S. miltiorrhiza* genome^10^, and genome-wide characterisation of the *R2R3-MYB* transcription factor subfamily has also been performed^11^. Furthermore, the repressor role of SmMYB39 in the rosmarinic acid pathway has been characterised through overexpression and RNAi-mediated silencing^12^. An analysis of the *S. miltiorrhiza* transcriptome revealed 1,341 unigenes belonging to 46 transcription factor families, including 76 unigenes of *bHLH* transcription factors^13^. Additionally, upon treatment with yeast extract and Ag^+^^14^, 412 transcription factors (40 *bHLH* genes) were identified among the differentially expressed genes in *S. miltiorrhiza* hairy roots.

Nonetheless, transcriptome sequencing is not likely to identify all *bHLH* genes simultaneously, especially those genes expressed under specific conditions. Thus, to systematically elucidate the regulatory role of bHLH transcription factors in *S. miltiorrhiza*, a genome-wide analysis of *bHLH* transcription factors was performed. Based on various expression patterns and the results of MeJA treatment and qPCR analyses, 3 *bHLH* genes that likely regulate tanshinone biosynthesis were identified. The findings from this study provide a foundation for understanding the molecular basis and regulatory mechanisms of bHLH transcription factors in *S. miltiorrhiza*.

## Results

### Gene prediction, sequence features and phylogenetic analysis

A total of 127 *SmbHLHs* containing ORF sequences were identified and named *SmbHLH1-SmbHLH127* (Table S1). The number of *bHLH* genes in *S. miltiorrhiza* is similar to the number of *bHLH* genes in *A. thaliana*, *O. sativa*, *N. tabacum* and *V. vinifera*. The gene length of *SmbHLHs* was found to vary from 459 bp (*SmbHLH58*) to 8,663 bp (*SmbHLH61*) (Table S2), and the length of *SmbHLH* cDNAs varied from 255 bp (*SmbHLH71*) to 2,154 bp (*SmbHLH81*) (Table S2). The molecular weights of the predicted proteins range from 9,533.9 Da (SmbHLH71) to 79,156.6 Da (SmbHLH81) (Table S2), and the theoretical isoelectric points are predicted to range from 4.8 (SmbHLH69) to 9.9 (SmbHLH126 and SmbHLH127) (Table S2).

**Figure 1:**
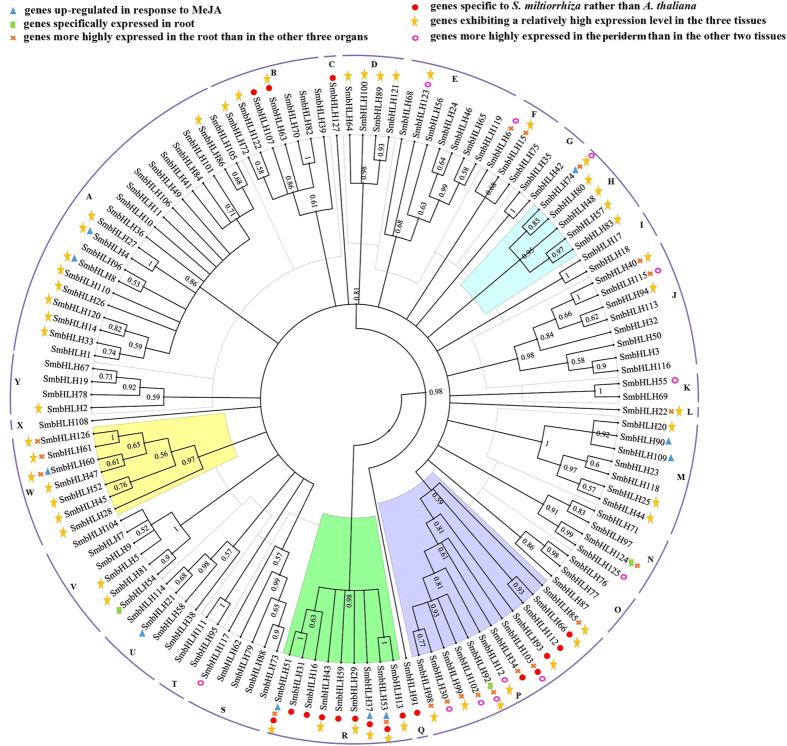
An un-rooted phylogenetic tree of the *bHLH gene family in S. miltiorrhiza.* The amino acid sequences were aligned using Clustal W, and the phylogenetic tree was constructed using neighbour-joining criteria. The letters (A-Y) represent the main subfamilies. The putative *bHLH* genes involved in the regulation of tanshinone biosynthesis are *SmbHLH37* (subfamily R), *SmbHLH51* (subfamily R), *SmbHLH53* (subfamily R), *SmbHLH60* (subfamily W), *SmbHLH74* (subfamily H), *SmbHLH92* (subfamily P), and *SmbHLH103* (subfamily P).

A neighbour-joining tree constructed among the 127 *bHLH* members identified in *S. miltiorrhiza* indicated a total of 25 distinct subfamilies (designated A to Y) (Fig. 1), with subfamily A having the largest number of members (20 *SmbHLHs*) and subfamilies C, L, Q and X the fewest (1 *bHLH*). An un-rooted phylogenetic tree was constructed using the *bHLH* gene family in *S. miltiorrhiza* and 5 other *bHLH* genes (*AtTT8*, *NtMYC2a*, *NtMYC2b*, *CjbHLH1*, and *CrMYC2*) that regulate secondary metabolism^15^,^16^,^17^,^18^. Interestingly, *AtTT8*, *NtMYC2a*, *NtMYC2b*, and *CrMYC2* cluster in subfamily R, and *CjbHLH1* clusters in subfamily O. According to the alignment, the transcription factors in subfamily R (9 genes) are putative regulators of tanshinone biosynthesis.

### Structure analysis

**Figure 2:**
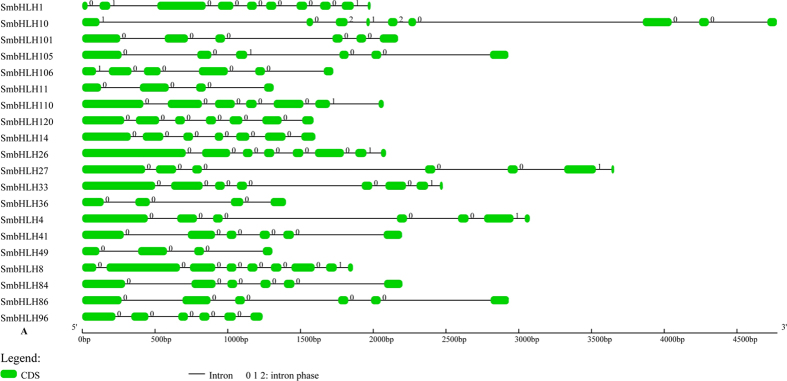
The structural features of each *bHLH* gene in subfamily A. The exons are represented by green round-cornered rectangles. The black lines connecting two exons represent introns. The numbers above the line represent the intron phase.

Structure analyses of all of the *bHLH* genes revealed that the number of exons varies from 1 to 12; a total of 6 genes are intronless. The genes in the 25 subfamilies have an average exon number per gene ranging from 1 (subfamily U) to 9 (subfamily D), and the average exon number in subfamilies G, H and L is 5. The same pattern was observed in subfamilies C, K and M but with an average number of 4. The 6 intronless genes are distributed across 3 subfamilies, particularly subfamily U, in which all 4 genes are intronless. The remaining 2 intronless genes belong to subfamily R and subfamily T. Although these subfamilies contain only 1 gene, all of the other genes in subfamilies K and N contain 4 and 2 exons, respectively. The structural features of each *bHLH* gene in subfamily A are listed in Fig. 2, and those of the others are listed in Figure S1.

Exons with the same splicing phase at both ends are called symmetric exons, and an excess of symmetric exons and phase 0 introns is likely to facilitate exon shuffling, recombinational fusion and protein domain exchange^19^,^20^. According to the 575 exons analysed herein, 230 exons are symmetric with phase 0 introns, 2 exons are symmetric with phase 1 introns, and 11 exons are symmetric with phase 2 introns. Among the 448 introns of the *bHLH* genes, 363 are phase 0, 41 are phase 1 and 44 are phase 2. Therefore, our analysis of the *bHLH* gene structures strongly indicates a great *bHLH* transcription factor family diversity in *S. miltiorrhiza*.

### Conserved motif analysis

A total of 22 conserved motifs were characterised (motifs 1–22) (Table S3). The SmbHLHs in subfamily A contain the highest number of motifs (11 types), whereas the SmbHLHs in subfamilies C, T, U and X contain the smallest number of motifs (2 types). Additionally, the average motif number per sequence varies across subfamilies, ranging from 2 (subfamilies C, N, T and U) to 7 (subfamily V).

**Figure 3:**
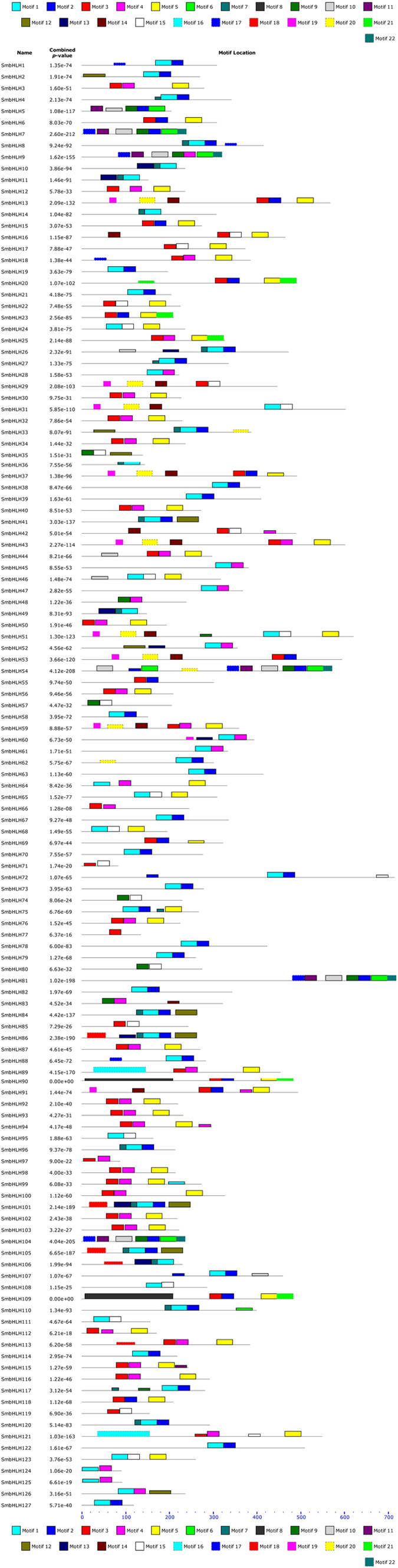
The distribution of conserved motifs in each *bHLH* gene. The relative positions of each conserved motif within the bHLH protein are shown in colour.

In most cases, a motif is repeated only once, but a few special cases were found. Motif 2 is repeated twice in SmbHLH72 and SmbHLH107. Motif 4 and motif 21 are repeated twice in SmbHLH94 and SmbHLH20, respectively. The highest number of motif repetitions occurs in SmbHLH54, in which motifs 2, 6 and 10 are each repeated twice. Furthermore, certain conserved motifs are nested in specific subfamilies in the un-rooted phylogenetic tree (Fig. 1). For example, motif 16 is shared by 2 members (SmbHLH89 and SmbHLH121) in subfamily D; motif 8 is shared by 2 members (SmbHLH90 and SmbHLH109) in subfamily M; and motif 21 is shared by 5 members (SmbHLH20, SmbHLH23, SmbHLH25, SmbHLH90, and SmbHLH109) in subfamily M. Motifs 12, 13 and 18 are only distributed in subfamily A. Similarly, motifs 6, 10, 11, 17 and 22 are specific to subfamily V. The distribution of conserved motifs of each *bHLH* gene is listed in Fig. 3.

### Differential expression of *bHLH* genes in various organs

After mapping these clean reads to the *S. miltiorrhiza* genome database, the expression levels were estimated according to RPKM (reads per kilobase per million) values (Table S4). Among the 127 *bHLH* genes, the expression of 117 *bHLH* genes, comprising 24 subfamilies, was detected in at least one of the four organs tested. The other 10 genes, distributed across 8 subfamilies, were undetected (RPKM values of 0). Among the 117 detected genes, 99 were detected with expression in the flower, 93 in the leaf, 93 in the root and 99 in the stem.

**Figure 4:**
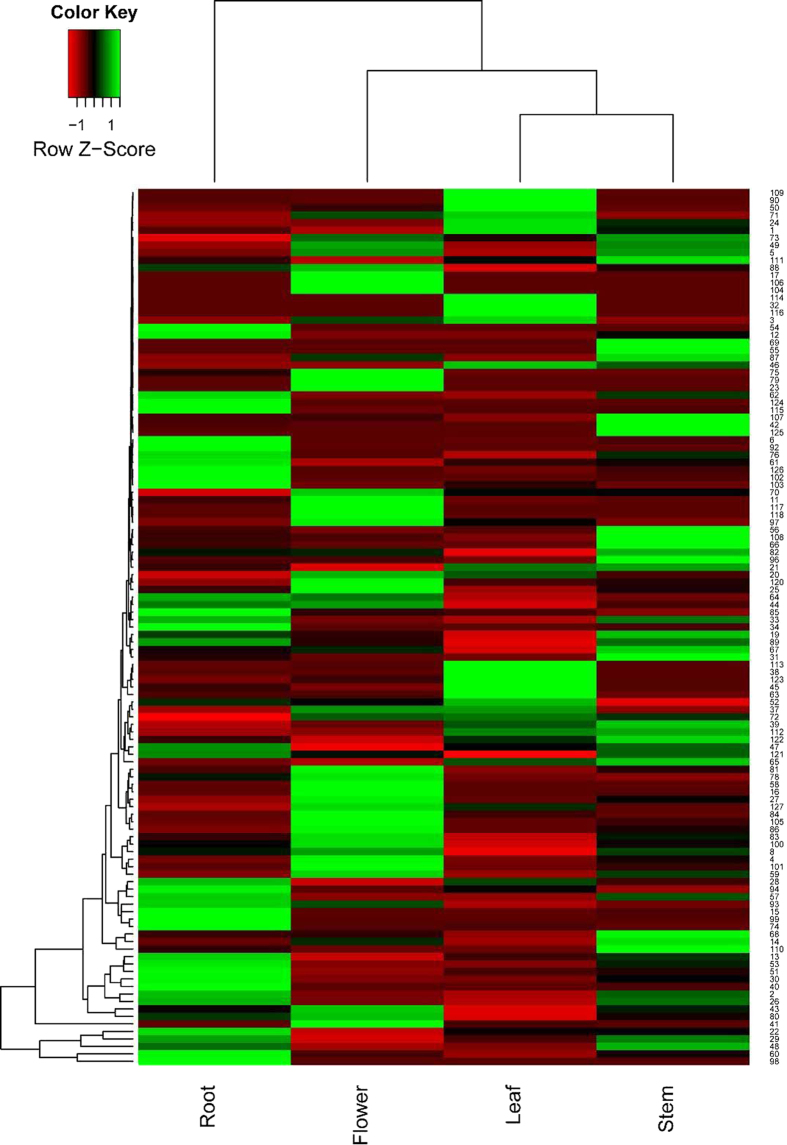
Heatmaps representing the expression profiles of *S. miltiorrhiza bHLH* genes in the flower, leaf, root and stem. The numbers to the right of the figure indicate the *bHLH* gene name. The colour scale is shown at the top. Higher expression levels are shown in green. The genes with RPKM values of 0 in the four tested organs are not included in the figure.

To understand the spatial transcriptional patterns of *bHLH* genes among various organs, hierarchy clustering was performed using the 117 genes detected. As illustrated in Fig. 4, 45 genes showed relatively high expression (RPKM values higher than 1) among the four organs. In addition, most of the *bHLH* genes exhibited diverse expression profiles. For example, 8 genes (*SmbHLH17*, *SmbHLH23*, *SmbHLH58*, *SmbHLH79*, *SmbHLH104*, *SmbHLH106*, *SmbHLH117* and *SmbHLH118*) were specifically expressed in the flower; 2 genes (*SmbHLH32* and *SmbHLH114*) were specifically expressed in the leaf; 3 genes (*SmbHLH54*, *SmbHLH92* and *SmbHLH124*) were specifically expressed in the root; and 3 genes (*SmbHLH55*, *SmbHLH69* and *SmbHLH125*) were specifically expressed in the stem (Fig. 1). Additionally, 17 genes exhibited higher expression levels in flowers than in vegetative organs (at least 2-fold higher than other organs, with RPKM values greater than 1). Furthermore, 22 genes exhibited higher expression levels in the root than in the other three organs, and this group included 2 genes (*SmbHLH92* and *SmbHLH124*) that were specifically expressed in the root (Fig. 1); however, the RPKM value of the other gene (*SmbHLH54*) specifically expressed in the root was 0.075. Two of the 22 genes (*SmbHLH51* and *SmbHLH53*) belong to subfamily R; 7 of the 22 genes belong to subfamily P; and 3 of the 22 genes belong to subfamily W.

### Differential expression of *bHLH* genes in various tissues

The expression of 101 *bHLH* genes, comprising 23 subfamilies, was detected in at least one of the three root tissues (periderm, phloem and xylem) tested (Table S5). A total of 26 genes, distributed among 12 subfamilies, were undetected (RPKM values of 0). Compared to the differential expression data for the four plant organs, 20 of these 26 genes were not detected in the root; 4 of the remaining 6 genes were detected in the flower, stem and leaf, with 1 of the 6 genes detected in the flower and stem, whereas 1 of the 6 genes was specifically expressed in the root. Of the 101 detected genes, the expression of 90 was detected in the periderm; 94 genes were found to be expressed in the phloem, with 97 expressed in the xylem. Eight of the 10 genes had an RPKM value of 0 in all four tested organs, and the RPKM values were 0 in the three root tissues for all 8 genes, except *SmbHLH10* and *SmbHLH77*. The RPKM values of *SmbHLH10* and *SmbHLH77* in the three tissues were less than 1 under normal conditions. A total of 52 *bHLH* genes with relatively high expression (RPKM values higher than 1) were detected across the three tissues (Fig. 1). Compared with the RPKM values of the four organs, 48 of the 52 genes (all, except *SmbHLH5*, *SmbHLH12*, *SmbHLH66* and *SmbHLH123*) exhibited relatively high expression levels in the root.

The highest percentage of lipid-soluble tanshinones is found in the periderm of the root. In the current study, 12 *bHLH* genes exhibited higher expression levels in the periderm (at least 2-fold higher than in other tissues, with RPKM values greater than 1) than in the two other tissues tested (Fig. 1). These genes were *SmbHLH6*, *SmbHLH12*, *SmbHLH30*, *SmbHLH55*, *SmbHLH74*, *SmbHLH92*, *SmbHLH102*, *SmbHLH103*, *SmbHLH115*, *SmbHLH117*, *SmbHLH123* and *SmbHLH125*. Seven of the 12 genes (all, except *SmbHLH12*, *SmbHLH55*, *SmbHLH117*, *SmbHLH123* and *SmbHLH125*) showed relatively high expression levels in the root.

### Up-regulated *bHLH* genes in response to methyl jasmonate

A total of 10 *bHLH* genes were up-regulated by MeJA treatment of *S. miltiorrhiza* leaves, *SmbHLH4*, *SmbHLH8*, *SmbHLH21*, *SmbHLH37*, *SmbHLH51*, *SmbHLH53*, *SmbHLH60*, *SmbHLH74*, *SmbHLH90*, and *SmbHLH109* (Fig. 1), 3 of which (*SmbHLH37*, *SmbHLH51*, and *SmbHLH53*) are in subfamily R. In addition, 4 of the 10 genes (*SmbHLH51*, *SmbHLH53*, *SmbHLH60*, and *SmbHLH74*) exhibited higher expression levels in the root than in the other three organs. These 4 genes are most likely involved in the regulation of tanshinone biosynthesis in *S. miltiorrhiza* and are distributed among 3 subfamilies (subfamilies H, R and W). More interestingly, *SmbHLH74* (subfamily H) exhibited high expression levels not only in the root but also in the periderm. In addition, the expression level of *SmbHLH74* was up-regulated upon MeJA treatment.

### qPCR analysis

**Figure 5:**
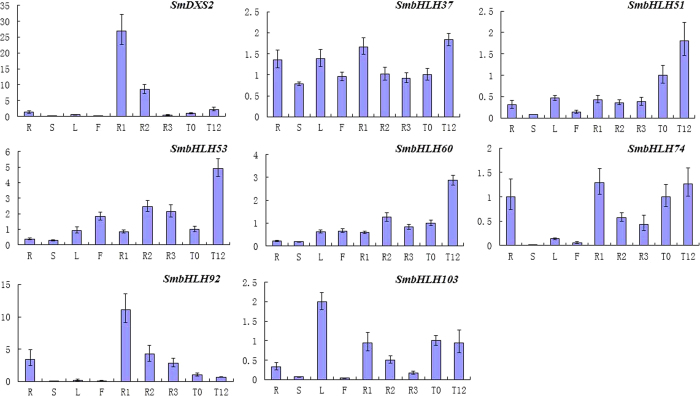
Expression patterns of 7 putative *bHLH* genes and the *DXS2* gene. Fold changes in gene expression levels in *S. miltiorrhiza* R (root), S (stem), L (leaf), F (flower), R1 (periderm), R2 (phloem), R3 (xylem), T0 (treatment with carrier solution for 12 h), and T12 (treatment with MeJA for 12 h) are shown. The transcript levels in the leaf after treatment with the carrier solution for 12 h were normalised to 1.

The expression levels of 7 *bHLH* genes (Fig. 1) identified as putative regulators of tanshinone biosynthesis were analysed using qPCR. The *DXS2* gene exhibited relatively high expression in the root and root periderm. Additionally, the expression level of *DXS2* was up-regulated upon MeJA treatment (Fig. 5). The expression patterns of 3 (*SmbHLH37*, *SmbHLH74*, and *SmbHLH92*) of the 7 genes in various organs or tissues or in response to MeJA treatment were similar to the results obtained from RNA-Seq (Fig. 5). However, the expression patterns of *SmbHLH51*, *SmbHLH53*, *SmbHLH60* and *SmbHLH103* in the four organs did not match those obtained by RNA-Seq (Fig. 5).

### Promoter sequence analysis of enzyme-coding genes in tanshinone biosynthesis pathways

As the biosynthesis of tanshinones depends on cross-talk between the MEP and MVA pathways^21^, the promoter sequences of 72 enzyme-coding genes (19 enzymes) in tanshinone biosynthesis pathways were analysed (Table S6). Interestingly, 70 enzyme-coding genes were predicted based on bHLH binding sites (E-box), including 4 functionally characterised genes (*HMGR2*, *CPS1*, *KSL1* and *CYP76AH1*)^22^,^23^,^24^. The E-box was predicted in the promoter sequences of 6 enzyme-coding genes (*DXS*, *DXR*, *MCT*, *CMK*, *MDS* and *HDR,* but not *HDS*) in the MEP pathway. Additionally, the E-box was predicted in the promoter sequences of genes encoding different types of enzymes (*AACT*, *HMGS*, *HMGR*, *MK*, *PMK* and *MDC*) in the MVA pathway. These results indicated that bHLH transcription factors may play a role in regulating tanshinone biosynthesis via the modulation of the MEP and MVA pathways.

## Discussion

Although the *bHLH* family has been broadly studied in animals and a diverse range of plants, the present study is the first to report the identification and characterisation of *bHLH* transcription factors based on the entire genome sequence of *S. miltiorrhiza*. According to their evolutionary origin, sequence relatedness, functional activity and DNA-binding specificity, bHLH proteins are classified into 6 main groups (designated A to F) in animal systems^25^,^26^,^27^; however, the classification of *bHLH* transcription factors in plants is on-going^28^. In this study, 127 *bHLH* members in *S. miltiorrhiza* were classified into 25 subfamilies, consistent with other plants^4^,^28^,^29^,^30^,^31^. Previous studies have suggested that the classification of *bHLH* transcription factors in plants might be different from that in animals. Based on a phylogenetic tree, *bHLHs* in *S. miltiorrhiza* and *A. thaliana* can be classified into 52 subfamilies (Figure S2); 23 of these 52 subfamilies include proteins from *S. miltiorrhiza* and *A. thaliana*, with the other 10 subfamilies (18 genes) specific to *S. miltiorrhiza* and 19 (48 genes) specific to *A. thaliana*. These results indicate that certain bHLHs play strongly conserved roles in *S. miltiorrhiza* and *A. thaliana*, whereas other bHLHs may have specific functions. Most of the *bHLH* genes specific to *S. miltiorrhiza* were found to cluster in subfamilies R and P. Additionally, 4 of the 7 putative genes regulating tanshinone biosynthesis are specific to *S. miltiorrhiza* (Fig. 1).

The regulation of growth and development, stress resistance, and signal transduction by bHLH transcription factors has been reported in plants^27^,^30^,^32^,^33^,^34^,^35^,^36^,^37^,^38^. To date, at least 43 bHLH transcription factors have been identified as regulators of secondary metabolism in at least 21 distinct plants (Table S7), and these active components include flavonoids, alkaloids, and terpenoids. *bHLH* transcription factor genes are expressed in stimulus-responsive, constitutive or organ-specific manners^39^,^40^,^41^, and organ-specific and MeJA-responsive expression patterns of *bHLHs* were observed in our study. In addition, available functional information from *Zea mays* L., *A. thaliana* and *Matthiola incana* R. Br. suggests that bHLH transcription factors can participate in flavonoid biosynthesis in both a positive and negative fashion^42^,^43^,^44^,^45^. In *Catharanthus roseus* (L.) G. Don, the bHLH transcription factor CrMYC2 regulates *ORCA* gene expression, which in turn regulates alkaloid biosynthesis genes^17^. Additionally, a regulatory role for *Coptis japonica* Makino CjbHLH1 in the transcription of isoquinoline alkaloid (IQA) biosynthesis genes has been reported^16^. The main objectives of the present research were to identify potential bHLH transcription factors involved in tanshinone biosynthesis.

Genes expressed at a higher level in the root and root periderm or in MeJA-treated leaves might regulate tanshinone biosynthesis in *S. miltiorrhiza*. After mapping all of the *bHLH* genes (genes up-regulated in response to MeJA, genes specifically expressed in the root, genes more highly expressed in the root than in the other three organs, genes exhibiting a relatively high expression level in the three tissues, genes more highly expressed in the periderm than in the other two tissues, and genes specific to *S. miltiorrhiza* compared to *A. thaliana*) to the phylogenetic tree of the *bHLH* gene family in *S. miltiorrhiza*, most genes were found to cluster in subfamilies H, P, R and W (Fig. 1). The regulation of sesquiterpene biosynthesis gene expression by *Arabidopsis* MYC2 (bHLH transcription factor) has been reported^46^, and tanshinones and sesquiterpenes are both terpenoids. In addition, upon construction of a neighbour-joining phylogenetic tree using MEGA 5.1, *MYC2* was found in the same subfamily as *SmbHLH37*, *SmbHLH51* and *SmbHLH53*. Based on gene-specific expression patterns, up-regulated expression patterns in response to MeJA treatment and qPCR analyses, the gene expression of 3 *bHLH* genes (*SmbHLH37*, *SmbHLH74*, and *SmbHLH92*) perfectly matches the accumulation pattern of tanshinone biosynthesis. Previous studies have shown that roots contain the highest concentration of tanshinones, though the concentration in leaves is very low^47^, and some key enzyme-coding genes (*SmGGPPS2* and *SmCPS1*) have been reported to be relatively highly expressed in roots^48^. These results indicated that tanshinone biosynthesis was more active in roots than in other organs, further suggesting that tanshinones are originally produced in the roots of *S. miltiorrhiza*. These results provide the foundation for understanding the role of bHLH transcription factors in the regulation of tanshinone biosynthesis in *S. miltiorrhiza*.

## Materials and Methods

### Plant materials and treatment conditions

*Salvia miltiorrhiza* Bunge (line 99–3) was grown in a field at the Institute of Medicinal Plant Development in Beijing. When the plants were blooming in May, the flowers, leaves, stems and roots of *S. miltiorrhiza* were collected, treated with liquid nitrogen and stored at −80 °C until subsequent analysis. In addition, some roots were separated into the periderm, phloem and xylem immediately after collection. Leaves were treated with MeJA (200 μM) and collected as described in a previous study^49^. At least two biological duplicate samples of each organ or tissue were used.

### RNA-Seq and bioinformatics analysis

Total mRNA was isolated using the RNeasy Plus Mini kit (Qiagen, Hilden, Germany); 1% agarose gel electrophoresis was used to confirm the integrity and quality of the total RNA. An accurate RNA concentration was determined using a NanoDrop 2000 spectrophotometer (Thermo, Waltham, MA, USA) and Qubit (Invitrogen, Carlsbad, CA, USA). An Agilent 2100 (Agilent, California, USA) was used to identify the integrity of the total RNA in more detail and to confirm accuracy before sequencing. A total of 19 sequencing libraries were constructed, including 8 for the 4 organs, 9 for the 3 root tissues and 2 for the MeJA treatment experiment^49^. The libraries were sequenced using the Illumina HiSeq 2500 platform to produce 100 bp paired-end reads. All produced reads were mapped to the genome of *S. miltiorrhiza* using Tophat 2.0.11^50^. Differential gene expression was analysed using Cufflinks 2.2.1^51^. To visualise the global transcription profile of *bHLH* genes, hierarchical clustering was performed using R^52^.

### Identification of *bHLH* genes and sequence feature analysis

All *SmbHLH* genes were identified by BLAST analysis of the bHLH domain against the *S. miltiorrhiza* genome. All of the predicted SmbHLH proteins contain the conserved bHLH domain, as determined using sequence search in Pfam^53^. The *SmbHLH* genes were manually corrected using a protein BLAST algorithm (http://blast.ncbi.nlm.nih.gov/Blast.cgi). In addition, the Compute p*I*/Mw tool on the ExPASy server (http://web.expasy.org/compute_pi/) was used to predict the theoretical isoelectric point (p*I*) and the molecular weight (Mw) of the SmbHLH proteins.

### Phylogenetic, gene structure and MEME motif analyses

A Clustal W analysis was performed, and an un-rooted neighbour-joining tree was constructed in MEGA 5.1, with 1000 bootstrap replicates based on the amino acid sequences of the bHLH domain of SmbHLH proteins (Table S8)^54^. The online Gene Structure Display Server (GSDS 2.0) (http://gsds.cbi.pku.edu.cn/index.php) was used to investigate the gene structure based on each coding sequence (CDS) and corresponding genomic sequence. Conserved motifs in *S. miltiorrhiza* bHLH transcription factors were identified using MEME (Suite version 4.9.1) with the following criteria: expected E-values less than 2 × 10^−30^, any number of repetitions of a motif, and an optimum width of 10–200 amino acids^55^,^56^.

### Quantitative real-time reverse transcription PCR (qPCR) and promoter sequences analysis

Total RNA was reverse-transcribed using a FastQuant RT kit (TIANGEN, China). The qPCR reactions were performed according to the manufacturer’s protocol with the SYBR premix Ex Taq kit (TaKaRa, China) and conducted in triplicate using the Applied Biosystems 7500 Real-Time PCR system (Life Technologies, USA). The primers were designed using Primer Premier 6 (Table S9), with an amplicon size ranging from 130 bp to 180 bp and an optimal *Tm* of 54 ± 1 °C. The specificity of the primers was assessed by 2% agarose gel electrophoresis and a dissociation curve. *SmActin* was used as the reference gene in the current study. In addition, the expression level of the *DXS2* (1-deoxy-D-xylulose 5-phosphate synthase) gene, which encodes the enzyme for the first step in the MEP pathway, was analysed as a positive control^48^. The promoter sequences (1500 bp) of enzyme-coding genes in the tanshinone biosynthesis pathways were used to predicted *cis*-elements in the PLACE database (http://www.dna.affrc.go.jp/PLACE/signalscan.html).

### Accession codes

Illumina HiSeq and genome sequencing data have been submitted to Sequence Read Archive (SRA) of the National Center for Biotechnology Information (NCBI) under accessions SRP051564, SRP028388, SRR1640458 and SRP051524.

## Additional Information

**How to cite this article**: Zhang, X. *et al.* Genome-wide characterisation and analysis of bHLH transcription factors related to tanshinone biosynthesis in *Salvia miltiorrhiza*. *Sci. Rep.* **5**, 11244; doi: 10.1038/srep11244 (2015).

## Supplementary Material

Supplementary Information
