# Supplementary material for: Genome-wide characterisation and analysis of bHLH transcription factors related to tanshinone biosynthesis in Salvia miltiorrhiza
Source: Sci Rep. 2015 Jul 15;5:11244. doi: 10.1038/srep11244 (PMC4502395; doi:10.1038/srep11244)
Supplement: Supplementary Information [file srep11244-s1.pdf]

# **Genome-wide characterisation and analysis of bHLH transcription factors related to tanshinone biosynthesis in *Salvia miltiorrhiza***

Xin Zhang[1], Hongmei Luo[1], Zhichao Xu[1], Yingjie Zhu[2], Aijia Ji[1], Jingyuan Song[1,3] & Shilin Chen[1,2]

1. Institute of Medicinal Plant Development, Chinese Academy of Medical Sciences & Peking Union Medical College, Beijing 100193, China, 2. Institute of Chinese Materia Medica, China Academy of Chinese Medical Sciences, Beijing 100700, China, 3. Chongqing Institute of Medicinal Plant Cultivation, Chongqing 408435, China.

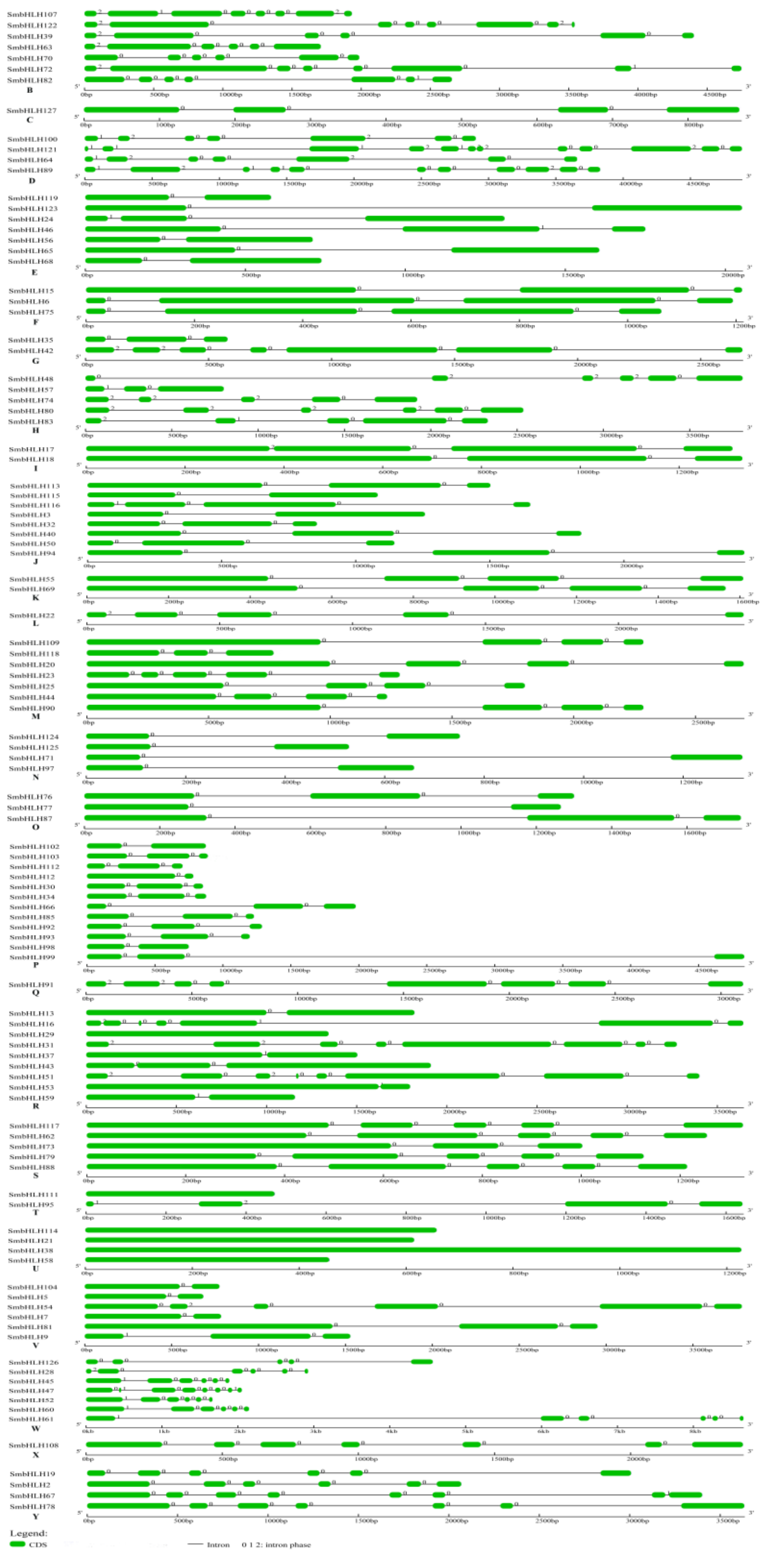

**Figure S1. The structural features of each *bHLH* gene in subfamilies B-Y.** The exons are represented by green round-cornered rectangles. The black lines connecting two exons represent introns. The number above the line represents the intron phases. The letters (B-Y) represent the main subfamilies.

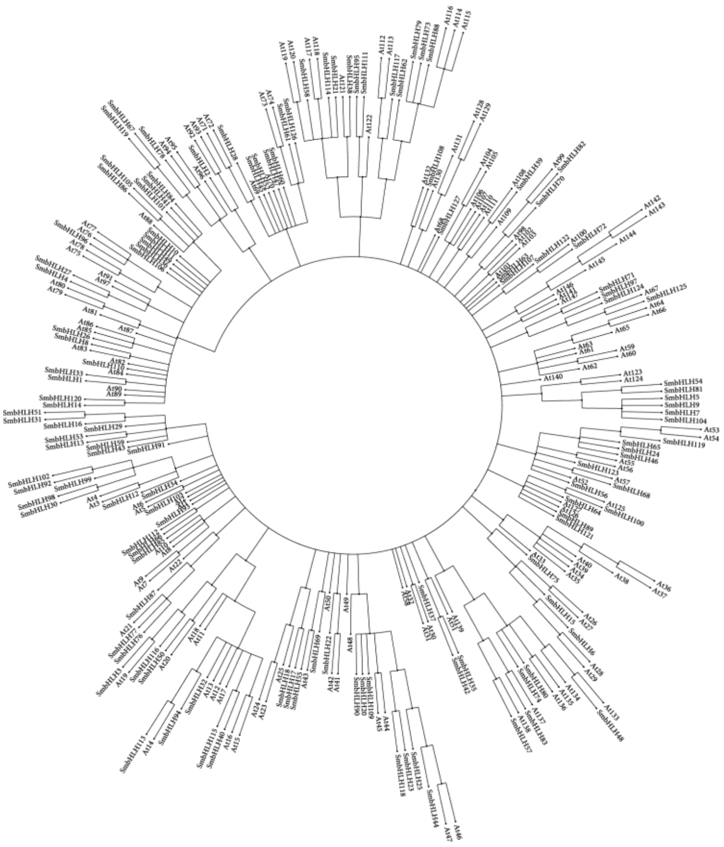

**Figure S2. The un-rooted phylogenetic tree of the *bHLH* gene family in *S. miltiorrhiza* and *A. thaliana*.** A neighbour-joining phylogenetic tree was constructed using MEGA 5.1 based on the bHLH domains of AtbHLHs and SmbHLHs. The *A. thaliana* and *S. miltiorrhiza* protein names begin with “Sm” and “At”, respectively.

**Table S1.** cDNA sequence of *bHLHs* identified in *S. miltiorrhiza*

| **Gene name** | **cDNA sequence of *bHLH* gene** |
|---|---|
| SmbHLH1 | ATGGTGTTTTCTTATTCATGCATCTTGATGCACATTTATAGCCAGCAG<br>AATTTGGTGCTTTCTGCTCCAACTTCAGTGCCCATATTGCCAGGATCA<br>CAAAAAGGGCAGGAATTCTTGCAAGAGAAACGGCATAACAGAGGC<br>AAATTCAAGTGTTGTTCTTCAATCATGGCTGCAGATAATGTTGCAGA<br>AATGTCAATTTTCTCAAAAGTAGACCATTTTTACAGTTGTGGTTGGG<br>ATGCCATGGTTTTGGGGAATGAAACAGCAACTTTTCTTGAAAATCAG<br>ACAAAGTTAAACTCTTCTGATTCTGGTTTAGCTGAAAGCATGCCAAT<br>GATTACTTCAGTTGGAGAAGTCGATGAATCTTGCAGTCACCCAGATT<br>TTTTTCAAGATCGTTGTCAAAGTCCATCTAATGCAAAGAGGAAAAGA<br>AATGTATCTTCATCTAAGAATTCCAAAGTGGATGAAAAGCAAACAGT<br>TGATGCTGAAGAGAAATTTGTTCATATAAGAGCCAAAAAGGGTCAG<br>GCAACAAGCAACCACAGCCTAGCAGAAAGGGTGAGGAGAGAGAGG<br>ATTAGTGAGCGCATGAGATTGCTGCAAGAATTGGTTCCTGGTTGTGA<br>TAAGATTACTGGTAAAGCAGTGGTGCTTGATGAGATTATCAACTACG<br>TGCAATCACTACAACAGCAAGTTGAGTTTCTTTCAATGAAGCTTGCA<br>ACCGTGGATCCAGAGCTCAACCTTGATATTCAACCCACTTTGTCTAC<br>ACATCTTCGAGGTAGCAATGGAGTTGGTGGTACGAGTTCGTCTTGTT<br>CCCAAGGGAATGTTCCTTCGCTACCTCAGGACTTATGGAACAATGAA<br>CTCCAAAGCGTCCTTCAAACTAGATTCTATCCCTGTTCCTCTATGAGC<br>GGTTTGCTGGGAAATGGGTTGTCGGAAATGGAGCTGTAG |
| SmbHLH2 | ATGTCGAACAATCCAGGCGAAGCCCCCTCCGACGATTTCCTCGAGC<br>AGATTTTAGGGTATCAGAACTACGCCGCCGGAGCCGACTCTACTTTG<br>GTCGGAAACGAAGGTCCGTCGCCTGCGATGTTGCTGCAGCTCGGCT<br>CGGGCGACGGCTCGGCTCATCTAGGTGGAGTCGGCGGCATGGGAAT<br>CGGAATAGGCGGCGGTTACGGCGTCGGAGGTGGCGGCGGCGGGTTT<br>CCGCTGGGACTGAGCTTGGAGCAGGGCAAGGGAGGGTACAACGAC<br>GCATCGGGTAGCGGGAAGAGGTTCCACCGCGATGATTTTGTTGATTC<br>CCGCGGTGCTGCCATGAAAGGGGGTTTTCATGGACAGCCGATGATG<br>AACACTGTACAGGCAGCGCCCCATCCGCCTGGAATTCGTCCAAGGG<br>TACGGGCCAGACGAGGCCAAGCGACTGATCCACACAGTATAGCTGA<br>AAGGTTGCGGAGAGAAAGAATAGCGGAAAGAATAAGAGCATTGCA<br>AGATTTAGTTCCCAGTGTCAACAAGACAGACAGAGCAGCTATGCTT<br>GATGAAATCGTGGATTACGTCAAGTTCTTAAGGCTCCAAGTGAAGGT<br>GTTGAGCATGAGTAGATTGGGAGGAGCTGGTGCAGTGGCTCCGCTT<br>GTTACAGACATCCCAATATCATCAGTAGAGGAAGAATGCAGTGACGG<br>CGGAAGAGCTCAGCCGGCTTGGGATAAATGGTCAAATGATGGCACA<br>GAGAGGCAAGTAGCTAAGCTTATGGAAGAAAATGTTGGAGCTGCGA<br>TGCAGTTTCTTCAATCCAAGGCGCTCTGA |
| SmbHLH3 | ATGGATGCTGAAACATTCTTCGACGATACCGATATCTTCAGCGTTCTC<br>GAAGCATGGGAGAACGAGAGCTTCCACTTCCAAACCTCGCCCTCCA<br>TTCTTAAGAGACCTGCCGAGGTGGAGGCCGAGCTCGACGCCGACGC<br>CTGCAAGCGCCACAAGCTCACTGAAGAAGCTGCCGGCCACAACAA |

| | |
|---|---|
| | AGTGTCGCACATCACCGTCGAGAGAAACCGCCGGAAGCAAATGAAC |
| | GAGCATCTCTCCGTGCTTCGCTCCTTGATGCCTTGTTTCTATGTCAAA |
| | AGAGGCGACCAAGCATCGATAATAGGCGGAGTGGTGGATTACATCA |
| | ACGAGCTGCAGCAGGTGCTGCAATCTTTGGAAGCCAAGAAACAGCG |
| | CAAAGCAGCTTACAACGAAGTAGTGGTGAGCCCGGGCCCGAGGATA |
| | GTGTCGAGCCCGAGGCTGAGCCCGAGGACCCCACAGCCGCAGCCA |
| | AGCAGCCCCTACAATTACAAACCCTGGTCTCTCCCCAGCCCCGTCGA |
| | TCCTTCTCCCTCCAATTCTTCTACCTCCTCTTCCATAAACGACGCCGT |
| | CAATGAGCTCGTCGCCACCTCCAAATCACCCATAGCTCAAGTCGAGG |
| | TCAAGTTTTCCGGCCCCAATCTGCTGCTCAAGACAGCTTCGCACCGC |
| | ATCCCGGGCCAAGTGCTCAAGATCATTTCTGCACTCGAACAACTCGC |
| | CCTCGAAATCCTACACGTCAGCATCAGCACCCTCGATCATGCAATGC |
| | TCAATTCTTTCACCATTAAGGTAATTAATGCGACTTCCATTTCCACTTA |
| | TTATTTAAATTTAAATAATACTAATAATAATATCGAAGATGAAGATTAA |
| SmbHLH4 | ATGGAGCCAAATGTTGAAGCGGTTCAACCGCAATTTAGATCCGGAAT |
| | ATCCGGAGCCAAACCAGGAGGAATGGGGTGCGTGTTCGGTGACATC |
| | CACGGCCTGATCCCCGGTCCACCGGAAACGGACGGCACATCTTTCA |
| | CGGCGCTTCTGGAGCTCCCACCGCCGCAAGCTGTGGAGCTCTTGGT |
| | CAAGGAAGATTTTCCGGCGAAACCTATGCCGCCGCCAATTTTTCCTT |
| | CCAATATTGCCCTCATTGACCGCGCCTCAATGTTTTCTGTTTTTGCTT |
| | CGGCCGATAATCCGCCGGAGAGCACCAACATTTTGTCGGCTTCGAGC |
| | TCAATGAAGATCGATTCGGTGAAGCAAGAGCCCTTGGAGTCAGAGA |
| | ATCAACGGAATTCTTCTTCGCCGGCGGTTTCGAATCAGAGCTTGAAG |
| | TCCATCAAGCGAAAGGAGAGGGAGAAAAAGGTGAAAGAATCAAGC |
| | AAAAAGAGCAAAAAAGTGGGAGCAAATGAAGCGTCTGATGGGAGT |
| | GGCGAGCAGCTGCCGTACGTTCATGTCAGAGCTCGCCGTGGCCAGG |
| | CCACTGACAGCCATAGCTTAGCTGAAAGAGCCCGGAGAGAGAAAAT |
| | CAATGCCAGGATGAAGCTATTACAGGACCTGGTCCCGGGATGTAACA |
| | AGATTTCAGGTACTGCAATGGTGCTCGATGAGATAATAAATCACGTG |
| | CAAGCACTTCAACGTCAAGTGGAGTTTTTATCCATGAGACTTGCTGC |
| | TGTTAACCCAAGAATTGATTTCAACCTCGACGCCCTCTTAGCCGTAG |
| | AAAGTGAATCTTCCATTGATAATAGCTACCTAGGCATGTTTCCACCAT |
| | CCATTTGGCCGGAGGGACAAATCAACGGCAGCAGACAACAACAGTA |
| | TCAACAGCCATGGCTCATGGACCCGCCTTCCATGGGCAGAGTAGAC |
| | GACACCTCTAACTTCATTACCCCGGAGAACTCACTGCTAAGTTACGA |
| | CTCTCCAGCAAATTCAGCTTCTCTGCACTCGAGCCAGCTGAAAATGG |
| | AGCTTTGA |
| SmbHLH5 | ATGGCGTTGAGCTACTACACCTTTCAGCAGCCAGACTACGATCCAGA |
| | GCTCCAATCTCTGTTGAACCCGGACGACTACTCTCTTCTCTGCGAGG |
| | ACCTCACTTATCATGCTAATGGACTTCCCTTGGATTTGGATAATCTCA |
| | CAACTCCCTTTCTTTTGCCGCACCAACAACAACAATCCCAGGCGCCC |
| | TTCCCCAAACGCCACAAGCTCTCGCTGCCGGAATTTGTCTCGTTGCC |
| | TCAGTTCCCTGCGGCGGATTTCAGCTGTGAGAGCGCCAAGAACGAA |
| | ACGAGCTTATCCGCGCAGAGCATCGCAGCCAGGCAGAGGCGGCGGA |

AGATCACGCTGAAGACGCAGGAGCTCGGAAAGCTGGTTCCCGGCG
GGCAGAGGATGAACACGGCGGAGATGCTGCAATCCGCCTACAACTA
CATCAAGTTCTTGCAGGCGCAAGTTGCTCTCCTTGGTTCGCATCATC
AGGAGGTACCATTCGAAGGTGAAGAAGAGCTCCAGAATCTTCTAGA
ATCTCCTTTGATTCAAGAAAAGCTATACTCCACTCAACATTGCTTGCT
TCCAATTCCCATTTGCTGGAAAAGGATCATTGATGACTCACGCCCGC
TGCCTAGCTAG

SmbHLH6 ATGGAGATTTCAACCTTCAGAAACATAGCTGATTTCGGAATGGATGA
TCCTCTATTCTTCCAGCAATGGCAAGTGGATCCAATGGAAGATTTCA
GCTCGATTTCTTGTGCATTTTCCGAAGGCTTTCATCAGTCCTACACCC
AGCAGCCTCTGCTGGACTTCAAGAGGCCCGCGGAGGCGTCTCCGGC
GGGCGAAAGCCGGCCATCGAAGCAGCTGAGAACCTCAAACTGGTG
CAAGCAAGAAAGAATGTCAAACTCACCCCCTCCAACCAGTCCTATT
GTGGGGGTTGTGAGGCCTCTTAAAGAGGAAGCATGGTTCTCTCCTTC
CACTCTCACTGATAATCAATCTTCAAACTATGTGCTGAAGCCATGCCA
AGGGATCAAGAGAGTCACCCCCAACACCAAGCTCTCTCAAGCACAA
GACCACATCTTAGCTGAGAGAAAGAGGAGAGAGAAGCTCAGCCAG
AGGTTCATCGCCTTGTCTGCCCTAGTCCCCGGTCTTAAAAAGATGGA
TAAGGCTTCTGTTCTTGGAGATGCTATCAAGTACATGAAGCAGCTCC
AGGAAAAGGTGAAGGCGCTGGAGGAGAAGGCGAAGAAGACGACG
GCTGTGGAATCCGTGGTGTTGGTGAAGAAATACCAAGTGTGCGATCT
GGATTCCTCAACGGATCTCTGTGAATCGCTCCCCGAGATTGAAGCGA
GGTTCTGTGAGAAAGATGTGCTGATTAGCATCCACTGTGAGAAGAG
AAAAGGGGTTTTGGAGAAAATCGTTGCTGAGATTGAGAAGAAGCTC
CATTTATCAGTTGTCAACAGCAGCGTCATGACTTTCGGAGATTCTGC
TCTCAACATAACAATAATTGCACAGAGGGATGATGAATCCAGTCTGG
ATATGAAGGAACTGGTGAAGAATTTACGTGGTGCTCTCAGAATATGA

SmbHLH7 ATGGCGTTGAGCTACTACTCCAACTGGACCTCTTTTCAGCAGCCAGA
CTACTCCGGCGATGATCCAGAGCTCCAATCTCTGTTGAACCCGGACA
ACTATTTCGCCGACTCCTTCTGCAACTCTCTTCTCTGCGACGACCTC
ACTTATCATGCTAATGGACTTCCCTTGGAATTGGATAATCTCACAACT
GCAACCCAACCCCCACCCTTTCTTTTGCCGCACCAACAACCCTTGTT
CCACTTCCCCAAACGCCACAAGCTCTACGATTACTCGCTGCCGGAAT
TTGTCTTGCCGCCGCCGTTGCCTCAGTTCCCTGCCGCGGATTTCAGC
ACCAGGATCTGTGAGAGCGCCAAGAACGAAACGAGCTTATCCGCGC
AGAGCATCGCAGCCAGGCAGAGGCGGCGGAAGATCACAGTGAAGA
CGCAGGAGCTCGGGAAGCTGGTTCCCGGCGGGCAGAGGATGAACA
CGGCGGAGATGCTGCAATCCGCCTACAACTACATCAAGTTCTTGCAG
GCGCAAGTTGCTCTCCTTGAATTCCTTGGTTCGCATCATCAGGAGGT
ACCATTCGAAGGTGAAGAAGAGCTCCAGAATCTTCTAGAATCTCCTT
TGATTCAAGAAAAGCTATACTCCACTCAACATTGCTTGCTTCCAAAC
AAGTTGGCGGAACAAGTTCCATTACTCAAATCCAATCCACATTTGCT
GGAAAAGGATCATTGA

SmbHLH8 ATGGAGAAAGAGGGCAGCAGCAACATCTTTTTCAGACCCAATTGGG

AGAATTCAGTGGATCAGAACGATCCCTTTGAGTCCGCATTGAGCTCC
ATGATTAGGCAGCATTTGCAATTCCGGCGAGATTTCCCTTTCAATGGA
AACAACAACAACAACAACAGCAGTGCTAACACCTCTTGCTACAGCA
CCCCTCTCAATTCCCCTCCCAAGCTCAACCTCTCCTCGATCATGAGA
GACAGAGCCAATCACTTTCTCTCTCTTCCCACCCTCGCTCCCTTCTCT
GCTGATCCAGCCTTTGCCGAGCGTGCCGCCAGATTTTCTTGCTTCGC
CAACAAGAATTTCAATGAACTCGATCCCACTAAGATTCCCCCGCCAC
CAACAGTTCAAGAAAACTGGGAATCAATTCAAGCTCCCGAGAAGAA
TTTCATGGCTTCCAGATCTTCCACGCCAGATAATGCCGCGGATTCGA
GGGAGAATTCCACGGTTTCCGAGCAGATCCCACCTCCCGAAAATGG
CGGAAATCCCAGAAAGAGAAAATCGGCTCCCAAAGGAAAGGGGAA
AGACACCACCACCACCGCCCCTGCTCCAAATCTTGCCAATGTTTCTG
CATCGGAGAATAGCGAATCCAGCGCCAAGAGAAGCAAATCGGAAGA
AGACAAGGCCAATCAGAAGCCGTCCAAGGATAATTCGAAGCTTCCA
GAACCTCCCAAGGACTATATCCATGTCCGAGCTCGACGCGGCCAAGC
TACAGACGCCCATAGCCTCGCAGAAAGAGTTCGTAGAGAGAAGATC
AGTGAGAGGATGAAAGCGCTACAAGATCTTGTACCAGGTTGCAATA
AGGTGACTGGGAAAGCCGTTATGCTTGATGAGATCATTAATTATGTG
CAGTCTTTACAGCGGCAAGTTGAGTTCCTTTCAATGAAGCTGGCTAC
TGTGAATCCAAGAATGGATTTCAACATGGAAGCACTAATGTCTAAGG
ATATGTTTCAGTCTCGTGGCTCGTTGCCTCATAGCGTGTACTCATCGG
ATGGCTATCCGTTTCAATCTCAGTCGATCCCATGTGGGAACGAGGCG
CCTTTCGCGTCTAGCAATGCTGCAGCTAGAAACCAAATGGAGAATTT
TGGTGATGCTGCCTCTCAAGTTTCATCGTTTTGGGAAGATGATCTGC
ATAGCATTGTGCAGATGGGATTTGGCCAGATGCAAAATTTTCAAGGG
ATTCTTCCCACAGCTCAAATGAAAGTTGAGCTATAA

SmbHLH9 ATGGGTTGCGCTGGGCAGTGCTCAGGGGCAGAGCTGCGTTGTGTAG
CGCTCTGGGGCAGGGCTGCGCTGAGCAGCGCTCTGAGGCAGAGCA
ACGCTCCGCGGCAGGGCTGCACTGAGCAGCGCTCCGGGGCAGGGC
TGCACAGAGCAGCGCTCCGGCGCAGGGCTGCATTGAGAAGTGCTTC
CGGGGCACGCTGCACTGGGCTGTCGAGCGTTTCCCGGCAGGGTAAT
CCCTTCATCAATGGCGTTGAGCTACTACTCCAACTGGACCTCTTTTCT
GCAGCCAGACTACCCGGCGATGATCCAGAGCTCCAATCTTTTTTGAA
CCCGGACGACTATTTCGCTGACTCCTTCTGCTACTCTCTTCTCTGCGA
CGACCTCACTTATCATGCTAATGGACTTCCCTTGGATTGGATAATCTC
ACAACTCAACCCAACCCCCACCTTTTCTTTTGCCGCACCAACAACA
ACAATCCCAGGCGCCCTTGTTCCACTTCCCAAAGCCACAAGCTCTAC
GATTACTCCTGCCGAATTTGTCTTGCCGCCGCTGTTGCCTCAGTTCCC
TGCCGCGGATTTCAGCACGGGGAGCTGTGAGAGCGCCAAGAACGA
AACAAGCTTATCGCGCAGAGCATGCAGCAGGCAGAGGCGGCGGAA
GATCACAGTGAAGACGCAGGAACTCGGGAAGCTGGTTCCCGGCGG
GCAGAGGATGAACACGGCGGAGATGCTGCAATCCGCCTACAACTAC
ATCAAGTTCTTGCAGGCGCAAGTTGCTCTCCTTGAATTCCTTGGTTC
GCATCATCAGGAGGTACCATTCGAGGGTGAAGAAGAGCTCCAGAAT

CTTTTAGAATCTCCTTTGATTCAAGAAAAGCTATACTCCACTCAACAT
TGCTTGCTTCCAAACAAGTTGGCGGAACAAGTTCCATTACTCAAATC
CAATCCACATTTGCTGGAAAAGGATCATTGA

SmbHLH10 ATGTACTCCCAATCGGAGCCGGAACGTGTTGTTCATCACCGGTCTTA
CGTCTACCGTGATCGTGAGGCTACCCATTTATGTCTACCATTGTTCTT
GCTCATCGTCGATGATGTGCAAGAATTTATTGGACTCGAGAAGCCAT
TCATCGAGTATTCCTTTCAGGACTATAATGCTGGACTGTCCCTCTATG
GAGATAAAGCGAAGCCGAAGACTTATGCTGCGCGTTTCGCGACTCC
TGATGGACATGCATCCTTCCAGCTATTAGGAGGTGTTGTGGGTGCTC
TTGTTTATATCTTCCCAGAATTCGTGATTCAAATTTATGCACCGGGTTT
GTGGATATTGGCTGAAGGCCAAATCATAAGGGAAATAGCTATCAAGC
AGAATGAACGAAGGAATGAAGGTGATAGTAAACGGATGAAGGCAGT
GGGTGAACTTAAAGTGGAAGGGGAAGGGAATTCCGGCAAGGGGGC
GGCCGCGGTGGAGAGACGCTCGAAGCTAGACGAACTGCCGAAGCA
AGACTACATCCATGTTCGAGCAAGACGAGGTCAAGCTACCGATAGTC
ATAGTTTAGCAGAAAGAGCTAGGAGAGAAAAGATTAGTGAGAGGAT
GAAAATTCTTCAGGATTTGGTTCCTGGTTGTAATAAGGTCCGAGCTG
CTATCAAGGTCAAGGCATCAGTCATCATCTGTTTCACTTCTTCCAGG
AAGAGCTGCTAG

SmbHLH11 ATGTTGACGGATCAGAGATCGAACGACGATTCGCGGAAGCGGCGGG
ATGGAGATGAGCATTGCGCCAAGGGAGGAGGAGCTTCCACCAGCAG
CAGCAATGGCGGCAGCTGCAACAACAATAACGTCTCGAATGAACGA
AGGAATGAAGGTGATAGTAAACGGATGAAGGCAGTGGGTGAACTTA
AAGTGGAAGGGGAAGGGAATTCCGGCAAGGGGGCGGCCGCGGTGG
AGAGATGCTCGAAGCTAGACGAACTGCCGAAGCAAGACTACATCCA
TGTTCGAGCAAGACGAGGTCAAGCTACCGATAGTCATAGTTTAGCAG
AAAGAGCTAGGAGAGAAAAGATTAGTGAGAGGATGAAAATTCTTCA
GGATTTGGTTCCTGGTTGTAATAAGGTCCGAGCTGCTATCAAGGTCA
AGGCATCAGTCATCAACTGTTTCACTTCTTCCAGGAAGAGCTGCTAG

SmbHLH12 ATGAGCTGCTTTTCCGAAATGATTCTTGAACAAGATTTGACGATATCG
AATGAGGCTGTTTTGGAAAGCCCTCAACTTGTAACGAAAAGCTCGA
AAAAGAGGCGAAGAACATCAAAATCAGGTCCCAAACAAAACGAAG
AAAACAAAGTAAGCGATGAGCTGAGGAAAACTGCGCATAGAGAAAT
TGAGAGGCAAAGAAGGCAAGAAATGTCTACTCTTTACGCCTCACTT
CGAGAACTTCTCCCTCTTGAATACATCAAGGTATATATATGTTTCTTTC
CTTTTAAATATTATTTAAAGAAGCCCTAAATCAATCTACATGGCAGG
GGAAACGGTCGGTCTCTGATCACATGCACGAGGCTGCAAGTTACATA
AAGGATATGGAGAAGAAGATAGAGGAATTGCAGTTGCGGAGAGATG
AGCTGAAAAATGCATCGGCTTCAAATACTACTTTTGATCTGCCAAATT
TTGTAACGGTGAATTGTTGTTGTGATGGGCTTGAGATTTTGATCAACT
GTGGTGTCAAAGAGGGTTATGGATTTTCCCTATCAAGAGTGATTGTG
GAATTGACTCACAACGGACTCGATGTAGTCACCTGCATTTCAAACAA
AGTGAATGGCAGATTCTTGTACAAAATCCATACTCAGGTAAATGCTT
TGGCATATATTAATCTGGCTGAGCTGCAGCAGAGGCTCGCGTATGAG

ATCAATTAA

SmbHLH13 ATGGGGGTTGTTGGTTGGAGTAATGAAGATAAGGCGATGGCAGCAG
CGGTTTTGGGAACTAAGGCTTTTGATTACTTGATGTCGAGCTCAGTT
TCTGCTGAATGCTCGTTAATGGCGATGGGGAGTGATGAGAATTTGCA
GAATAAGCTTGCAGATCTTGTAGAGCGCCCAAATTCTTCCAATTTTTG
TTGGAATTATGCAATTTTCTGGCAGCTCTCTAGGTCCAAGGCTGGGG
ACTTGGTGTTGGGATGGGGGGACGGGTGTTGTCGAGAACCTCGTGA
TGATGAGGAATCTGAGGTTACTCGAATTCTCAAAATGAGGCTCGAGG
ATGAATCTCAGCAGACAATGAGGAAAAGGGTTCTCCAGAGGTTGCA
TACTTTGTTTGGAGGAGGCGATGAGGAAAATTATGCTTTTGGATTGG
ATAAGGTAACGGACACTGAGATGTTCTTCCTGGCATCAATGTACTTTT
CCTTCCCCAGGGGCGAAGGAGGCCCTGGGAGGTGTTTTGGATCTGG
CAAGTATGTCTGGTTGTCGGATTCATTGAAGTCTTCCGTTGATTACTG
TGTTAGGTCATTCCTTGCAAAGTCGGCTGGCATGCAAACTATTGTTTT
GATCCCAACTGACGTTGGGGTAGTTGAATTAGGGTCGGTCAGATGCA
TCCCGGAAAGTATGGAGCTAGTCAAGGTGGTTGGATCTTCCTTTTCG
TCGTTTTCTTCACTCCTCAGGTCGAAACAAGCTGCAGCTGCGGCTGT
GGTGACAGTAACGGACAAAAAGGATACGAATGCCCCCATTCCTAAC
CTGGCTATTGGTAACCGACCAGAAGTTGCTCCCAAGATTTTCGGGCA
GGACTTAAATTCAAGCCATGTGCAATTTAGGGAAAATGTTTCCGTTA
GGAAACCAGAAGTGCAAGAAAGGACTTGGGACGCAAGTGGAAACA
GGAACAGGCTACCATTTACCAACAATCGCAATGGTTTTCATGGCCTA
CGTGGACACAGTATACATCAAAAGCCAGCTCAAATGCACATAGATTT
TACTGGAGCGACCTCAAGGCCTTTAACCTCCTCACATCCACAGAGTC
TCGAATCTGAGCTTTCAGATGTTGAGGCTTCATGCAAGGAAGAGGCT
GCAGGCCTATCAGAAGATAAGAGGCCGAGGAAGCGTGGTAGGAAG
CCTGCCAATGGAAGAGACGAACCCCTCAATCATGTAGAGGCAGAAA
GACAGCGAAGAGAGAAGCTGAACCAGCGCTTCTACGCTCTACGAGC
TGTTGTACCAAATATCTCTAAGATGGATAAAGCTTCCCTCCTTGGAGA
TGCTATTGCTTACATAACCGAACTGCAGAAGAAGCTCAAGGACATGG
AGTCTGAGAGAGAGAGACTTGGCAGCATATCGAGAGAAGCATCTGT
TTCAGAAGCTAATTCTAACAGAGAATCACAAGACCTGCTGGCTTCGA
GCATTAACATCGAAGCTGGCCGTGAAGAAGTCACTGTTAGGATTAGC
TGCCCGCTGGACGCCCATCCAGCATCAAGAGTCATCCAAGCAATCA
ATGATGCACAGGCAACTATCATCGATGCAAAAATGGCTACAGGGAGT
GAGCGAGTGTTCCACACATTCGTTGTCAAATCCCATGGATCCGAACG
ACTAACTAAGGAGAAGCTGATCGAAGCGTTTTCTCGTAGATCCAGCT
CTCCCCATCAGTTATCTCTCGGGTAA

SmbHLH14 ATGGCAGCTTTTTCATCATCATTTCAGCATCTCGACGCATCATCATCAT
CAGTCTTCCTGTCAAGGCCAAACTCCTCTTCATCTTCGTCTCCCTCC
ACCACCATTAATAGCATGTGTGCCCTTTTCCATGATCCAAACAATGCC
TTATCCCATCAATTCCACCACCTTCATCATGGTACAACACCCTCTTCC
TCAAATAATAATATCAATGATTCTGCCCCTTTAAATGCTGCTGCTGCTA
CCAACAAGAACAGCATGGATTCCTCAATATCTGTCGTCACCCACAAA

AACCGCCAAACCAAGAAGAGGAAATCCAATTCTGCTCAGTCCAAGG
ATATGAGAGAATTAGTGAAAGGGAAGAAGCAGAAGAAAGTGAAAG
ATTGTGAAGAGAAGAAAGAAGGTGAAGAAAAAGGTTATATTCATGT
AAGAGCAAGGAGGGGCCAAGCAACTGATAGCCACAGCCTTGCTGA
GAGGGTGAGGAGGGAGAGAATTAGTGAGAGAATGAAGCTACTACA
AGCTCTTGTTCCTGGTTGTGACAAGGCCCTCATGTTGGATGAAATAA
TAAACTATGTCCAATCACTCCAAAATCAAGTTGAGTTTCTTTCAATGA
AGCTTGCATCTGTGAATCCCATCTTCTATGATTTTGGTATGGACTTGG
AAGCATTCATGGTAGGACCTGATCATCAGAATTTAAATAGCTTGGCAT
CACCATTGCCAGCAGGCATGCAAGAATGCAGCCCCACAACAGCCAA
TACCTATCATGTCTTAGATAATTCTGTTAATTCTCTTCTCTTACACCAA
TCCCAAATTCCAAATGCTCTACCCCAGGGTGATAGACAGGTTTTGTG
GGAAGTGGATGAGCAAAGACAGAAAAATATTAATCAGTCAGGAATC
ATCAACAACTTGTTTTCTTTCCCATTGATGTAA

SmbHLH15 ATGGAAATATTGGAAGACGATTTTTTCTATCTCAACCCTTTTCTCGCT
GAGATCTACTCCAACTGCAGCAACAGCGGCAGCTCCCAGACTTCCG
ATCACGGCGGTGCCGCCGATCAGCTGAGAATGAGTTCAACGAGCTG
GATTAATTCATTGAACGGTCGAGAAATGGCGATGCCGAGTTCTTCAA
TAATGGCAGCTGCTGTTGAAGCTCCACGGTTAATTTCTTTTGAGAAC
GAGAGATTTTCGTCGTTTTCTCCGACAAATGCAGATCATCGTAGTAC
AGATTGTAATAACCTAACCCCACAGATCATAAGCTTCTCGTCTTCTAC
TAAAGACGACGACGACGACGGCGATTTCGGTGGTAAGAGAGAGAG
GGCTTCCACGGCGACGCGGACGCCGTTGCAAGCTCAAGACCATCTC
ATGGCGGAGAGGAAACGGCGTGAGGATTTACGTCACCTCTTCATCG
CTCTCTCCAAAGTTGTGCCGGGATTGAAGAAGTTGGACAAGGCATC
TTTACTGGAAGATGCTATAAACCACTTGAAATCACTTGAAGAAAGAG
TGAACGTTCTAGAGAAGGAAGCAATGATGAGTCCAAGAGAAGACAT
CTCGAATAGTGGGTGCGAGTCAGTACCCGTGGAAATAAGTGCAAGA
GTTAGGGAGAAGCATGTCCTAATAAAAATGTGTTGCAAGAAACAAA
TGGGGTTGATGTCAAGAATCCCATGTGAGATGGAAAAATTACATCTA
AATGTGGTAGACATTAGAATCATGCCGTTTGGACAAGCAGCTCTAGA
TGTCACAATTCTTGCTGAGAATAAGCCCTCTTTATGA

SmbHLH16 ATGTTTGGAATTTGGGATAAAAACAAGAATAATAAAGCGACATATATA
ATTGCGATAATGATAGTATTATATAGTTTGTGTTGGTTACCTGGGAAG
GCATATGTAGATAGGCAGCACATTTGGCTTACAAGAGCAAATAAGGC
CGACAGCACACTCTTCTCCAGAACAATTCTTGCTGAGAGTGCTAAA
ATACAGACAGTGGTATGCATTCCTCTTCTGGATGGGGTTGTGGAACT
TGGGACAACACAGAGGGTTGAAGAAGACATTGGATTGATCCAACGT
GTGAAGAGCTTCTTCTCCCACTGTCAAAACCCTAAACCTTCAAGGC
AAGCCCTATCGGGCCACTCCACTTCTAACCCTACCTCTTCGGCCCCC
GCTTATTATTCTCCTCCGCCATTTGCACCACCACAAGTGGGGGAGAA
AGAGGAAGAGGAAGAAGAAGAAGAAGAAGAAGAAGAGGTGGAGT
CCGACTCAGATCAAAACCCTGTTGAAGCAATGCAGCTTGATATGTCT
GACCAGGATATTAGGGCTGGCTCACCGGATGATAGCGAAAATAATTT

AGGCTCCAATTTCCACCACCTCGCCGCCAGCCACGGTGGCTTAGATA
CGGCTGCAACGATGCTTAGGTGGCCATGCAAAAACCCCATCGTCAA
CGCCACCCTTCAACCACCGCATTCAGTGGACATAATAGCAGAAGAA
GACACCCACTATTCTCAGACAGTGTCAAGCCTCCTCGAAAACCAGT
CAAACCGGTGGTCGAAGCTCTCCTCCGCCTCCGCCTCCGCCTCCGCC
TTCTCCAAGTGGCAGGCCGCTTCTTCCAGCCGCCGCCACAACCAAA
TCCTGGGCGGAGCCTCCCAATGGGTCCTGAAATACATACTCTTCAGC
CTCCCGCTCCTCCACACCAAGCCCCGCGACGACCTCGGCGGCAACC
ACGTGCTGGCGGAGCGGCGGCGCCGCGAGAAGCTCAACGAGCGCT
TCGTGGTCCTCCGATCCATGGTGCCCTTTGTGACGAAGATGGACAAG
GCGTCCATCCTCGCGGACACCATCGAGTACCTGAAGCAGCTCAAGG
AGAGAATCGAGCAGCTGGAAGCAGAGAGGAAGGGCGGCCGGAGG
GAGAAGAGGAAGCTGAGGATGGCGGAGGCAGTGGAGGTGTCCATC
ATAGAGAGTGATGCGTTGGTGGAGATTTCATGCTTGCATAGAGGGGG
ATTGTTGTTGGATGTGATGCAGATGCTTCGAGGGTTAGGTATACATGT
CACCACGGTGCAATCTTCCATCAATAATGCCACTTTTTATGCTGAGTT
GAGAGCTAAGGTGACGGAGAGTGCTAACGGCCGGAGACCAAGCAT
CATGGAGGTCAAAACCACGATAAAGCAAATAATTCAACACCAGATA
CAATCATAG

SmbHLH17 ATGTATGAAGAAAACAGTGGTTTGGATGAAAGCAGCAGTATGCATGT
AGATAGAGGTGGATTGAGTCAAGGTGAGGATGCTTTCTCCCAAACC
CACTGCAACAACTTCATCACGGAGGAGCAATCCTACCCTCAAAACG
ACGTCGCCGCCCTAGAGATGGAGCTGCAGATCGAGCAGTGCTTCAC
CAACAACAACAAAATTCATGATATGGCGCACCACGCCTCTAATTGGC
AAGACATCACCACTGGCCATTACCAAATCTTCGCTGCTCAAGCTTCA
TTCACCGACGCCCCCGATTTGCCGGCCATATTTCCTCCTCCGTCTTCG
CTGCTTCCCAACTTTCCCCAAAAAACCCCAGCTTTCTCCGCTTCCGT
CGTTTACGACCCTCTGTTGGCGTTGAATAAGCCCCCTTCGTTCAGAG
ATCTGTTCTATGGCGGTGGGGTTTACACACTTGGGGTTGATGAGAGA
GAAGCAAATGTGGGTTTCTATGAAAACGGTGATGGAGTTTTTGAGTT
CACAGCAGCAGAAATGGGCGAGAATGGAGATGCTATTAAGGATACC
AAACAACACTCGAGCGAGAAGCGTAGGAGAGTGGAGTTGAAGGGC
AAGTATGAATCTCTGCGATTACTGATTCCTAGTCCCTCTAAGAATGAC
AAAGCGTCGGTCCTGGCCGACGCCATTCGCTACATCAAGGAGCTGA
AGAGGACTGTGACTGAGCTCAAAGATCTCGTAGAGAGAAAGAGATT
TGCGAGAGAGAGAGGCTCAAGAGGCCTAAGGTGGAAGAAGATGACGT
GGATCCTTACAATGGATCATCGTTGCGGAGCTCGTGGCTGCATCGCA
AGTCCAAGAACACCGAGGTCGATGTCCGAATCGTAGAAGACGAGAT
CACGGTGAAGCTGGTGCAGCAGAAGCGGATCAACTGCTTGCTGTTT
GTTTCCAAGGTGCATGATGAATTGCAGCTCGATCTTCAACATGTTGC
TGGAGGCCTCATTGGTGATTATTATAGTTACCTTTTCAACTCCAAGAT
TTGCGAGGGGTCGATTGTGTACGCGCGTGCCGTAGCGACCAAGCTG
ATTGAAGTTGTGGACCAACACTATGCTGCACTCCCACCAACAACAA
CCTATTAG

SmbHLH18 ATGTATGAAGAAAACAATGGTTTTGATGAAAGCAGCAGTATGCATGG
AGGTGGATTCAGTGAAGCTGAGGATGCTTTCTCCCAAACCCACTGC
AACAACTTCACCACACAGGAGCAACCCTACCCTCAAAACGACGTCG
CCGCTCTAGAGATGGAGCTGCAGATCGAGCAGTGCTTCACCAACAA
CAACAAAATTCATGAAATGGTTCACCAAGCCTCTCACTGGCAAGAC
ATCACCACTGGCCATTACCAGAATTCTGCAGCAGATTTCAACCAAGT
CTTCGCTGCTAAAGCTTCATTCACCCACCCCCCCGATTTGCCGACCA
TATTTCCTCCGCCGTCTTCGCTGCTTCCCAACTTTCCCCAAAAATCCC
CAGCTTTCTCCGCTTCGTCGAGCAGCATCGTTTACGACCCTCTGCTG
GCATTGAATCAGCCCCCTTCGTTCAGAGATCTGTTCTATTCTTCGCCA
AATGGCGGTGGGGTTTACACACTTGGGGTTGGGGTTGATGAGAGAG
AAGCAAATGTGGGTTTCTATGAAAACGGTGAAGGAGTTTTTGAGTT
CACAGCAGCAGAAATGGTTGAGATTGAGAATGGAGATGGTATTAAG
GATACCAAACAACACTCGAGTGAGAAGCGTAGGAGAGTGGAGTTG
AAGGGCAAGTATGAGTCTCTGCGACTACTGGTTCCTGATCCCAGTAA
GAATGACAGAGCGTCGATCGTGGCCGACGCCATTCGCTACATCAAG
GAGCTGAAGAGGACTGTGGGTGAGCTCAAAGGTCTCGTAGAGAGA
AAGAGATGTGCGAGAGAGAGAGGTTCAAGAGGCCTAAGGTGGAAGAA
GATGACGTGGATCCTTCGCTGCGGAGCTCGTGGCTGCACCGCAAGT
CCAAGAACACCGAGGTGGATGTCCGAATCGTGGACGACGAGATCAC
GGTGAAGCTGGTGCAGCAGAAGCGGATCAACTGCTTGCTGTTTGTT
TCCAAGGTGCATGATGAATTGCAGCTCGATCTTCAACATGTTGCTGG
AGGCCTCATTGGTGATTATTATAGTTACCTTTTCAACTCCAAGATTTG
CGAGGGCTCGATTGTGTACGCGAGTGCAGTAGCGAACAAGCTGATT
GAAGTTGCGGACAAACACTATGCTGCACTCCCACCAACAACCTATTA
G

SmbHLH19 ATGCAGGAAAATGATGCATCCATTAAAGCTCTATTCAATGGCTTCACG
GGTTCCCTAGGTCAAACAACAAATCAGGATCAGCATTTCCCTCACTC
TCAGACTGATAGTTTTGGAGTCTCGGCGTCTGCGGCGGCGAGCCAG
CCGTCGGCGGCTGGACAACCTCGGCAGCGGGTGAGGGCCAGGCGA
GGGCAGGCCACCGACCCTCACAGTATTGCTGAAAGGTTGAGGAGGG
AGCGAATTGCGGAGAGAATGAAGGCTTTACAGGAGCTTGTACCTAA
TGCTAACAGGACGGACAAAGCCTCAATGTTGGATGAGATCATCGAC
TATGTCAAATTCCTACGGCTCCAAGTCAAAGTACTGAGCACGAGCAG
ATTAGGCGGAGCTGCAGCTGTCGCCCCACTACTTGGTGATGCATCCT
CTCAGGGGAAGAAGGGAGAGAAGGAAGGAATGGCGACGGCGGAG
CAGAAGGTGGCGAAGCTTCTGGAAGAAGACATGGGTTCGGCGATGC
AATATCTTCAGGGAAAGGGTCTTTGCCTGATGCCTATTTCTCTGGCCA
CCTCCATCTCCTCCTCCTCCACCCAAACCTCTACTTAA

SmbHLH20 ATGCTGCCCCGCTCCAACACGGCCCTCTGGATGGACGGAGAAGAAG
ACGAGGCCATCTCATGGCCACCGCCTCGGACCCCCGCCGACGCAAT
CAACAACGCCTCCTTCAAGTCCATGCTCGACCCCGATTGCTTCATCA
ACACGCCCAATCCATTCTATCACCCTGAATTCAGCACCCTTTTATTCA
ATCCCTTGGATTCCTCTTCCTCCTGCTCTCCCTCGCACCCCTTCCACC

TCGATCCCTCGCACCAGATGCCCGCATTTTTGCCGCCTAAATCTATGC
TTCCTTCTCTCTTCACCAACAATTTTGATAATGGCTTCGATTTGAGCT
GCGACCAGAGCTTCTTCCCAGCTCCGGACGCTTCTTCCGCGCCCATT
TTGATGGGTTTTAATGGATTTAATTCCCAATTTCAGTCCAGCTCTGAG
TTCCAGACCGCCTCCCGGATGCTTCCTTTTTCCGAAGATAACGCCGC
CGCAACTGCCACCGCCATTGGAGGTGCTTCGAATGCATTCAATTTCG
AGTGTTTCGATGCTAGCGGCGGATTATACCCCAACAGGTCGAAGCTG
CTGAGGCCGCTCGAAGTTCAGCCGTCAGTCGGAGCGCAGCCGACTC
TCTTTCAGAAGAGGGCGGCGCTGCGGCAGAGCTCGAGGCTGCAGA
ATTTGGGGACTATGAGCTCCAAGAGCGACGAGGGTTTGACGAGTGT
GGATGATACGGGGGGAATGGATATGGGGAGGAAGAGGAAGAGGAAT
TTTGAGGATGAAATGGAGGATATGGGAGCTGATATCTCCACTATGAAT
TACGATTCGGATGACCCCACCGAGAAATTCGGCGACGGAAATGGCG
ACAATTCCAACGCCAACAGCAGCGTGACGGTCGGCGACCAGAAGG
GGAAGAAGAAGGGGATGCCGGCCAAGAATTTGATGGCGGAACGCC
GCCGGAGGAAGAAGCTCAACGACAGACTTTACATGCTCAGATCTGT
TGTACCTAAAATAAGCAAGATGGATAGAGCTTCAATTCTTGGAGATG
CAATTGATTATTTGAAGGAACTTTTGCAACGCATAAATGATATTCACA
ATGAACTTGAAGCTACTCCAGCTGGATCTATGATGCATCCATCGACA
AGCTTCCATCCTCTGACGCCCACGCCTCCCACTCTTCCTTATCGCGTT
AAGGAAGAAATCTGTGCAAGCTCAGCACCCAGCCCCAAAAACCAA
CCTGCAAGGGTTGAAGTAAGGTTGAGAGAAGGGAGGGCTGTGAAC
ATACACATGTTCTGTGCACGTAGACCAGGTCTCTTGTTGTCGACTATG
AGGGCTTTGGACAACCTTGGTCTGGACATTCAGCAAGCTGTGATAA
GCTGCTTCAACGGCTTTGCTTTGGATGTGTTCCGAGCAGAGCAATTT
AGAGAAGGGCAGGATGTGTTGCCGGAGCAGATCAAAGCAGTGCTTC
TGGATTCTGCTGGGTTTCATGGTGTTATGTAG

SmbHLH21 ATGGACATCGGCTTCATGAAATCCGACCAAATTGAAATGATGCTGAT
GCAGATGGACAAGCTCCCAGATTTCAGCGCCGCCTTCGACGACGAC
GACAGCGACGACAATCCGAATATAACCGGGAACAACAACAACAACG
TCGATGCTTTCGAGAATTTCAACGTTCAATCGCCGCCGGCGTATGAC
GGCTTCCACGGCCAACAAGATCCGCCGGCGGCGAGCTCAATGGCGG
CGATGAGGGAGATGATATTCCGGATTGCGGCGATGCAGCCGGTCCAC
ATCGACCCGGAGTCGGTGAAGCGGCCGAAGAGGAAGAACGTGAGG
ATATCGACGGACCCGCAGAGCGTGGCGGCGCGCCACCGCCGGGAGA
GGATAAGCGAGCGGATCAGGATCCTGCAGCGGCTCGTCCCCGGCGG
GACCAAGATGGACACGGCGTCGATGCTCGACGAGGCCATCCACTAC
GTCAAGTTCTTGAAGAGCCAGGTGCAGTCGCTCGAGCGGGTCGCCG
CCGCTCGCCCGGAACCGCCCGGGGTCGGCTTCCCGGTGCCCATGTC
GAGCGGGAGCTATTTTCCGCCGCCGCCGCCCGCCGCCAAGGGGTAC
CACAATGTTCAGTATTGA

SmbHLH22 ATGGAAAATTTCGATGATGATTACACAAATTTTTGGGAAGCCAACAT
GTTCCTCCAAACCGAAGAGCTCGGAAGTTATTTCGACGAGGCTATAT
CGGTGTACTACGACTCGAGCTCGCCGGACGGCGTGCAGTTGTCACC

GGCGTCGAAGAACATAGTGTCGGAGAGGAATAGGAGGAGGAAGCT
TAATGAGAGGCTCTATGCTCTAAGAGCTGTGGTCCCAAACATTACAA
AGATGGACAAAGCATCAATAATTAGAGATGCGATTGAGTACATAAAA
TCGCTCCAAGATGAGGAGAGGAGAATTCTTGCTGAAATTTCGGATTT
GGAATTAAATGGAGATTTTTTTGAGATGGATCAAGAAGATGTTACAA
ACTTTCGTTCTAAGCCAAAAAGGACTAAAGTGGACAAGGCATCATC
GCCTATCGAGGTGCTCGAGTTTAGGGTATCGAATATGGGTGAGAATG
TAGTGGTGGTGAGCTTGGCCTGCAGTAAAAGAAGAGACACAATGAT
TAGGTTATGTGAGGCCTTTGAATCTTTGAAGCTCAAAATCATTACTTG
CAGTATCAATGCTTTCTCTGGTAGACTATTCAAGACTCTCTTTCTAGA
GGGTAATGATGAGGATAAAGATGTTCTAAGAGAAAAGATAGAGGCC
GCCATAGCTGCAGCTAGCTCTAAATAA

SmbHLH23 ATGGAGTTGTACAGAAATGATGAGCATGGTTTCTTGGAAGAGTTACT
AGAAATGGAGTCCTTCACAAATCAAGAAAGCTTGCCTAGTCTTGCTT
ATATCCCTTCCTACCATCACCGCCCGACTTCAGCTTCATCCAAACGCT
CCCTTATGATTTTCAAAATCCCGAACCCCACAAGACGGCGGCGGAA
GCGCCTCAACGACCGCCTCTCCATGCTCAGATCAGTTGTTCCTAGGA
TTAGCAAGATGGACAGAGCATCTATTTTGGGAGACACCATAGACTAC
ATGAGAGAACTGCTGGGAAGAATCAACAACTTGCAGGAAGAAACG
CATTTATTCAAGGATGTCAAACCCAACGAAATTTTACTCAGAAATTC
GCCCAAGTTTCAAGTGGAAAGAGGAAATCCGGATACAAGAATTGAG
ATTTGCTGCGGCGGGAAGCCGGGATTGTTGCTGTCAACGGTGACTA
CATTAGAAGCATTAGGCGTCGAGATTCAGCACTGTGTTATTAGCTGTT
TTAATGACTTTGCACTGCAAGCCTCTTGCTCAGAGGATTTGAAGAAG
ATAGCGATTTTGGATCCAGAAGACATAAAGCAGGCGCTTCTGAGAA
ATACAGGATATGGAGGGAAGTGCCTCTAG

SmbHLH24 ATGACCCTTTCCTCCTCCCGCCGCCGCCGCAGCAGGCAGCCTGCGCC
GGCCTCTTCGGCAGAAGGCCGTCAGTCGTTAGGGCAGGTGGTGCAC
CCCGGCGGCGGCGGCGCCCCCTTCGGCCTCCACGCCGAACTGCAGA
AGATGACGGCGCAGGAAATCATGGATGCCAAGGCCCTCGCCGCGTC
CAAGAGTCACAGCGAAGCTGAGAGAAGAAGAAGAGAGAGAATCAA
CAACCATCTTGCTAAGCTCCGAAGCTTGCTCCCTAGCACCACCAAAA
CAGACAAAGCCTCACTGCTGGCTGAAGTGATCCAGCACGTGAAGGA
ACTGAAGCGGCAGACCTCCCTTATAGCGGAGAGCAGCCCCGTCCCG
ACGGAAAGCGACGAGCTAACCGTGGATAAGGAGGCGGACGAGGAG
GGCAGAGCGGTGATCAAGGCGTCCATCTGCTGCGAGGACCGCTCTG
ATCTCTTGCCGGATCTCATCAACACGTTGAAAGCCCTCCGCCTTCGG
ACGCTCAAAGCTGAGATCACAACACTTGGTGGGAGAGTCAAGAATG
TGTTGTTCATAACCGGAGATGATGAATCCACCACTTGTGATCACCAC
TCCATCCAAGATGCGCTTAAAGCAGTTATGGATAAAACTGCTGAAGA
CTCCGCTTCGGGGAGTGTCAAGAGGCAAAGAACCAATATCAATATCC
TTCAACACAGGTCGCTTTGA

SmbHLH25 ATGGAGCTGTACTCAAAGGAACACCAAGGTTTGTTGGAAGAGTTAC
TAGGCCTAAGAGAGATGGAGCAGTACAACACACACAATCAAGAATT

CAACACAAGTTGGAACATTCTGGAAGATGTTTCCATCCCAACAAGC
ACTTGCTTCCAAGATTTCTATCTCCCCATCGACCAAACCCTACACTAC
AGCGCCCCGTTTGGCGACGGCCTCTCGCCTCCCTCAGATTCATCAAA
CACCAGAATCAACTCCCAAACACTCCCTTACAGTACTGTCGCCCCGC
AACCACAAGACTACTACTGCAACGCCTTCTCCATTCTTCACGACGAA
GACGCCGCCGTTTTCGGAAACGGGTTCCGCGATTTGGATATCGGGGC
CCCGCTCAAGGCCGAACCCGGCCTGCCCGCTTTCGACATGGGTTTTA
GCCATCAAAGTGAGAGAAAGAGCAAGATGAAGAAGCTCAACGGCC
AGCCCTCCAAGAACCTCATGGCGGAGCGCCGCCGCCGGAAGCGCCT
CAACGACCGCCTCTCCATGCTTAGATCAGTCGTTCCCAAGATAAGCA
AGATGGACAGAACATCAATACTCGGCGACACCATAGATTACATGAAA
GAGCTGCTTCAGAGCATAAATAATTTGCAAGAAGAAATGAATTTGGG
GGGAAATGAATTGGAGTTGTTGAGCATATTCAAGAACGCCAAGCCT
GAAGAAATGTTGATCAGAAATTCACCAAAGTTTGAAGTGGAGAGGA
GGGAGTCAGAGACAAGAATAGGGATATGTTGCGGTGGAAAGCCAGG
GTTGTTGCTATCAACAGTGACAACTCTAGAGGCATTGGGCATCGATA
TTCAGCACTGTGTTATTAGCTGCTTCAACGATTTCGCAATGCAAGCTT
CTTGCTCTGAGGATCTGAAGCAGAGGGCAGTGTTGGATGCAGAAGA
CATAAAGCAGGCACTGTTTAGAAACGCAGGATATGGAGGAAAATGT
CTATGA

SmbHLH26 ATGGATAAAGATTACTTTATGAATGCTGGAATTCCACCACCTCAACCA
CTCCACTTTGAAACCTCAATGCCAATTCCATGGAGCTCTGATCAATC
CTTCTTGAACCCTAATTCCGCCATGGATCATCACCAATTTGACTCATC
TTGGACCTCAATGGCCACCACCACCACCACCACCACCACCACCAAC
GCCGACGGTTTCGCCCTCCGCCAGCTCATCGGGAAGCTGGGCGGCG
CCGCCGACCGCCTTTCACCCACGCTGAACTTGCCGCTTCTCGATCAG
ATTAACCTCCCCAATCTGGCCACCACCTCCACTCCGCCGCCGCTGCC
GTCGCTCGCCGCCGATCCGGGCTTCGCGGAGCGGGCCGCAAAGTTC
TCCTGCTTCGGCAGCCGCAGCTTCAACGGGAGAACGTCGACGCAGC
ACCCGCAGCCGAGCCAGGCCGAGCCGATGCGTGGATCGAACCCGGA
GCCGAAGCTGCCGCGCGTCGCGAGCAGCCCGTCGCTGAACAGATCG
CCGCCGCCGAGCCGGATTAAGCCGGTCCTGGAAATGCGGCCCGCCG
AGAGGAAATTCGGCGCCAGCTCCAACGAGGAATCCTCCGTCTCCGA
GCAGATTCCGAGCGGCGAAACCGGCTCGAAGAATGAGATGAATTCT
CGGAAGAGAAAGGCGGCTTCCAGAGGCAAAAACAAGGACGACAA
ATCGAATTCAGCTAAGGGAGGCGAAGGCGACGACGACGGAAACTC
GAAGCGGTCGAAGCAAACGGAGAATTCGGAAAATGAGGAAGAATG
TAATAATAAGGCGGACGAAAATAAGGCGAATCAAAAGCCGCCGGAG
CCACCGAAGGATTACATTCATGTCCGAGCCAGAAGGGGCCAAGCCA
CTGACAGCCATAGTTTAGCAGAAAGGGTGAGGAGAGAGAAAATTAG
TGAGAGAATGAAGCTTCTCCAAGATCTCGTACCAGGTTGCAATAAGG
TGACTGGAAAAGCACTGATGCTGGACGAAATCATAAATTACGTGCA
GTCATTGCAGCGACAGGTTGAGTTCTTGTCAATGAAGTTGGCATCAG
TGAATCCGGAACTTGATTTCAACATGGAAAGCCTTCTCTCCAAAGAC

ATGTATCAGAGCACGAGTCTACCACAACAACAAATGTACCCTTTGGA
TTCCTCTGCACCAGCATTCTACCATCACCAGAATGTACAACACCAAC
AGCTTCATGGTACAACCATCTCAAGCGTGGACCCTCTCCCCCTAGAT
GCTAGCTTAGGGATTCAGATGCCTTCTGTAGATGGATTCGCCGAAAA
TTTGCCCCAGTTTCCAGCATTCACTGAAGATGATCTGCACAGCATTG
TCCAAATGGGATTTATCCAGAATTCTGTTCACTTCCCAGTTCAAAATC
AAACCTCAAACATGAAAGTTGAGCTATGA

SmbHLH27 ATGGAGCCCGAACTCGGTGCAGCTCAACCGCAGTTTAGATCCGGCG
AAATGGGGTGCGCGTTTGACGAAATTCACGGACTCATCTCCGCTCCA
CCGGTCACCGGCGGCAGCTCGTTCACGGCGCTGCTCGAGCTTCCCC
CGCCGCAAGCGGTGGAGCTCCTGGTTACGGAAGATTTTCCGGCTAA
ACATCAGCCGCCGCCGCCGATTTTCCCTACTGATATCGGTCTCATCCA
TCGCGCGTCAAAGTTCTCCGTCTTCGCTTCAGCTGATAATTCGCTCG
AGAGCAATACGATTCTTTCAGTCTCAAACTCGATCAAGGTTGACTCG
GTGAAGCAGGAGCAGCTGGACGCGGATTCCCACCGAAATTCTTCTT
CTCCGGCGGGTTCGGATCAGTGTCTCAAGTCCGGCAAGCGAAAGGA
GAGGGAGAAAAAGGTGAAAGAGTCGAATAAAAAGAGCAAAAATAT
GGCCGCAAATGAACCTTCTGACAACGGCGGCGGCGAGGAGCTGCCT
TACGTTCACGTCAGAGCTCGCCGCGGCCAAGCCACTGACAGCCACA
GCTTAGCAGAAAGAGCAAGGAGAGAGAAGATTAATGCCAGAATGA
AGCTGTTACAGGAGCTAGTCCCCGGATGCAACAAGATTTCAGGAAC
TGCAATGGTGTTGGATGAGATAATTAATCATGTCCAAGCACTTCAAC
GCCAAGTGGAGTTTTTATCCATGAGGCTTGCTGCTGTTAGCCCAGGA
ATTGATCTTGACCTCGATTCCCTATTCGCTGTGAATGGATCTTCAAAC
GACGATTCCAGCTACACTGGTATGTTCGCTCCACCCATCTGCACTGA
GGGACAGCTTGATGGGACCAGACAATTACAGCTCCAACAGCTGTGG
CATTGTGATGAGCTTCATCAGTCTGTCTGGGCCAGAGAAGCTGACAC
CTCTAATTTCATTGCTCCTGCAAATTCACTAATGAGTTACGACTCCTC
CTCCGCAAATTCAGAGCTGAAAATGGAGCTGTGA

SmbHLH28 ATGCAACCGCCGCGCGGCAGCGGAAGCGCCGAGCTGACTCGGAGC
GACGGCGGTGGCCTCGCTAGATACCGACCGCCTCCGCCGCCCAATG
CCTCCGCCGCCGACGTCGATCTCGAGCTGCTGGAGTCCGCCGGCGG
AGGCGGGTTTAGCAATTTCCTGAGGATGAACAGCTCGCCGGCGGAG
TTTTTGTCTCTGCTTAATAGCTCGGAAGGGTTTTTTTCGAATTTAGGT
ATTCCGGCGGATTACGAGCTCGTGCCGGCCACTTCAGCTAAGCGCGC
ACGCGAGGCGGAGGATTTGGATAGGATTTCGCCGAAGAAGTCTCCG
TCGTCGTCTTCTTCTACTCATTTGAAAGGAGAGAAGCGTAGGCAGTT
GCAAGGCTCGGGTGGGTCATTGGATCTTGACATGGAGAATCTTTTGG
AGGACTCAGTTATGTGTAGGGTACGAGCAAAACGTGGTTGTGCTACT
CATCCTCGCAGCATTGCTGAAAGGGAACGAAGAACGCGGATCAGTG
ACAGGATAAGGAAGCTGCAAGAACTTGTTCCTAATATGGATAAGCAA
ACGAATACCGCAGATATGTTAGAAGAAGCTGTAGCTTATGTCAAGCA
TCTGCAGAAAGAGATCCAGCTAAGTGGATATCATAATGATCAGCTTG
GAGAAGATCTGTATGGTTGA

SmbHLH29 ATGGATCACGACTTAATCCTCTCCGCTTCGGCTTCATCGTCTTCCTCC
ACCACCACCTCGACGGCCGCAGTGTCTCTGGTATCCTCCGTTTCCCA
GCCGGCCGTCCTGCAGAAGAAGCTCCACCACATCCTCCAAACGCAG
GCGGAGTGGTGGGCCTACGCCATTCTGTGGCAGACCTCGAAGGATG
AATCGGGTCGCATCGTCCTCACCTGGGCCGACGGCCATTTCCAAGGC
ACGAAGCAGAAAAACCCCCCCTCGGGGTCCCTCCAGCCGGAGCGC
AAGAAGGTGATGCGAGGAATCCAGGCTCTCATCGGCGAGGGCGTGG
ACCCCCTCGAGGGCGAGGTCACCGACGCCGAGTGGTTCTACGTCAT
GTCCCTCGCCCAGTCCATCTCCCTCGGCGACGGCGTTGTCGGCAAG
GCCTTCAACTCCGGATCCCTCGTCTGGCTCACCGGCGCCAATCAGCT
CAGATTCTACAACTGTCACCGCGCTAAAGAGGCGCAGATCCACGGG
ATGCAGACCATGGTGTGCATCCCCACATTCGACGGCGTGCTGGAGCT
CGGATCCGACCTCATCGTCGCCGAGAATTGGAATTTAGTGCAGCAGG
CCAAGTCTCTCTTCGATCCCCAACCCACCGCCACCTTCCAAGACGCC
CCCGCGCCCAAGCTCCACCTCGACTTCCTCGACCACTCCGACTCCG
ACTTGTTCGCCGAGCCCATGGAGACCAAGAGAGCCCCCAAGAAGA
GGGGCCGGAAGCCCAACCTCGGCCGCGACGCGCCGCTCAACCACG
TGGAGGCCGAGAGGCAGCGACGGGAGAAGCTCAACCACCGCTTCT
ACGCGCTGCGGTCGGTGGTCCCCAACGTGTCGAGGATGGACAAGGC
CTCCCTGCTGTCGGACGCCGTGTCCTATATCAAGGAGCTGAGATCTA
AAGTGGAGGAGCTGGAGCAGCAGCAGCCACACGCGAAGCCGGTGA
AGACGGAGACCGGCGACAACCACAGCACCACCACCACCGCCACCG
TGGACCAGCTGGTGCTCTCGTCGGCGTCGCCCGGTGGAGGTGGAAG
TGAAGATCGTGGGCGGGGACGGGATGATCAGGGTGCAGTCGGACAA
GTCGAATTACCCGGCGGCGCGGCTGATGAGCGCCATCCGGGAGCTG
GAGCTGCCGCTGCACCACGCGAGCATGTCGTGCGTGAACGAGCTGA
TGCTGCAGGACGTGGTGATCAAGGTCCCCGAGGGATTGAGATGCGA
GCATGCTTTGAAAAACGCCATTCTTACAAGATTAATAGACCACTGAT
GAGGCATCCATCCATTATCATCAATTATATTAATATTATGGGATTATTAG
ATTAG

SmbHLH30 ATGTTTCCAATCTCAAGTGATGGGGTAATTGAGGACCCTTGGATATTT
TTAGAGCAAGATGGATTTCTGCTTGCGGATATTCCCATTTTAGAGGGC
AACGAACATCCTCAAAATAGTGTGAAAAAGAGGCCGAGAAAAGCC
CCAACCTCCGCCGGAGTTGAAGAAAACAGCAGCGACTCCAAGAAG
ACTGCTCACAGATTTACTGAGAAGCAGAGAAGGCAAGAAATGACAA
CTCTTTATGCTTCTCTCAGATCCCTTCTCCCTCTTGAATACATCAAGG
GAAAGCGCGCTGTATCCGATCATATGGACCAGGCGGCCAATTACATT
AACGATATGAAGAAGAAAATCGAAGAAATGAAACGAAGGCGGGAG
AGCTTGAGGAATGTAGCAGGAACTGCTGCTGAAGCTCAGAATCAAA
CCTCCAATGCTACCGATTACTGTGTTAAGATAAATGTATTCACAGATG
GGGTTGAGATCTTGATTAGTAGTAGCCTCAATAAAGATTGTTTCCCTC
TTTCTATGGTGCTTTCCGATTTGCTTAATAGACAGCTCCATGTAATCA
ACTGCGTTTCTACAAGAGGCCAACGATGTTACCTCCACAAAATTCAC
ATTCAGCTTAATAATTCCACAAGCATTGATGTAGCCGAGTTACAAGA

| | |
|---|---|
| | GAGATTGGTGGATTTGCTTCAACTTGCTTGA |
| SmbHLH31 | ATGGCAAGTGGAAACCAAAACCAGCAAGAAATGCCTCAGAATCTAA<br>TGATGCAGCTTGCTCTTGCTGTTAGAAGCATTCAGTGGAGCTATGCA<br>ATCTTCTGGTCCGTTTCATCCAGACAACCAGGGATGTTGGAATGGTG<br>CGAAGGTTACTACAACGGTGATATCAAAACACGAAAGACAGTTCAG<br>GCTGCTGAGGTGAACATGGATCAGTTGGGGCTACAGAGAAGTGATC<br>AATTGAGAGAACTTTATGAGTCTTTGTCTCTAGGCGAAACCAAACCT<br>CAAGCTAAGAGGCCTACAGCTGCATTATCCCCCGAAGATCTCACTGA<br>TGCAGAGTGGTATTTCTTGGTTTGCATGTCTTTCCTGTTCAACACCAC<br>CGATCAAGGGTTGCCTGCAAGAACATTAGCTACGAATCAAATGATAT<br>GGCTCTGCAATGCTCACCGGGCTGACACCAAATTCTTCTCTCGTTCT<br>TTGCTTGCGAAGACTATTGTGTGCTTTCCACATTTAGGAGGCGTGGT<br>TGAGCTCGGAACGACTGAACTAGTTCCAGAGGATCCAAATCTTATTC<br>GGCATATAAGTTCATTCCTGGAGAGTCCTTCTGTGACTGTTCCTGTGA<br>TTCCTAACCTTGTCTTCGACAACAGTTGTAACCATAATGGTTTCAATC<br>TTGAACGGCTCGACCATGCTAATCTTCCCGAAGAGGGTCTTGATCGG<br>CTCATGGATGATCCATACATGGAGATCTGTACTCCAAATAACTCAGAT<br>GATTTTGCAGACAATCTGCTGAGAGAGGAGATGAATTTGGTGGAAG<br>GCGTAGACGCTGAGGCTTCTGAAATACAAAGCTGGCCAGTAATGGA<br>AGATGCAGTCAGTAATTGCTTAAATAATTCTGTGAATTCCAGTGACTG<br>TGTGTCTCAAAGCGAGGGAGAACCCGAGACGATAATCCCACATTCG<br>GATGGGAAGAAGGAGAATAAAAGCTGTATGGAGGAATGCAATCAGA<br>AGACAGCCTCTGAATTTCAAGGAAATGATGTTCGTTACCAGAGTGTT<br>ATTTCCAACCTCCTGAAGAGTTCTCACCAGTTGATTCTTGGTCCTTAC<br>ATCAGAAACGGGAGTAGAGAATCGAGCTTCGTTTTCTGGAGGAAGC<br>ATGGGGCTCCGGGACAACAAAGAGGAACCCCACAGAAGCTACTCA<br>AGAAAGTACTCTTTGAAGTGGCTAGAATGCATCAAAGTTGCAGGGC<br>TGACTCTGAGAGGCAGCACAACAGCTGTTCCAAACGGGAAGCTGAT<br>GAAATCGATAGAAACCATGTCTTGTCTGAACGCAAGCGCAGAGAGA<br>AAATAAACGACAGATTCATGATTCTTGGATCCCTAGTCCCATCTGGTG<br>GAAAGGTTGACAAAGTATCGGTGCTTGATCACACGATAGAGTACTTG<br>AGAGAGCTCGAGAGAAGAGTTGAGGAGCTGGAGTCCTACAAGAAA<br>GCAATGGAGCGAGAGTCAACAACACACAGCAAATCTCAGGATGCCA<br>TTGAGAGGACCTCTGATAACTACGGTAATGATAACAATCCAAAGAAG<br>CCAGCAACGAACAAGAGAAAGGCAGCCTGTCATAATCATAAGGACA<br>GAACAGGAGCTGAAAACATCAAAGCTCGGTTGAGAGACTCCTCCTC<br>CCCTGCTGATAACATAACCGTCACTGTCTCAGACAAGGTCGCCTTGA<br>TTGAGACTATCCAATCATCCACCAACAACGGGACCCTCTCTATCATTG<br>TGAATGCCAAGTGCAAGGGACTGAAATCTCCATCAGCAGTAGTGAT<br>CAGACAAGCTCTTCAGAAAGTTATCAGGAAGAGTTGA |
| SmbHLH32 | ATGGTGGACTGCATGCTGAATAATCCACCATCACCACCTCCACCTTC<br>GAGTTCATTAGCAGATACAATGCAGCTTGCAATTGATGAGGCGAAAA<br>CCTCGGTGGAAGGCAAGGGCAAGAACAAGAGGAAGAGGGACAAA<br>ACTAACAAGTCTATTGAAGAAGTCGAGAAGCAGAGAATGACTCATA |

TCGCAGTGGAGAGGAACCGGAGGAAACAGATGAACGAGCATCTTC
AAATCCTACGCTCTCTCATGCCGACCTACTATGTCCACAGGGGAGAT
CAGGCTTCCATAGTTGGTGCAGCAATCGAGTTTGTGAGAGAGCTGC
AGCAACTTCTCCAATGCCTTGAATCAGTGAAGAGGCATCAACATCAT
CAACCTCAGATGATCTTGCCTCCAACACTTGATCAGACGGCAGAGA
GCAAGTCTTACTTGGCAGATGTTGAAGTGAAGCTCTCAGGGTTGTTT
GGCTTGATCAAGATTCTCTCGAGAAGGCGGCGCGGCCAGCTCATCA
AGTATATTGCTGCAGTTGAGCATCTGCACCTTCATATTCTGCAAACTG
ATATTACTACCATCCAACAAGATGTTGTCTGCTCTTTCACTGTCAAGA
TGAGTAGAGAAGCAATGCTCACAGCTGAAGATATGGCAAACTCAAT
TCAGCAGATATTGAATTCTCTTCATGCCAGCAGCAGCATGTCATGA

SmbHLH33 ATGGGTTCTGAGGGAAGTGGTGGTAGAGTGTTCCAACACAGAGGTG
GTGGTGGTGGTAGGGCTCTGAATTCAGGTATGGGGGGCACAAATTC
AGTTTCTGACAAAGTTTCAGGAGTGACAATGTGCTCAGAATCCATGT
TCTATGGCTCTAATTGGGATCCAATTATGTCATTGAGTCAAAGTGGGA
ATTTTGGGAATTCTAACATCGTTTCTCAGAGTGAGTTTGCTAATCCAC
TCATGTTGGAGAATCAGACAATGGGTAGCAGCTCTTTGCCTCAGTTC
CCCTCTGATTCTGGTCTTGTTGATTTGGTGCCAAGAATCCCCAGCTTT
GGGAGTGGCAGCTTCTCTGAGATGGTCACTTCCTTCGGCCACAACTC
CGAGTCGCCTAGTCTTAAGAAGGGAGTTTCGCAGGATCATTGCCTGA
ATTCGGATAATGGGATGTTGGGAGCTTCACCTTATGGGAAGAGGAAG
AGAGAGGTGCAGCAAGGAGCTTCACCAAAGAGTGCTGAAGAAGAA
CAGCACAAGGATCCCTCTGGGGATACATCTAGAGAAGATGATGATAA
GAAACAGAAATTCGAGAAAGGTATTGCTCGCGGCAAGCAAGCTGCG
AAGCAAGCCAAGGATGATCAGAGTGGTGCAGAGCCATCTAAAGATG
ATTACATTCATGTGAGAGCCAAAAGGGGCCAGGCAACAAACAGTCA
CAGCCTTGCAGAAAGGGTGAGAAGAGAGAGAATTAGTGAGAGGAT
GAGATTGCTTCAGGAACTGGTTCCTGGCTGCAATAAGATTACTGGGA
AAGCAATGATGCTTGATGAGATTATCAACTATGTGCAATCACTCCAAC
AGCAGGTTGAGTTTTTATCGATGAAGCTTGCAACCGTAAATCCAGAG
CTCAACATGGATATAGACAGGATTTTGTCCAAAGATATTCTTCACTTG
CGTGGCGGCAACACCACCCCACTCGGAGTTACTCCAGGGCTGAGTT
CGTCCCTCCCGTTCCCTCCATATCCCCAAGGAGTCTTCAACAGCGCC
CCAGGCTCAGGTGCAGCATTTCATTCCTTACCCCAGCATATATGGAAC
ACTGAGCTCCAAGGTCTTCTCCAAATGGGGTTTGATCCCGATCCCTC
CGGTGGCATGGGGCCAAATGGGTCGTCGAAAATGGAGCTATAG

SmbHLH34 ATGAAAAGAGACGGTAGACAACAAGATGAAATTCCAATTAGTGTTC
CAGATTTCTCGATATGGGACATCGGTTTTGATCAAATTTTATTTCACA
ATGATCCAACCAATCTCGAAGTAAAAAACCATAAAGGAAATAAAATT
GCAGGTAAATTACAAGAGATCGAAGGCTCAAGAATCAGTGAGCGGT
TGGATAAGAAAATAGTGCACAGAGAAATTGAGAGGCAAAGGAGAA
AAGAAATGGCCGACCTTCACGCCTCTCTCCGTTCAGTCCTCCCTTCC
AAGTGTATCAAGGGCACGCGGTCGGCATCAGATCAAATTCATGAGG
CTACAAAGTATATAAGGTATATGCAGAATAAAGTTAGACAGTTGGAA

ATGAAGAGGGATAGCATAAAGAAATCGCCAGAATATGAAAAACAGG
GTTTGTTGAGAGAGAGAGGGAGTTCAAGCAAGGCTTTGCCTATTAC
AGTAAGGGTTCAGCGATGCACGGCCGGCGTCGAGGTGTTGATCTTG
GCGGAAGGGCGAAGCCTTCACCTATCGAGAATCCTGGAGCTGCTGC
TCGATGAAGGCCTCAATGTTGTCCACTGTAATTGCACCAAATTTGAT
GGAAGGTTGCATTGCACCATCCAATCTGAGGATGTAACTAGTCCGAG
TATCGATCTCCAGATGCTGCAAGTAAAGCTGACGAGTAATATTGGCA
AATTTAGTGATCAAGGGTGA

SmbHLH35 ATGATCTCCGAGAGAAAGCGCCCCGAGAAGCTCAACGACAGCTTCC
AGATACTCAGATCCTTCCTCCCTCCTGGAACCAAGAAGGATAAGGCG
TCTGTGCTGAGCAACACCACCGAGTATCTGAGCTCGTTGAAGTCAC
AGGTGGCAGAGTTGGCCAAGCGGAATCTGCTTCTTGAATCGCATATT
AACTCGATACAGAAATCAGAAAGTGACACAGACGAAGCGGGGAGT
TCTTGGTCCGGTGGCGAAATGATTAGTGTGGAGATCGCGCCAGTTTC
ATCGTCGTCGTCGTCGTCGGAGGAAGCAAGATTCTTGGATTTGGGAT
GTGAGTTTGAGATCGGTGGAATCGAATACGAGGATGTTGGGATCAAT
TGCAATTCAATACTTAAAACATATATGGAGATGGGCCCCACGAGAAA
ATAA

SmbHLH36 ATGGCTCCGGCGAGGTTGGCTGCTGCTGCCTGTGAAGTTGGCGAGG
CCAATGCTATTTGGCACCGTCACCGTTGGGGCTGGGTCCAACGGTTG
CGGAAGCCCCACCGTCTCTGTATCTCCCTCTCTCCATCGCTCTCTCTA
ACACCGTCAAAAGACAATGAGAAAGCAGAGGAGCTAACTTTTGGA
GGGAGCACTCTAGGACATAGCAATAGTGAAAACTGCATCCAATCGTT
CTTGCTACTCTTTCTAGACGAACTGCCGAAGCAAGACTACATCCATG
TTCGAGCAAGACGAGGTCAAGCTACCGATAGTCATAGTTTAGCAGA
AAGAGCTAGGAGAGAAAAGATTAGTGAGAGGATGAAAATTCTTCAG
GATTTGGTTCCTGGTTGTAATAAGGTGAGTTGTCGACTTTGTTTGGA
CATTTTTGTCGCCTCTTGA

SmbHLH37 ATGGGAAAGAAAGTATGGTGGAATGAAGAAGAGAAGGTTGTTGTGG
AAAGTGTGTTGGGCAGTGAAGCAACTGAATATTTTGACTGGGCCGC
TTCAAATAATGTGTTGTCTGAGTTCTCTACTTCTAGTGGGGATCTTGG
AGTACAGGAGGCACTGTGCAAGATTGTTGAAGGATCTGATTTGACAT
ATGCCATATATTGGCATGTTTCGAAATCAAAATCTGGTAGATCAGCAT
TGATATGGGGTGATGGGCATTATCAAGAATCTAAAGAAAGTGTGCAT
GATAGTTGTGGCAGTTACAATGATCTGAAGCGGGTGGAAGGAGATA
GAAGAAAATGGGTGCTTCAAAAGCTTCACGCTTGTTTTGGGGGATT
AGAAGATGATAATGTTGCCGCAAAGTTGGATCAAGTTTCAAATGTGG
AGATGTTATATCTCACATCGATGTTTTTTGTGTTCCCTTTCGACAAGC
CTTCTATTCCTTCCCAGTCATTCAATTCTGATAGATGTATCTGGGTATC
GAATTTGGACAGTTGTCTTGAGCGGTATCACTCTAGAGCATATTTAGC
CAAGTTAGCACAGTTTGAGACAGTGGCATTTGTTCCAACAAAATCA
GGGGTAGTTGAAATTGGGTCGAGAAAGTCTATACCTGAAAACAAGA
GCATCATTAAGGCAGCCAAGTCTATAGTGGTGGCCTTCAACTCAACA
CAGGTAAAAGCTGTCCCAAAAATTTTTGGGCAAGATCTTAGTATGGG

| | |
|---|---|
| | GGGTTCACGATCAGGTACTATAAATATTAGCTTTTCGCCGAAAGTGG<br>AAGATGATTCTGGGTTTTCTGCAGAGTCTTATGATTTACAAGCAATAA<br>GTAGTAACCATGGTTATGCAAATTCATCAAATGGGCATTGTGGTGATG<br>ATGGTGATACTAAAGTATTTTCACACCATGTAGTAGCTGCAGGACTG<br>GACTCACAATCAATTATTTCTGGGATGGAGCTAGACGATCAAAGGCC<br>TAGAAAAAGGGGGCGGAAGCCTGCAAATGGGAGGGAAGAGCCGTT<br>GAATCATGTGGAGGCAGAGAGACAGAGGCGAGAAAAACTTAACCA<br>GCGTTTTTATGCGTTAAGAGCTGTAGTTCCTAATATCTCAAAGATGGA<br>CAAAGCATCTCTTCTTGGTGATTCAATTGCTTATATCACAGATCTTCA<br>GGCAAAGATCAGGGTTTTGGAAGCAGAGAAAGGGATAGGGAATACT<br>AATCAACAACTGAATCCTATTCCAGATTTTGAGTTTCATGAAAGGCT<br>TGAAGATGCTGTTCTACGTGTAAGTTACACTCTGGAGACTCACCCTG<br>TATCCAAAGTTGTAAAGGCGTTGAGAGAGCAGCAAGTGATGGCACA<br>AGAATCTTCTATCTCTATAGATGACAGTGGTGAAGTTGTTCATACATT<br>CTCCATTCAAACAGAGAGCGGTGACGCAGAACAATTGAAAGATAAG<br>TTAACTGCTGCTCTCTTGAAATGA |
| SmbHLH38 | ATGGATAATATTGATTGGGGCAATGAGCTTGAAGAAAGTTTTATAATG<br>TCCAGCTCAATCAGCCTTCCCTACTACAACAACAACAAGATTGAGAC<br>GATTTCGTCGAAATTAGATACATACAGTCCAGTTCATCAGCAGATGC<br>AGAAAACGGAGATATTATCCAATATTTTGCCTCAGCAATGGAATTCA<br>AGATCAGGAGCTGGCAGCGGGTGGGGAGTCGCTATCAATGCAGGAG<br>CGACGACGTCGGGCTCATTCATTGAATGCAGTAGGCCTTCTGCATTG<br>AACACTACTCCTCTCAAAAATAGCATCATTGATGATACGAACGTTCTC<br>GAACAACATCCTCCAATGGCGGCCAACGCCGCCGCCTCGTTCGAGT<br>CCCTCGACTGCTTGCTCTCCGCCTCCACCACCGACACCACCACGTCA<br>GCCGATCAAGACGACGGTATTTCCCTCATTTTCTCCGACTGCCAAAA<br>CCTGTGGAATTTCGCGAAATCGGGAAAAACGACCATTACTCCAGTTT<br>CGCGAAATTCCGAAGAAATCGTCAATAATCGGAGCAAGAGAAAGAG<br>AGAGGCGGCGACGAGCGAGCCGATCGGATTAACGGAGCTGGATTTC<br>CAGTCGAATTACTCCGATCACGGCAACGGTAGCGGCGGTTTCCAGAT<br>CATATCGGAAAATCAGCAAAAAAGTACCAAAAAGGCGAGATTAGAT<br>CAGAAACAGCCCCTTCCTGTGATTAATTCGTCGTCTTCCAACATCAA<br>TTTCCAGCAGCCTAGCTCGTCGGCGTCCTCCGGCGACGAGCAGGAC<br>GCGGAGGCGATCGCGCAGATGAAGGAGATCATCTACCGCGCGGCGG<br>CGTTCAGGCCGGTGAACTTCGGCGCGGAGGCGATGGAGAAGCCGA<br>AGCGGAAGAACGTGCGGGTGTCAAGCGATCCGCAGACGGTGGCGG<br>CGCGGCAGAGGAGAGAGAGGATAAGTGAGAGAATTAGGGTTTTGCA<br>GAAGCTGGTCCCCGGCGGCAGCAAAATGGACACGGCGTCGATGCTG<br>GATGAAGCGGCGAATTATCTCAAATTCCTCAGATCACAGGTCAAAGC<br>TCTCGAGGCGTTAGGGCAGAAGAGCGTGATTGATCCAACGGTTCATA<br>ATTTTCCGGCAAATACGAATCTCGCATTCTCCTCGCCGTTTCTTAGCT<br>ACTCATTTGCCATGCAGCAGCAGCCTCAGTTCTCCATCCATAATCCCA<br>ATCAATTCAAAAGTTGA |
| SmbHLH39 | ATGAATCAATGTGTGCCGAGTTGGGATCTCGACGATAATAGTTCCAA |

GATCAAGTACCAATCCAATTCACCTCAAGACGTCCCCTCTGGCGGAG
CTAACATGGGAGAATGGTCAGCTCGCCATGCATGGCCTGGCGTCCCG
CGCGTGGTGAACAAACCGAGCGCGTCGTCGCCGCCCTCGACGTGGG
ACAAGCCACGCGCCAGCGGCACGCTCGAGTCCATCGTCAACCAAGC
CACCTCCCTCGGCGAGAAGGAGTCCGAGCTCGGCGGCCACCGCGCG
AAGGCGAACCCCGCCGCCGTCGCCATGGATGCGCTCGTGCCGTGCG
GCGGCGGGGGGCACGAGCAGCAGCATCCGGCGGCGGCGCAGGCGC
ACGTGCGGGGGGCGAGCAGGGCCGGGTGCTCCACGCGCGTGAGCA
AGTGCAGCGGCGCGCGCGTGGGGAGCCGGAGCGTGGGCGAGAGCG
CCACGTGCGGCGGCGGGAGCGACAGCCGTCAGACGATATCGATCGA
TACATGGGGGGAGAGGGAATTCGGTGGGCCCGGGTTCACGTCGACG
GCGTCGCTAATCTCGCCGGAAAACACTATCTCCGGCCAAGACTACAC
GAAGACCTCCGCCGACGACCGCGACTCCGTTACTCTTCACAGCGGG
TCTCAGGCCTCTCATAGAAAAGGAGATGAGGAGGGCAGAAGAAGA
GACAACGGAAAATCCTCAGTTTGTACAAAAAGAAGTAGGGCGGCCG
CAATTCATAACCAATCAGAACGTAAACGAAGAGACAAAATCAACCA
GAGAATGAAGACATTGCAAAAATTGGTTCCAAATTCCAGTAAGAGC
GACAAAGCATCGATGTTGGATGAGGTGATAGAATACTTGAAGCAATT
GCAAGCTCAAGTGCAAATGATGAGTAGGATGAACATGCCATCGATG
ATGTTGCCACTAGCCATGCAGCAACAGCAGTTTCAGATGTCGATGAT
GGGATGCATGGGCATGGGCATGGGGATGGGGATGGGCGTCATGGAC
GTCCCCCGCCCGCCCTTCATGCCCGTGCCTGCCGCCGCCGCCGCCTG
GGACAACGGAGGGGACTGTTTGCCCCCGCCGCCGCCCCCCGTCGCC
ATGCCCGACCCCTTGGCGGCACTTCTTGCATGCCAGTCTCAGCCGAT
GACGATGGATGCTTATAGCAGGGTGGCAGCTATGTACCAGCAATTGC
AACAGCAGCAGCAATTGCCTGCTTCAAAAAATTAA

SmbHLH40 ATGGCAGCTCTCACTGTTCAACACGATCCATTTATTTACTCCTGCAAA
GACTACACTTACAATATGGAAGAAGAGAAACCAAACCCTCGAAATG
TTGAAATTTATGGAGAATATTGGGAGTCTCCGCCTTCTTCAGCCGCTC
AGGCCGCCATCTCTTCCCCGGAGACTTGCGGCTCTCCCACGGGGAA
GTGCCGCCGGAAGAGACGGCGCGTGAAGCCCACCAAGAATAAGGA
GGAAATGGAGAACCAAAGGATGACTCATATCGCCGTCGAGAGGAAC
CGCCGGAGACAGATGAACGACTACCTCGCCGTTCTCCGCTCCATAAT
GCCCCCTTCCTATACGCAAAGGGGTGATCAGGCATCAATAGTTGGGG
GCGCAATAAATTTTGTGAAGGAACTCGAACAACTCCAACAATTTCTT
GAAGCAAATAATCAGCAGCAGCCAAATGAAAAGCTATTCTCCAAGT
TTTTCACCTTCCCACAATACTCAACTTGTCCGAGCACCACCACGTCG
GCCGCCGTGGACGCGGTGGCGGAGCGGCGGTCGAGCATAGCCGAC
ATCGAGGTGATGATGGTGGAGAGTCATGCAAACATCAAGCTATCGAC
AAAGAGGCGGCCGAAGCAGCTACTCAAATTAGTGGCCGGATTTCAG
TCTATAGGCCTTACAATTCTCCATCTCAACATCACAACCGTTGATCAG
TCAGTTCTCTACTCTCTCAGTGTAAAGGTGGAGGTTGAATGTCAGTT
TACTACAGTAAATGAGATAGCCACAACTGTGCATGATATAGTGGGAA
TGATCCAAGAAGAGTCAATTTGTAGCTAA

SmbHLH41 ATGGATCCGCCTTTGGTAAATGAGGCGTCGTTTACCGCCGCCGCGAA
CCCCAATTCGTACAGTTTGGCGGGGCTGCTTTCCTTCTCCGGCAGCG
GCGGGCTGGGGCTCAGAATGGGGAACTTGGGGCGCAGCGGCGGAG
GAGACGCGTCTCTCGAGGAATCGACGGTTACGGAGCAGAGCGGGA
GCGGGAATGGCGGTGGCCCGAAGCGGAGGAGGGATGTTGGGAGCT
CTTTTGATGACGACTCCTCCAAAATTGTCTCCACCAGCAGTGCTAAT
CAGGACGTGACTGATTCGAATGCCAAACGAGCAAAAGTATTTGATTC
CGGAGATCAATTGAAAACGGAAACAGAAACACATTCGGGGAGCAG
CAAGCCTGTTGAGGAGAAAAGTAAACCTGAACCGCCTAAAGACTAT
ATTCATGTTAGAGCAAGACGGGGTCAAGCTACTGATAGCCACAGTTT
AGCTGAAAGAGCTCGGAGGGAAAAGATCAGCGAAAGGATGAAAAT
CCTGCAAGATTTGGTACCGGGATGTAATAAGGTTATCGGAAAAGCTC
TTGTTCTTGATGAAATAATTAATTACATCCAGTCCCTACAGCGTCAAG
TTGAGTTTTTGTCGATGAAGCTTGAAGCAGTTAACTCGAGGTTGAGT
CCCACATTAGAAGGGTTTCCCTCCAAAGATCTAGCAGGACCTGCATT
TGATGCCAACGGGATGATATATGGACCACAAACACCGGGAGTGTACG
CCAGAGGATCGCAGGCGGAGTGGCTTCACATGCAGGTTGGAGGTGG
TTTTGAAAGATCAACATGA

SmbHLH42 ATGGACTCCATTTTTCACCTTGAGAATGGTGATCGCGCCGCCTTCCTC
AAGCAAATGATGCAAGCTTTTGGGTGTGTCTACATCTGCCTCAGGTG
CTATGTACCTCCACCATACAACTGTTTGAAGGGGTTGGATGGAATCT
TCCATGAAACAAGCACATCGCATGGAAGCCTTGCTAGGCGCCTTTTT
CATGTATATCGAGAATCATCCACCTATCCCGATTCCGGGCTAATCCCG
GGGATTGCTTTCGCGAGCAGTAATCCCTACATGAAGCTACAATCGCA
TGAATTTCACGGCCTAGTTTCCAGTGATTTCCAGCTTCAGTTCTACCA
AACGATTGTTCTCATGGGATGCGCCACCGGAGAAATTGAGCTTGGA
ATGTCAGATCATAATCCTCAGGTAGACGTGGAAACGGAGATGAAAA
ACTTAATCCGAATCCATTTCCCTCATCACAACGATCCAAATCAACCAC
CTTCATCTTCTTCTTCATCTCTGCTGTCACTCTCAGTCGACAGCCCCG
AGTACCCGCCTTATCTCTTCAACATTCCCACCACCACCACCACCGCC
GCCGCTGCCTTCCTCCCACAACAAACCCTCATCTCCTCTTCATCAATT
CCCCCGCACGACCAAACCATACAGATCCTCAGCCAATTCCGGAACG
CGCAGTTCCCCTCAATCGAGAGCGAAGACGCCGCAATGACGAAAGC
CATTCTAGCCGTGCTCTCCTCTCCTTCAACCTCCTCTACCTCTCAGCA
CAAACACATCCCCACCGCATTCAGAAGGTACCAAGCCAACTTGGCG
CCGCGGTTCGCCTCCACGCCTCAGAACCATAGCAGGTTCAAAAGAG
CCGTTTCGTTTTTCAGAAAGCTGAGCCTCAGAAGCAGACATGAAATT
CAAGGAAACCAGCCCACCGCTACGCAGCTGCACCACATGATCTCCG
AGAGAAAGCGCCGCGAGAAGCTCAACGACAGCTTCCAGATACTCA
GATCCTTCCTCCCTCCTGGATCCAAGAAGGATAAGGCGTCTGTGCTG
AGCAGCACCACCGAGTATCTGAGCTCGTTGAAGTCCCAGGTGGCGG
AGCTGGCCAAGCGGAATCTGCTTCTTGAATCGCAGATTAACTCCATA
CAGAAATCAGAAAGTGGCACAGACGAAGCGGGGAGTTCTTCGTCC
GGCGGCGGAAGGATTAGTGTGGAGATCGCGCAGGTTTCACTGTCGT

| | |
|---|---|
| | CGTCGGAAGCAAGATTCCTGGACTTGAGAGTGAGAGGAGAATATTC<br>TAGCTTGTTGGATTTGGTTGCTAGGCTGATTGAGTTCTTGAGAGGGC<br>AGCGCAGTGTGAGTTTGAGGTCGGTGGAATCGAATACGAGGATGTT<br>GGGATCAGTTGCAGTTCACGCCGTCATCATGAGACTCGGAGTTGAG<br>GGAGATGATGAATTTGACGAGTCTGGTTTCCGAGAAGCTGTGAAAA<br>GAGTTGTGGAAGACCAAGATAATTGA |
| SmbHLH43 | ATGAACCAGTGGGGTGGTAGCAGCGCCGCCGCAGCCATGGACGGCG<br>ACTCGGCCGTGATGGACGCCTTCATGTCCTCCGCCTCCGATCTCACT<br>TCCTTCTGGCCCCAACCCCAGCAAACCCCATACCCCCTCACCCCTTC<br>CTCGCCTCCCCCCGCCGCCGTCGCCCAATTTTTCAACCAGGAGACTC<br>TTCAGCAGCGCCTGCTCTCCCTGATCGAGGACGCCGCCCACGAGAG<br>CTGGACCTACGCCATCTTCTGGCAGTCCTCCGTCCGCCGGTGCTCGG<br>CTGGGGACGGCTACTACAAGGGCGAGGAGGACAAGGCGAAGCGCC<br>GGACGGCCACGTCGCCGGCCGAGCAGGAGCACCGGAAAAAGGTCC<br>TACGGGATCTGAATTCGCTGATTGCGGGGCCTCAGGCCGTCGCCGAC<br>GAAGCTGTAGACGCCGTCGATGAAGAGGTCACCGACACCGAGTGGT<br>TCTTCCTCATATCCATGACTCAGAATTTCGTCAACGGGTCGGGCCTCC<br>CGGGCCAGGCCCTCTACACGTCGAGCCCGGTCTGGGTCACCGGGCC<br>GGAGCGGCTCGCCTCATCTCACTGCGACCGGGCCCGCCAGGCCCAG<br>GGATTTGGGCTGCAGACGCTGGTGTGCATACCCTCACCAAACGGCG<br>TCGTTGAGCTCGGATCCACGGAGCTCCTGGTCCTTGCCGGAGACCG<br>ACCCGGCGGCGCTGTGGCTCACGGAGCCGTCTTCCTCCGCCGTGGA<br>TGTCAAGGATTCGTGATCAACAACCCTAATCAGGGAAGTTCAATCCC<br>TTCTTCTATTACTAGCAATCAATCTCATTTAGTTAAAGAAAATCCTAAT<br>TCCACCACCATGAATGAGAACCAAAATCTAGGATACGTCTCTAGAGA<br>GTTGAAATTCTCCGAATTCGGATTCGATGGTAGTAGTAACCCCAAAA<br>ATGGCGGTGGTTTTAGGCCTGAATCTGGTGAGATACTAAATTTTGGA<br>GAGAGCTCTAAGATCAATTCTGCAAATGGCAATGTGAATAATCTGTT<br>TGGGATTGAGAATGAGAATAGCAATAACAGAAACAAGGTGAAGAAA<br>AGGTCCCCAACTTCTAGAGGTAGTAACGAGGAAGGCATGCTTTCCTT<br>CTCATCTGCTGCGATGAAGGCCGACGGTGGCATCGACTCGGACCAC<br>TCCGATCTCGAGCCATCCGTCGTGAAGGAGGTCGAGTGCAGCACCG<br>TGGTTGATCCAGAGAAACGGCCTAGGAAACGGGGGAGGAAGCCCG<br>CCAACGGGCGGGAGGAGCCCCTCAACCACGTGGAGGCGGAGAGGC<br>AGCGGAGGGAGAAACTCAACCAGCGGTTCTACGCTCTCAGAGCCGT<br>TGTGCCCAACGTGTCCAAGATGGACAAAGCCTCCCTCCTCGGGGAC<br>GCCATTGCCTACATCAACGAGCTCAAGTCGAAGCTCCAGAACGTGG<br>AGATCGCCAAAGATGATCTCAGAAGGCAACTCGAGTCGTACAGCAA<br>GGAAGGTGGGGGGCGTCACTCGGTCCCAGCCCCCCCAGAGCGCGA<br>GACCAAGGTGTCCGGCAGCAATGCCATGGTCGACATAGACGTGGAC<br>GTGAAGATCATTGGGTGGGACGCCATGATACGCATCCAATGCAGCAA<br>GAACAACCACCCCGCGGCCAAGCTCATGGTGGCGCTCATGGAGCTG<br>GACCTCGACGTTCACCATGCCAGCGTGTCCGTGGTGAACGATCTCAT<br>GATCCAGCAGGCGACGGTGAAGATGGAGGGCCGGATTTTCACCCAA |

| | |
|---|---|
| | GATCAGCTCAAGCTAGCGTTGATATCCAAAGTTGCTGAAACTCGGTA<br>G |
| SmbHLH44 | ATGGAGCAGCTTACTCAACACAGTAGTCTCTTAGAGGAGCTAATCAT<br>GCCTCCAAAGATAGAGTCCTCTTTCTCAACCGAATTCTTCCAAAACA<br>CTTGGAACTTCACTCCTTTCGACCAAACCCCAGATTTCAACCTACCA<br>TTCACAAATCCTTCGCTCCTCGACCTCATCTCGCCGCCCGAATTCGC<br>CACCCCTTGCCCCTTGGGCGAATTCCAGCCCTTTCTCGACGCCCTTG<br>CCTCGCCGGACTTCGGATCCTTGTACGACAGGGACGATCTGCCGCCA<br>ATGTCAATTCCCTTCCTCGACGGCTGCATCGCCGATTTTGACGGCAC<br>CTTCAAACTGGAGAATTCACAAATTAGTGATGCGATAAACATCGATG<br>TCTACAACAATGTAGATCTGTACGGCGAGAGAAAGAGCAAATCGAA<br>GAAACCGGACGGCCAGCCGTCGAAGAATCTGATGGCGGAGAGGCG<br>GCGGCGGAAGCGGCTGAACGACCGCCTCTCCATGCTCCGATCGATC<br>GTGCCGAAGATTAGCAAGATGGATAGGACGTCTATACTCGGCGACAC<br>CATTGATTACATGAGAGAATTGCTTGATAAGATTCAAAAACTAAGGG<br>AAGAAGGCGTCGATGAAAGCACCGCCATTCGGAACTACTTCAAGGA<br>GCAGCAAATGCAGCTGAGAAACCCTCCAAAGTTTGAGGTAGAAAG<br>GAGAAGGTCGGATACCCGAATAGAAGTGTGTTGCACGGCGAAGCCG<br>GGGCTATTGCTGTCTACGCTCGCCACGCTCGACGGCATAGGCCTCGA<br>CTTACAACACTGTGTAATCAGCTGTTTCAACGATTTTTCTCTGCAAGC<br>TTCTTGCTACGAGCAAGCGTTGTTCAGAAATGCAGGCTACGGAGGG<br>AGATGTTTGTAA |
| SmbHLH45 | ATGTATGGTGATTCCCATGCATTATCTTCTGATCCGAGCCCTATATTTC<br>CACCGAATCGCAAGCAGAAAGAAGAGCAGCAGCAGCTTCCATTAAT<br>GGAATCCGACTACCGACAGCAGCTCGCCTGCAGCCAGAACAGTTCT<br>GGGCTGCTGAGATTTAGGTCTGCTCCCACTTCTCTGCTCGAAAATTT<br>CACAGACAAGGGTGCGAAATTGGGGTTTTCGAGATTCTGCTCCGAT<br>GAAATGGATGGCGTGAATGCGGCGGCTGAAAACGACGACGTTGATG<br>ATGGTAAGGGTTCCGGTAATAGATTTGGAGGTGCGAATTCGCAGCTG<br>CCGCCGCAGTATCCGAGGCAAAGTGGAGCTCAATTGGGGTATAGAG<br>GTCTGACCTCCATTGCGCGGGAAAATCAGGGGCTTATGCGGCAGAA<br>CAGCTCTCCTGCTGGATTGTTCTCTCACCTCAACTCTCAAAATGGGT<br>ATTCAGGGGTGAGAGGTGTTGGAAGCTACAGAGTGGGTGGTGATGG<br>AGATTTAAGCCCGTCTTCGAATAGGTTCAAGAACCACATGAACTGCT<br>CTCGTGTGTCGCCCTCTTCCTTATCTCGTATCTCAGAGTTTGAGAGCG<br>AGAATGCCCTTGTTGATGAGGCAAAGGTCGGAAATGGCAGTGGTGA<br>TCACAGTTTAATTTTCAGTTCTGGATTCCCATTTGCTTCTTGGAACGA<br>TTCTTCGTATTTTGCAGAGAATTTCAATAATAGTGGCATCAAAAGGG<br>AGCTGGATTGTGATCCGAACCTGTTTGTGAATAATAATGAGAGATGT<br>GAGACTGGAAATAGGCCAAATATTCTGTCACACCACCTAAGTCTGCC<br>TAAGACTTCATCAGAAATGGCTGCTATGGAGAAGCTAATGCAGCTTC<br>AAGACACAGTTCCTTGCAAAGTGCGTGCCAAGCGTGGCTGCGCCAC<br>TCATCCACGAAGCATTGCAGAGAGGGTTAGGAGGACTAGAATCAGT<br>GAGAGGATGAGGAAGTTGCAGGAGCTTGTTCCAAACATGGATAAGC |

AAACCAACACATCAGACATGTTAGATTTAGCTGTCGAGTATATCAAA
GCCCTCCAGAAACAATACAAGACACTTAGCGATGTTCGAGCAAATT
GCAAGTGCTTAGCCTTTCAAAAGCGTTAG

SmbHLH46 ATGCAGCACCAACACAACGTCACCACAGATGCATACATAAATGGAG
CTCGCCGCCGCCTACGCCGACGTCTCACACATCCTACCCGTGGACCC
TCAACCCGGCCCACTGACCCTTTCCTCCTCCCGCCGCCGCCGCAGCA
GGCAGCCTGCGCCGGCCTCTTCGGCAGAAGGCCGTCGTTCGGCTAC
GACGGCGACCACCACCTGAGGTTCCTCACAGAGTCGTTAGGGCAGG
TGGTGCACCCCGGCGGCGGCGGCGCCCCCTTCGGCCTCCACGCCGA
ACTGCAGAAGATGACGGCGCAGGAAATCATGGATGCCAAGGCCCTC
GCCGCGTCCAAGAGTCACAGCGAAGCTGAGAGAAGAAGAAGAGAG
AGAATCAACAACCATCTTGCTAAGCTCCGAAGCTTGCTCCCTAGCAC
CACCAAAACAGACAAAGCCTCACTGCTGGCTGAAGTGATCCAGCAC
GTGAAGGAACTGAAGCGGCAGACCTCCCTTATAGCGGAGAGCAGCC
CCGTCCCGACGGAAAGCGACGAGCTAACCGTGGATAAGGAGGCGG
ACGAGGAGGGCAGAGCGGTGATCAAGGCGTCCATCTGCTGCGAGG
ACCGCTCTGATCTCTTGCCGGATCTCATCAACACGTTGAAAGCCCTC
CGCCTTCGGACGCTCAAAGCTGAGATCACAACACTTGGTGGGAGAG
TCAAGAATGTGTTGTTCATAACCGGAGATGATGAATCCACCACTTGT
GATCACCACTCCATCCAAGATGCGCTTAAAGCAGTTATGGATAAAAC
TGCTGAAGACTCCGCTTCGGGGATGTCAAGAGGCAAAGAACCAATA
TCAATATCCTTCAACACAGGTTTTGAGAAAGTGACGGGTTTGTTCTC
ATCCCCACCGCCACGTACGGTCTACTCCTCCGCCGCTGCCGCCGTGA
GGGGAAGTCGTCGTTTGTGGGAATGTATATAG

SmbHLH47 ATGAGCAGGAACAGCAGCTATCTTCTCCCAAATCCGAGTAATGACGG
CGGTTTCAAGAGCAGAGATTCGATGGGGTCCGATTTGTTTCTGAATC
AGCAGCAGAGTAATCAGCAGGGGTTAGCGCGGTATCGATCGGCGCC
GAGCTCGCTCCTCGCTGCGCTTTTGGATTCGACTACTGAAAACAGCA
GCAGCGGCGACGAATCGGAAGCTCTGTTGTCGGCGCTGATGGATGG
GCCGCGTGATCTGAATCAGATTCAGAAGGGCGGTAATCCGATGCATT
ATCATCTGAAGCAGGAGGTCGGGGCCGAATCGGAGCCCCGACCCGG
TCATATGGGTTATGAGAGCGCTAGCAGCTCTCCTGCTGGATTCTTCAA
TGGATATGGCATAATGGGGGAGGTGGAGGATTACAGAGTGCAGAAC
CATTCAAAAGCAAGTTCATCAGCAGCAGGTGGTTTGAGCAGTCACA
TTAGCTTCTCATCCTCGAGGTTCATGCCTAGCATACCTGAGAACGTG
AACGAAAGCATTGCCAATGCGACGCGCAACCCTGAAAACGGGCAGT
TGGGAAGCGCCAACGCCAGAGAATTCGACGCCCTCTTCCCTCAGGA
CTCGTGGAACGACACTCCTTTCAGCAGCTTGAAAAGAAATCGAGAT
CATATGTTTTCCAATTTCAATGGATTGGAGAGCCAGAGTGGAGAAAC
GAGGAGAAGCCCTCATGGTTTAGTTCACCACTTGAGCTTGCCCAAA
ACTGCCACTGAAATGGCCGAATTGGACAAGTTTCTGCAAGCTCAAC
CGGACACGAGTCCCTGCCAGATTAGGGCGAAAAGAGGCTGCGCCAC
TCATCCAAGAAGTATAGCAGAGAGGGTGAGACGCACCAGAATCAGT
GAGAAGATGAAGAAGCTGCAAGACCTTTTCCCAAATATGGACAAGC

| | |
|---|---|
| | AAACGAGCACAGCTGATATGTTGGATTTGGCAGTTCAATACATTAAA<br>GACCTTCAAGTGCAAGTAGAGACGCTGACGGAGATGAAGGCCAAG<br>TGCGTTTGCTCCATAAAACCTCAACAAACAGTTGAAGGAGCGCTCT<br>CCGTTGTCGATGTTGGTAGGCTTCCACCAACAATGCGGATGTAA |
| SmbHLH48 | ATGGTTTCGCCTGAGAACACGAATTGGCTCTACGATTATGGCTTTGA<br>GGATATCACTACGTCCACGGACGGCTCTCACTCCGGGGAGCCTCCGA<br>TCGACCCAGGCTGGTTGGCTTCAAGCCCCCTGGAATCGTGCGGCAC<br>TCTCCCCAGTGTCGAAATTGATGGATCATTTGGAGAATCTGACTCGC<br>AGAAGGAAACTGGGTCCAAAAAAAGAGCTAGAACTGAGACATGTG<br>CTATATCAAGCTCTAAAGCATGCAGGGAGAAACAGCGAAGGGACAG<br>GCTAAATGACAAGTTCATGGAATTAGGTGCTCTTCTTGAGCCTGGGA<br>GACCTCCTAAAACTGATAAAGCTGCAATCTTAGTTGATGCTGTCCGA<br>ATGGTAACTCAGTTAAGAAGTGAAGCTCAGAAGCTGAAAGACTCAA<br>ATTCGAATCTGCAGGAGAAGATCAAGGAGCTCAAAACCGAGAAGA<br>ACGAGCTTCGTGATGAAAAGCAAAGGTTAAAGGCGGAGAAGGAGA<br>AGCTGGAGCAGCAGCTGAAGACAATGAGTGCTCCCCAACCTGGTTT<br>TTTACCAGCTACCCCAAACATCCCAGCTGCATTTGCCGCCCAAAGTC<br>AAGCTGGCGGCAACAAGTTGGTGCCAATCATCAGCTACCCTGGTGT<br>CGCTATGTGGCAGTTCATGCCACCAGCAGCCGTTGATACGTCTCAGG<br>ATCATGTGCTTCGCCCACCGGTTGCCTAA |
| SmbHLH49 | ATGTTGACGGATCAGAGATCGAACGACGATTCGCGGAAGCGGCGGG<br>ATGGAGATGAGCATTGCGCCAAGGGAGGAGGAGCTTCCACCAGCAG<br>CTGCAACAACAATAACAACGTCTCGAATGAACGAAGGAATGAAGGT<br>GATAGTAAACGGATGAAGGCAGTGGGTGAACTTAAAGTGGAAGGGG<br>AAGGGAATTCTGGCAAGGGGGCGGCCGCGGTGGAGAGACGCTCGA<br>AGCTAGACGAACTGCCGAAGCAAGACTACATCCATGTTCGAGCAAG<br>ACGAGGTCAAGCTACCGATAGTCATAGTTTAGCAGAAAGAGCTAGG<br>AGAGAAAAGATTAGTGAGAGGATGAAAATTCTTCAGGATTTGGTTC<br>CTGGTTGTAATAAGGTCCGAGCTGCTATCAAGGTCAAGGCATCAGTC<br>ATCATCTGTTTCACTTCTTCCAGGAAGAGCTGCTAG |
| SmbHLH50 | ATGTCTCACATAGCCGTCGAGCGAAACCGCCGGAGACAGATGAACG<br>AACACCTCAAAGTTCTACGTTCCTTAACCCCATGTTTCTACATCAAA<br>CGGGGCGATCAAGCCTCGATCATCGGTGGGGTGATCGAGTTCATCAA<br>GGAGCTGCATCAAGTGGTGCAGTCGTTGGAGGCGAAGAAGCGGAG<br>GAAGAGCCTGAGCCCTAGCCCGGGGCCGAGCCCGAGGCAGTTCCG<br>GCTGAGCCCTCCGCCGGAGACCCCCTTTTCCGATGACTTCAAGGAG<br>CTGGGAGCTTGCTGCAACTCTCCGGTGGCTGACGTCGAGGCGAAGA<br>TCTCGGGTTCCAACGTGATCCTACGAACCATATCGAAGCGGATCCCG<br>GGCCAAATCGTGAGGATTATCGGAGCCTTGGAGAGGCTTTCCTTTGA<br>GATTCTTCACTTGAACATTAGCACCATGGAAGATACTGTGCTTTACTC<br>CTTCGTCGTCAAGATAGGGTTGGAGTGCCAAGTGAGTGTGGAGGAG<br>CTAGCATTTGAAGTTCAGCAAAGTTTCTGCTCTCAAGCAGTCTCCAT<br>TATGGAACATGCACAAATTTTGTCATGA |
| SmbHLH51 | ATGGCTACTGCAAATCAAAAGGGTGTTCCAGATAATCTCAGAAAGCA |

GCTTGCTCTTGCAGTTAGAAGCACCCAATGGAGCTATGCAATTTTTT
GGTCTTTTTCTGCTAAACAATCTGGTGATGGGTACTACAACGGAGAT
ATTAAGACAAGGAAAACGGTTCAAACCGTAGAGCTGAACTCAGATC
CGCTGGGATTGCAGAGGAGTGATCAGCTAAGGGAGTTGTTTGAGTC
TCTTTCATTAGGTGAGACCACCCCACAACCTAAGAGGCCTACAGCTG
CATTGTCCCCTGAAGATCTCACTGACACTGAGTGGTATTTTCTCGTGT
GCATGTCGTTCGTGTTCAATAAAACGAGACAATCTGGCTACGCAATG
CTCATCTTGCAGACACGAAGCTCTTCTCTCGTTCTTTGCTTGCTAAG
GCAAGTTGCATCTCTGCAGACAGTAGTGTGCTTTCCACATTGGGGAG
GTGTGGTTGAGCTCGGAACAACTGAACTAGTTCCAGAGGATCGCAG
TCTGATTGAGCATATAAAAACTTCGTTCTTGGAGAGTTCTTCCGATGC
TGTCATCAATCCGAGCCATGAACTTGTCTATCAAGTTCTCAATCACTC
TGACATCCCTGAAAACAATCTTGATGAAGTGGAGGTTTATTCTCCCG
ACACCAGCTCAGATGAATTCGCAGACAATGTGCTGATAGAGGGATC
AAGTTTAGCAGACGGCGCTGATGGGGAGGCATCTCAGTTGCAAAGC
TGGCAGTGCAAGGACGATGCTGACCCGGTCAGCAATGGCACGAACA
ACTCCACGAGCTCTAGTGACTGCGTGTCTCAGATTCATGGGAATCTG
GAGGTGAAAATCCAGGAATCCGATGGAAAGAAGGCGGGTGATGCTA
ACTGTGTGCCCGTAAGTCAAGAATGCAACCAGCAGAAATCGCCTAG
CTTCAACGGTGGTGGTGGCGGCAGCGGCGGCGGCGGTGGTGGTGGT
GTTGGTGATGTTCATTATCACAGTGTAATTTCCAGCCTTTTGAAGAGC
TCCCACCAGCTGATTCTGGGGCCTTACAGAAACGGTAAAACGGAAT
CGAGCTTCATCAACTGGAAAGACAGGAAATTGAGTCATCGATTACCC
CAGAGCGCCTCGCCACAGAGGTTGCTAAAGAAAGTGCTGTTTTCAG
TAGCTAGAATGCATGAAAATGCCCAGATTGAATCTGCCAAACACAAG
GACAGATGTGATGACCAATCCGGACAGCAAGAAGGCGAGGAAGTC
GACAGGAACCATGTCCTGTCCGAGAGGAAACGGAGGGAGAAAATC
AGTGAGAGGTTCGCCATTCTCGGATCACTAGTTCCATCTGGTGGCAA
GGTTGATAAAGTATCAGTACTTGATCACACAATAGAGTATCTGCGAG
AGCTAGAGAAAAAGGTAGAAGATCTGGAAGCTTACAAAGAAGCAA
CAGAACGAGAGTCGACCACGCAGAGCAAGGCTCACGATGCCATCG
AGAGGACCTCCGACAACTATGGTCAAAGCAAGCCTGGCAGCTTCAC
GAAGCTGCTTGGGAGCAAGAGAAGAGCTAGTGACATGGAGAAAAC
TGCACCCGAGAATAAGAGGACTCGGTCGAGCTCATCTACAGACAGC
ATAACCATCAGCATCACAGACAAGGATGTGCTGGTTGAGATGAGATG
TTCTTGGAGGCAATGTGTGTTGATTCAAGTTATGGAAGCTTTGACTC
AACTGAATCTGGATTCACAATCTGTTCAATCTTCCAACACTGATGGA
ATTCTCTCCCTCTCTATCAATGCCAAGAGCAAGGGAGTGAAAGGTGC
GTCAGCTGGTGCAATCAAACAAGCTCTTCAGAGAATTATCAAAAAG
ATTTGA

SmbHLH52 ATGAATAGCCATGAGCAGCAATTCCAGCAAAATACGCAGATGGGGTC
GGGGCTGACCCGATACAGGTCGGCCCCGAGCTCGTATTTGACTAGCC
TTCTCAGCGCTCCAAGCAGCGATGGCGGATTCGGAGAGGAAGATTT
CGCCGAGCTCTTCAACCCGCGAGCTTCGAGTCCTGAGACGCAAGTA

ATTTTCTCGAGGTTCATGAACAGCTCCGGACAAGAATCCCGTCCCTC
AATTGCGCCTCTGCCGAGAAAGTCGGAGAATCAAGAGTATGCGCCC
CCGCAGAAGCTGCAGAGGCAGCATAGCCATGATTTGTATTCCGAGGC
TGCTGCTGTTGCCGACACCTCCTACAGCAAAGTGTTTGCGCCTTTGA
ACTCCAATTGCGCGGCGCCGGTGCAGGTCAAGATGGAACGCACCTC
TGGCCTCATTCGTCACAGCAGCTCGCCTGCAGGCCTCTTCGCCAACA
TAAACATCGAAAATGAGTTCAAAACAATGAGAGGCGTGGGCAGCTA
CGGAGGCGGTAACAGTGCTAATTCGGAAGCAACATTTTCTTCATCGG
CAAACAGGTTTGTGGCCCAAATGGACTACCCCTCTTCTTCGGCGATG
AATATGATGAACCCTATTTCAGAAGGCATTGGAGAAAATGCAGCAGT
TGATGACGATGATGTTGACTTCATCACTAGTTTACCTTGGGATGATTC
ACCACTCTTGTCTGATCCTTACCTGAATGATCAGAAAAACGAAGCAG
GAAATCGGCCTAAAACTCTGTTGTCTCATCACCTAAGTTTGCCAAAA
GCAGCAATGGAGAAGCTGCTGCAAGATTCAGTTCCTTGCAAAATCA
GAGCCAAGCGAGGCTGTGCTACTCATCCTCGAAGCATTGCTGAAAG
GGTTAGAAGAACTAAGATCAGTGAGCGAATGAGGAAGCTGCAAGA
ACTCGTGCCTAATATGGAGAAGCAAACCAATACATCTGATATGCTGG
ACTTGGCTGTTGATTACATCAAGGATCTTCAAAGACAAGTAAAGACG
CTTTCGGGTAATCAAGCCAAGTGTAGCTGCTCGGCTAAACAATGA

SmbHLH53

ATGAGGAAGGAATCTAGTATGGCGGGAATTGGGTGGAACGATGAGG
ATAGGGCTATGGCGTCGGCGGTTTTGGGGACTAAGGCATTTGATTAC
TTGATGTCGAATAATGTTTCCGCCGAATGCTCGTTGATGGCGATGACT
AATGATGAGAATTTGCAGAACAAGCTCTCAGATCTCGTAGAGCGTCC
CAATTCCGCCAATTTTAGTTGGAATTACGCGATTTTCTGGCAGCTTTC
TAGGTCGAGGGCGGGGGACTTAGTGTTGGGGTGGGGGGATGGGTGC
TGTCGAGAGCCCCGTGAGATCGAGGAGGATTCGGATGTCACTCGTTT
CCTCAGAATCCGGCAGGAAGACGAGTGCCAGCAGCGGATGAGGAA
GAGGGTTCTGCAGAAGCTGCATATCTCGTTTGGGGGGAGCGAGGAC
GAGAGCTACGCCTTTGGATTGGATAAGGTCACGGATGCTGAGATGTT
TTTCTTGGCATCTATGTATTTCTCATATGCTAGGGGGGAGGGGGGGCC
GGGGAGGTGCTTTAGCTCCGGTGAGCATCTCTGGTTGAGCGATGCGT
TCAAGTCCCCCGTGGATTACTGCGTTCGCTCCTTTCTTGCGAGGTCT
GCTGGCGTGCAGACGATTGTGTTGGTTCCCACTGATATTGGTGTGGT
GGAATTGGGTTCGATTAGGTGTGTCCCGGAGAGTTTGGAGCTCGTG
AAGGTTGTTAGGTCGTCGTTCTCCTCGTTGTCGGGGGTTCTCCGGTC
CAAGAAAGCTGTGGCTGTGGCTGTGGCAGCGGAGAACGACAGATC
AAACCCGAAATCTGGTTTTGGAGAAGTTATTCCCAAGATTTTCGGGC
AAGATTTGTGTTCTGGTCGCGTGGAATTGAAGGAGAAAGTCGATGTT
AGGAAGGTTGGATACGGGGCGATGGATGCAAGTGCGAACGGGAATG
GGAACGGGAACGGGAATGGACAGCCATTCGCCAACGGTTTCCATGC
CATGGTGTGGAGGCAGTATGGGAATGTTAACGTTAAGCCTGCGAACC
CGGTGGAGGTTTGTAAGAATCCTCCGAGACCAACGAAACATGTTAAT
GATATGGTGAATGGGGCGAGGGATGGCCAGCATCGGAAACCAGCAC
AAATGCAGATCGATTTTGCTGGAGCAACGGCAAGACCCGTTAGCTC

CAGACCACGCAGTGCAGAGTCGGAGCACTCTGACATTGAAGCTCCG
TGCAAGGAGGAGGGCATGGTGGTGGTATCGGAAGACAACAGGCCTA
GGAAGCGTGGCCGGAAGCCTGCCAATGGGAGGGAGGAGCCCCTCA
ATCACGTGGAGGCGGAGAGGCAACGGCGGGAAAAGCTGAACCAGC
GCTTTTACGCGCTGAGAGCCGTTGTCCCGAACATATCCAAGATGGAC
AAAGCTTCCCTCTTGGGAGATGCTATTGCTTACATAACCGAGCTTCA
GAATAAGCTCAAGGAGTTGGAATCCAGTAAGGAAAGTAGGCTATCG
AGAGAGGCATCCGTCTCGGAAACTCAGCCGAATACGGAAACGCCAG
CCTGCATCCCGAGCATCGACATACAAGCAGGACGAGAGGAAGTCAC
GTTGAAGGTGAGCTGCCAGCTGGATGCTCACCCCTATCGAGCGATCA
AGGATGCTCGGGCGACCATAGTCGATGCCAAATTCGCATCCGGGAGC
GAGAAAGTGTTCCACACGTTCGTTGTGAAATCCGATGCACCGGAAC
GGCTCACCAAGGAGAAGCTGCTAGAAGCGATCTCCAACTCGCAGCA
GCAGTCGTCGTCTGTCGGGTAA

SmbHLH54

ATGCTAATGGACTTCCCTTGGAATTGGATAATCTCACAACTGCAACC
CAACCCCCACCCTTTCTATTGCCGCACCAACAACAACAATCCCAGGC
GCCCTTGTTCCACTTCCCCAACCGCCACAAGCTCTACGATTACTCGC
TGCCGGAATTTGTCTTGTCCGCCGCCGTTGCCTCAGTTCCCTGCCGC
GGATTTCAGCACCAGGATCTGTGAGAACGCCAAGACGAACGAGCTT
ATCCGCGCAGAGCATCGCAGCCAGGCAGAGGCGGCGGAAGATCAC
AGTGAAGACGCAGGAGCTCGGGAAGCTGGTTCCCGGCGGGCAGAG
GATGAACACGGCGGAGATGCTGCAATCCGCCACAACTACATCAAGT
TCTTGCAGGCGCAAGTTGCTCTCCTTGAATTCCTTGGTTCGCATCATC
AGGAGGTACCATTCGAAGGTGAAGAAGAGCTCCAGAATCTTCTAGA
ATCTCCTTTGATTCAAGAAAAGCTATACTCCACTCAACATTGCTTGCT
TCCAAACAATGCTGGATTTTCAACTCTTGATTATCAGCGCTGGATGG
GCGCGCTGGATTTCGAACTTCCGGATCGGGCAGTCGCTGGATGGAAT
GCTCAGTCGTGCTTTTGGAGCTCGGATACTGCTGGGCTTGTTGTGTT
CACGTTGATACTGACGGGTGGTCTGGTGGCTATGATGGTAGACGTTT
CTTTCTTTGAAGGGTTGGTGATTGATGGAGTTTGGGTTGGGGTGTCA
GCGACGTCTTTTTTCAGTGATCGGGTGCGCTCGACACTGGGAGTCG
GCTGGTCGGACCTCTTGGATCTCCGTGATCGGCTTGATTCTGCTGTTT
CACGTCGAGCTTGTTTGAGGGGCGGTAGTTTTCGTAGTCGATGTGCC
GCTTCTTGGAGTTTTTATCTCCTTCTTCCGTCGGCTTGTGTTTTCTTG
GCATGGTTTCACGTGTTGTGGTGGGGGCAGGTAATCCCTTCTTCATC
AATGGCGTTGAGCTACTACTCCAACTGGACCTCTTTTCAGCAGCCAG
ACTACTCCGGCGATGATCCAGAGCTCCAATCTCTGTTGAACCCGGAC
GACTATTTCGCCGACTCCTTCTGCAACTCTCTTCTCTGCGACGATCTC
ACTTATCATGCTAATGGACTTCCCTTGGAATTGGATAATCTCACAACT
GCAACCCAACCCCCACCCTTTCTATTGCCGCACCAACAACAACAATC
CCAGGCGCCCTTGTTCCACTTCCCCAACCGCCACAAGCTCTACGATT
ACTCGCTGCCGGAATTTGTCTTGCCGCCGCCGTTGCCTCAGTTCCCT
GCCGCGGATTTCAGCACCAGGATCTGTGAGAACGCCAAGAACGAAA
CGAGCTTATCCGCGCAGAGCATCGCAGCCAGGCAGAGGCGGCGGAA

GATCACAGTGAAGACGCAGGAGCTCGGGAAGCTGGTTCCCGGCGG
GCATAGGATGAACACGGCGGAGATGCTGCAATCCGCCTACAACTAC
GTCAAGTTCTTGCAGGCGCAAGTTGCTCTCCTTGAATTCCTTGGTTC
GCATCATCAGGAGGTACCATTCGAAGGTGAAGAAGAGCTCCAGAAT
CTTCTAGAATCTCCTTTGATTCAAGAAAAGCTATACTCCACTCAACAT
TGCTTGCTTCCAAACAAGTTGGCGGAACAAGTTCCATTACTCAAATC
CAATCCACATTTGCTGGAAAAGGATCATTGA

SmbHLH55 ATGGAAGAGAGGTGTGGAGAAGAAACAATCCCAATCCCAATCCCAA
TCCCAATCCAAGAACAATATGCAAATCCCGATTTCGAGATGCCATATT
TCAACGAAAATTTAGATCACTTTCTCGATCTATTCTGCAAAGAGAAT
GCAGCCGATGATCCTATTCTCAACGAAATATACGACTTCCACACCCTA
AACGACGACGTTGCATTGATGAGTCCTCTTCCAGACTTTAATAGCAG
CATCAGCATGCCTAACCACCTTCAAAACGACGACGTACAGGATGAG
GATGAGGACGAGGACGAGGACGAGGATGAGTCCTCGGCAACCACA
TCGGAGAAGACGAAAAAGAGTGATAGATCCACCACTTTGATGTCGG
AGAGACGCCGCCGCCGCCGGATGAAGGAAAAGCTCTACACTTTGCG
CTCCTTGGTTCCTAATATTACTAAGATGGATAAAGCTTCTATAGTTGG
AGATGCAGTTCTGTATGTGCAGGAATTGCAAAAGCAGGCTAAGAAG
CTAAGATCGGAGATCGCCGGCCTCGAATCATCATTCACACGACTCAA
CAACAGTAAATCAAAATTCAAGAAAACAAATGCTTCCCCTACTCCCA
CAATCAAGAAGATTTTCAAGTTGGATGTATTTCAAGTTGAAGAAAGA
GGTTTTTACGTGCGAATAGTCTCCAACAGAGAAAGAGGAGTTGCCG
GATTTCTCTACAAAGCGCTTGATTCCCTCAAAAGATTCCAGATTAGG
AGCTCTAATCTGGTTTCTGATGCTGAAAATTATGTGCTGACGTTCACT
TTGCATGTTGTTGAAGAAGAGGTGGAGACGGACGTCTTGCCAAGTC
TGAAGATGTGTATTGGAAAGGCTTTTGTTGGTCAAGGATTTGATTTT
CAGACTTCCTCATCTGCTTAA

SmbHLH56 ATGTTTTCTATTCCTAGTTTGTATGATATGGGGACGTGTTCGGATTCAT
ACGACATTGTGGGAATGCTGAGTTCGCCGACAAACTCCGACAATCC
AAACCTCAGCCCAAGATCCATGGCGGAAGCCAAAGCAGTGGCCGCC
AGCATCAGCCACAAGGAAGCGGAGCGCCGCCGCCGCAAGAGAATC
AACGGCCACATCGCCACCCTGAAATCCATGCTCCCCAACACCATCAA
GACGGACAAGGCGTCCCTGCTGGGAGAAGCCGTGAGGCGCGTGAA
GGAGCTGAAGAAGAGCGCGGCGGAGCTGGAGGAGGGGCCGAGCG
AGGCGGACGAGCTGAAGGTGTGGCAGTGCGAGAAGACGGGGCTGA
TGAAGGCGGCATTGTCGTGCGACGACAGGCCGGAGATAATCGTGGA
CATGATAGAGGCACTGAAGGCGGCGGAGGCGAAGGTGGTGAGAGC
GGAGATGTCGACGGTGGGGGGGAGAACCAAGAGCGTGCTGTGGGT
AACCCAAAACGACGCCGGATTGGGCCCGCTGAGGCGGGCGCTGAA
AATGGTCATGGACAAGTCGACGTTGGAGGCGAGTTCGGGACAGGGA
TTGCCGGCCACCAAACGCCCCCGTTACTATCACTTGTGA

SmbHLH57 ATGGAGGTCGGGAATGCATTAGCAACTAGAAAAGTGCTCAAAGCAG
ATCGCGAGAAATTGAGACGAGACAAGCTAAATGAGCAGTTTCTGGA
GTTGGGCAACTTACTAGATCCAGACAGGCCCAAGAACGACAAGGGC

| | |
|---|---|
| | ACCATTGTCTCCGACACAATCCAAGTGCTCAAGGAATTGAATTCTGA<br>GGTGAAAAGGCTCAAGAAAGAGCATGCACTACTTTGCGAAGAAAC<br>ACACGAGCTAACACAGGAGAAAAACGAGATCAGGGAAGAGAAAGC<br>TTCTCTGAAATCCAGTATTCAGCAGAACCTCAGAGCTCAGCAGCATC<br>AACAAACTCAAGGCTTTGTGGTTCCGTGGGATTCTCAACTACTGATG<br>GCACCATCGTATCCATTTCCTATAGCTGTTCCCATGCATCATCGAGCA<br>GCACAGGTGCCACCTTTATTTGCCAATGCCACACCAACGACTTACAT<br>TCCTTATCTTACTCCTGCTATGCCTAGAGTCGACCAGCCCGCTGCCTT<br>GAATGTGTCAGCTTCTGGGCCAAGAGCTGGAACTGCAAATTCCACT<br>GACGTTGCAACTGATTTGGAGCTTAAGATACCAGGATCTAAGGTTGA<br>ACACAAGAAGCTGTGA |
| SmbHLH58 | ATGGAGGCATACAGCGTGGCGATGGAGGAGGACTACGAGCCGTGGC<br>CCTACTACCTCCCGCCGCAGGCCGAGGAGGCCGACGACACCGACGA<br>GGAGCTCGGGGCCATGAAGGAGATGATGTTCAAGATCGCCGCAATG<br>CAGCCCGTCGACATCGACCCGGCCACCATCCGCAAGCCCAGGCGCC<br>GCAACGTCCGCATCAGCGACGACCCTCAGAGCGTCGCCGCCCGCCA<br>CCGCCGCGAGCGGATCAGCGAGAAGATCCGGATCCTGCAGCGCCTC<br>GTGCCGGGGGGAACCAAGATGGACACCGCCTCCATGCTCGACGAGG<br>CCATTAGGTATGTCAAGTTCTTGAAGCGCCAAATCCGGATCCTCCAG<br>GGGCCGCCCCCGCCGCCGCCCACGTACTTGTTTGGCGGGACTGATG<br>GCGGGCCGCCGCCAGGTGATGATCATCATCCCGTGTGTTTCTAA |
| SmbHLH59 | ATGGAAGAAATTCGCGTCTCTCCTACGCCGTCCGCCCTCCACACCCA<br>GCTCCAGTTCATTCTCCAAAGCCAGACTGAGCGCTGGGACTACGCC<br>ATCTTCTGGAAGTCATGGAGAGGCGTCGACGGCGGCGGCCGCCCCG<br>TTCTGTCGTGGGGCTCCGGCTACTTCCACGGCGACACCGTCACAGTA<br>CCTACAAACACCAAACCCGACCCCTCCGAATCCGAGTGGTTCTACAT<br>GGCCTCCATCACCAGATCATTCGCGGCCACCGACGACCTCGTTTTCC<br>GAGCTCACGCCACGGCCTCGAATGTCTGGTTGGTCGGACCCCAACA<br>GCTTAACCTCTGCGGCTCCGAAAGGGCTAAAGAAGCTCTCTTACATG<br>GACTCACAACTCTGGCTTTCATACCAACTCCTTATGGCGTCGTTGAAT<br>TAGGCTCCTCTGATTTGATCAAAGAGAACTGGAGCCTCATCAAACTC<br>GTTACCAACTCTTTTCATCCTCAAATCCAAGACCAGAAAAGAGTTCT<br>GTCTAAGACCTCGTCGGGGTCGCCTGAATCATGTGATTCACGCGACA<br>TCCCTCCACCAAGAACTGAAATTCAGTTGGTGGATGGATGGCAGGA<br>AGCGCAGGGAATGCTCAACCAACGATTCTACGCTCTGAGAAGCGTC<br>GTCCCCAACGTGTCGAAGATGGACAAAGCCTCTCTGCTAGCCGACG<br>CCGTCACCTATATCAACCAGCTCAAATCCTCACTCACCGCCTTAGAA<br>GCCAAATGCGCGCGTTTCCTCAAAATGTACAACGTTAGAAGCACATC<br>CGCAGCCAAGACCTCCCACGCCCACAAGCTGGAGATCGAGGTCAAG<br>ATTCTAGGATCGGAGGCGTTGATCCGAGTCCAATCCGACGACGTTGA<br>TCACCCTTGCGCCAGAGTGATGAACGTGCTCAGAGATCTGGATTTGG<br>AAGTGAGTCGAGCTAGCTCGTCAGCGATCGGAAAGATTATGTTCCAA<br>GAAATTGTTATTAGAGCTAGTGATGGTTTGTGTAGTGAGGAAGCTGT<br>CAAGACAGCTATAGTTGGGAGATTGGAGCTTATGACTCTGTCCCCAA |

| | |
|---|---|
| | TTTCATAA |
| SmbHLH60 | ATGTTCAGCTCAGACACCATCTCCAGAGACTTCCTTCACTCCAATGC<br>TAATCCCTCTCACCACACCAATTTCAAGCATGTCTCAGCTGATGCCG<br>AATTCTCCAAGAGCAGAGAGTTCATGCCCTCGGATTTCTTCAACAAT<br>CAGCATCAGCACAGCTCCGGACTAGCCCGATATCGGTCAGCGCCGA<br>GCTCGTTGTTCGCGGCCCTTCTGGATTCCAACACCGATAACAACAGC<br>AGCAGCGGCGATGAATCAGACGCTTTCTTCTCGGCGCTGATAGAGC<br>GCGATCTCAATCCCAAGAGTAGTGATCATCAGATTTCGAGCGGTATG<br>AAGCGGGAAGATGGGGCCGAGGCGGACCCCCGACCCGCGCAAAAC<br>GGGTATGATGCGGTTACCGGATCTTACTCTGTGGGGATGGTGCATCAT<br>GTTGATGTGAGGTTGAGGGATGAAAATGGGAATAGGTCCAATCTGCT<br>TCGTCAAAGCAGCTCTCCTGCTGGATTCTTCAATGGTTTTGGTGTAAT<br>GGGAGAGGCAGGAGACTACAGAGTCCACAATCCTGCAGAAGACAG<br>TTCATCAGTTGGTGGTTTGAGCAGTTCCATGAACTTGACGTCGGCTG<br>CATCTTCAAGCTCGAGATTCATGCCTAGCATACCCGAAACTGGAAAT<br>CAGGACGCGTTCAGCCCTGAAAATGGTCGTCTGAGAAACGAACCTA<br>CCTTCCAGCATGATTCCTGGAATGAAACATCCTTCAACAGTTTGAAA<br>AGAAACAGAGATGGTGATTCGAAGATGTTTTCCAATTTTAATGGATT<br>GGACAATGAGAATGGAGAAGCCAGGAAAAAGTCCTCTGGTTTAGTC<br>TCGCATTTTAGCTTGCCAAAGACATCCACGGAGATGGCTGCGGTTGA<br>GAACTTCCTGCAGTTCCAACAAGAGACGACAGTTCCTTGTCAAGTT<br>CGAGCAAAAAGGGGTTTTGCCACTCATCCCCGAAGTATTGCTGAAA<br>GGAATAGACGAACACGGATTAGTAAGAACATGAAGAAGTTGCAAGA<br>TCTTTTCCCCAATATGGATAAGCAAACGAACACGGCTGATATGTTGG<br>ACTTGGCAGTTGATTACATCAAAGAACTGAAGAAAGAAATGCAGAT<br>GCTCAACGACGCGAGAGCAAGATGTGTTTGTTCAGGTACACCTCAA<br>CAAACAAGTCCTACCACGTAA |
| SmbHLH61 | ATGTATCCATCGTCTACATCCTCTTCATCCCAGGGCTCAATGGGCAGC<br>AGCAGCAACGGCGGCGGCGGCGGTGGACTCATACGCTACGGCTCCG<br>CCCCGGGCTCCCTCCTCACCGCGGCGGTCGACTCTGCGACCCGCGA<br>ATTCTCGGCGCTCGGGCCTCAGGCTCAACCCGGCCCGGCCCGTTACT<br>TAGCCTCTGAATCCGCCGCAATGGCGAGGCGCGGCAATCCAAGTGG<br>CGAGGAGCACGCTACAGCTAACACCACGATTGGTTTGCAGCCCTCG<br>TACGGCTTCGGCGGCGGCGCGTCTTCTACTGCGTTGCTGAGGCAGA<br>GTAGCTCGCCGGCGGGATTTCTCAATCACTTAGCAACGGCGGCTGGA<br>GATAGAAATGCAGGATTTTCAGTCACGAGAGGCATAGGCAGCTACA<br>ATTCAAAGGGAATAGGTGAGACCGGCGGCGGCATATCCAGACTGAA<br>TTCACAGCTCAGTTTCACCGGAAAAGAATCGCTCTCTCGCATCTCCG<br>AAGAAGCTGAGGCCGCCGCCATGGACAACCACCAAAAGAAGTCTT<br>ATGCCACCACTAGCGGTGGCGGCGCTTTCGGAATGTGGGAACCCAG<br>CAGTAATCCTATAATGTTTTCAGTCGCGCAGTCGAATCGGGGCAAGA<br>ACGTTGGCGACGGTCTTGACGCCATCGAATCTCAGTTTCAGTTTAGC<br>ATATCGCAAACAGCGCAAGACATGGCATCAATGGAGAAGTTGCTCA<br>ACATCCCACAGGATTCTGTAACATGCAAGATTCGAGCAAAGCGCGG |

| | |
|---|---|
| | CTTTGCTACGCATCCTCGTAGCATTGCGGAACGGGAGAGAAGGACA |
| | CGAATCAGCGGCAAGTTGAGGAAATTGCAAGATCTTGTTCCAAACA |
| | TGGATAAGCAAACTAGCTACGCCGACATGCTGGATCTAGCAGTGCAA |
| | CACATAAAAACCCTTCAAGATCAGGTTGAGAAATTGAACCATGATCT |
| | TGATAGCTGCTCATGCGGATGCAAGAAGACGTGA |
| SmbHLH62 | ATGGAACACGTCGGAGCAGTAATTCCTCAAGGAGAGTGGAACTCCT |
| | TAAGCGGAATAATGTGCTCTGAGGAGGCTGAGTTCATGGCAGAATTG |
| | CTAGGGAACTGCTCTCTCCCAAATGAGGCGACATCGCCTGCAATTTG |
| | GCCGCCCCACCGGAACATTGAGGCTGCATTGTATTCCTCTGATGAAA |
| | CTATGTGTTTTTCTTTTTCACAAGGGAGTGGCAGTGGCTTTCTTTCCC |
| | CCTTTTCCAGTCAGGAAATTAGCTACTACTCCACTCTCTCTCATCATG |
| | CTTCTCTGCCTAACAACAATTACTCTACTAATGTAATGGGAGGGGCC |
| | GATGATGTCTTCAACGACAGTACAGAGTCTAACAACGACGTCGTGC |
| | CTGAAGAAGAGAAGAAACTCGGCAGCAGCCCTTGCAAGTCGAATA |
| | AAAGATCTCGTGTTCCCTGTGAAATTCCGAGAAGCAAGAAGAGCAT |
| | GAAATGCAGCAGGTGTATCTGCGACGATGATAACAACAACAATGCA |
| | GTGGCTCACAGGCAGCAGACCTCAAGCAGCTGCTGTTCAGAAGATG |
| | AGTACTCCAATGAGCTGATGGGAACGACGTCGTGCTCGAGCTCCAA |
| | GGGCGGACGGGCTCCCAATCCGAATGCGAAAACACGTGCGAGTAGG |
| | GGTTCAGCAACCGATCCACAGAGCCTCTATGCAAGGAAAAGAAGAG |
| | AGAGAATCAACGAGAGGCTCAAAATCTTGCAGACCCTCGTCCCAAA |
| | TGGAACAAAGGTTGACATCAGCACAATGCTGGAAGAGGCCGTTGAG |
| | TATGTGAAGTTCTTGCAACTCCAGATTAAGCTGTTGAGCTCCGATGA |
| | TTTGTGGATGTATGCTCCGATTGCGTACAACGGGATGGACATTGGAC |
| | TGGATTTGAGACCTCAATCCTCATGA |
| SmbHLH63 | ATGGATGCTTGCCTTCCTGATTGGAACACTGATGTTGGAGGTGAATT |
| | CCTTCTCCCACTTCAGAGAAAACCTCTTGGATTAGAAAATGAGTTAA |
| | TTGAATTGTTGTGGCAAAATGGGGAGATTGTGTTGCATAGTCAAACC |
| | AATAGGAAACAAACAAAGCAAATTAGGGATCCAAATCAATGTGGTT |
| | TGATTCAAGATGATGATGAAGCAGTTTCATGGATTGATTGCCCCATTG |
| | ATGAATCCTTTGAGAGGGATTTCTGTGCTAATTTCTTGTCTGAGATAC |
| | CTCCTCCAAACTTGGATGAATCGAGCAAGGGCGAAACGGAGTTCAA |
| | ATCCGGTGCATCGGAGGTTATCCCCCAAGGCTTTGCTCCTCCTCCTC |
| | CACCAAGGTTCGAGGCAGCTGAGGCCGTGGCTCGTCACCAGCCTCA |
| | GATGCCGGGGAGGAGCCGTGAGTGCTCTGGCATGACCCTCGGGTCG |
| | AGCCATTGTGCGAGCAACCAAGTGGACTTGAGTTGGGCTTCTAGCT |
| | GTGGGGTTGGCACCATGGCTGCAGGGGCGATAAACGAGGGCGTGGA |
| | GAGGGAGGCGCCCGGGCAGGCTAGGGAGTCTTGCTCCGGTGGCTCC |
| | GGTAGCAGCTTGTGGAAAACGAGCAGGCACAAGAGGAAGAGCCGT |
| | GACATGGAGGAATCAGAGTGCCCTAGTGATGCGACTGAATCGGAGT |
| | CGGCTGGAGGGAACAAGGCGAAGAATGGGACGGCCCGGAGGAGCC |
| | GTGTAGCTGAGGTGCATAACATGTCTGAGAGAAGACGAAGGGACCG |
| | GATCAACGAGAAGATGAAGGCGTTGCAGGAGCTCATCCCTCATGCC |
| | AACAAGTCGGACAAGGCGTCCATGTTAGACGAGGCGATCGAGTATA |

| | |
|---|---|
| | TGAAAGCCCTGCAGCTGCAGATTCAGTTGATGTGGATGGGGAGGGG<br>GATGGGGATGGCACCGGTGATGCTTCCGGGCATGCAGCACTACATGT<br>CGCGTGTAGGGATGGGAATCGGGCCAACGATGCTGCCCGGGATCCA<br>TAACCTAATGCGCCTATCGCGCTTGCCGCTCGTGGATCAAGCCATGA<br>GCGTGCCTCCGGCCGGCCACAACCCTATGTTGAATCCGGTTAATTAT<br>CCGAACCAGATGCAGAGCTCGAGTTTCCAAGAGCAGTACGCGAATT<br>ACGTGAACTTCCACTCGATGCAGAGTGCGAATTCGCCTCAGGTGTGT<br>GTGCTTCCCTACACTTGCCATGATCCCATGTATCTGCCCTGA |
| SmbHLH64 | ATGGGAAGAACTGCGAGCCACGTAGAAGACGAAGAAACTCACGAT<br>AGCTCTTCCCCTACAGACTACAAAAGAGTTGATCAGAAGGCAAACG<br>CTTTACGTTCCAAGCATTCAGAGACGGAGCAACGCAGAAGGAGCAA<br>AATCAATGAGAGGCAAGCTTTTAACTTCATTCTCTTTCTTTTTCTTTC<br>CCCTTGTTTAGGTTGTTTTCGTTCAGACAGATTTCAAATTTTGAGGG<br>ACCTGATACCCGAAAATGATCAAAAAAGGGATAAAGCTTCGTTTTTG<br>TTGGAGGTTATACAGTATATCCAGTTCTTACAGGAGAAGTTGCAGAT<br>GTATGAGGGATCTTGTCAAGGTTGGAGCTCAGAGTCCACAAAGTGT<br>TTGCCGCTGAGAATTAATTCTGGGCCAGATGAAAGCTTCGTTGACCA<br>GACCCAATTTGAAAGGAACTTTTATGGTCACGAAGACAATGTTCACC<br>CAGTCCTGCTAAGTAATGCGCAGAACTCAGTGGAATCGGACTTAACT<br>GGAGTTACCTTATATAAATCAGCAGTTAACCCACCCATGACAACACA<br>AGCCCTTGCTATGGCCATGCCTCTGCCAGCAGCTATATTTGAGAGTTT<br>GCCTGGCCAGCCCCACCAAGGATCTTTTACTGAAGCCGACCAATTG<br>AACTTTTGGCAGGGTAGATCATGTGCAGATGATTGTTCTGTTCCTGTC<br>TACTCTGCAAATGGGGAAGAACTGAAGAGTGAGAGTGGTGAAGCTA<br>GTATCTCAAATGTCTACTCTCAAGGGTTGTTGAATTCCTTAAAGTGTT<br>CACTACAATCGTCAGGGGTCGATTTGTCCCAAACCAACATTTCCGTG<br>CAGCTGGATGTTGGAAAACAAACTACTGGTGGAAATACCAATCCCG<br>TAATCTCTATCAAGGATCAAGAGAATGCCTCCGGTCTCTGCTATGGAT<br>CTTCTATAAATGTTGGTAAGAGTTATGAGCACCCTTCAAAAAGGTTG<br>AGAGGTGACGAGACCTAG |
| SmbHLH65 | ATGCAGCAAGAAAACAGTGGCGATGTATACATGGGCATGGGCGGCG<br>GCGATGGCGGATCAGCTCTAACTTTCCCCGACGTTTCACAAATCCTG<br>CCGTGGGCGCTCCCGCCGGTCCACGCCTTCAACCCGGTCCATTTCTC<br>GACCCGCGACCACGACCCGTTTCTCCTCTCCGCCGCCGCGGCGCAG<br>CCGCCCTATGGCGGCATGTTCAACAGGAGGCCGTCGGCGGGCTACG<br>AGTCGGAACAGCAGCTACGGCTGCTCTCGCAGTCGCTAGGGCAGGT<br>GGTGCACCACCACGGCTCCGGCGCCCCCTTCGGGCTCCACGCGGAG<br>CTGCAGAAGCTCTCCGCCCAAGAAATCATGGACGCCAAGGCGCTGG<br>CCGCGTCCAAGAGCCACAGCGAAGCGGAGAGGCGGCGTAGAGAGA<br>GAATCAACAATCACCTTGCTAAGCTAAGAAGCTTGCTCCCCAACACA<br>ACCAAAACGGACAAAGCTTCGTTGCTAGCCGAGGTAATTCAGCACG<br>TGAAGGAGCTGAAGCGCCAGACTTGTGTGATAGCGGAGACGAATCC<br>GGTTCCCACAGAGATGGACGAATTAACGGTGGATAATGCAAACGAT<br>GAAGAGGGGAAATGGGTGATCAAAGCTTCGATTTGCTGCGAGGATC |

GCCCGGATCTGTTGCCGGAGCTGATCAAGACGCTCAAGGCCTTGAA
GCTGAGGACGCTGAGGGCGGAGATCACCACTCTCGGAGGGAGAGT
GAGGAATGTGCTCTTCATCACCGGAGACGACGAAGAGGAAGAAGA
GGAAGAAGAAGCAGCCAATCACGACTGCATAACATCCATTCACGAA
GCGCTTAAAGCTGTGATGGAGAGGACCAACGCCGATGACGCGGCGG
GGAGTGTCAAGAGGCAAAGGACCAATCTCAATATTCTTGATCACTAC
AGGTCGTTTTGA

SmbHLH66 ATGGGGAAGCCTTGTCGAATTCCATCAAATGCAAGCACAAATCCAA
GAATTGAGAGGAAAATCATTGAGAAAAATAGAAGAAATAAAATGAA
GAATCTCTATTCCCAGCTCGTTTCTCACCTTCCACCTCAGCCCTCCTC
GCTAGTGGAAGGGGCGCCATTGCCGGATCAAATAGACCAAGTTGTG
GAGCATATAAAAAACATGAAGACGAAGCTGGAGAAGTTGAGGCAA
AAAAAGGATTTTTTATTGCAGAGAAATAAGCAGCCAATTAATAATAA
CTCATGCATTACAAATACACAAAATGAGGGCAAAAATTTGATATCATC
ACCTCTTGTTGAAGTTCAAGATATGGGGCCAAATCTTGATGTCGTCT
TAGCAGAAAATCTTCAAAGCTTCTCGAGTTTTCGCGATATCATAAGC
TTGGTTCATCAACATGGAGTTGAGATTGCAAGTGCAAGCTTTTCTAG
AGATGGCAACTCATCTATCCAAGTTCTCCACGATAAGGTCGGGCATC
CGAAACCGGGGTTTGATGGGGCGACGATCGCGAGAAAGATGAAGG
AGTTGGTATGCAAAGGGATGAATCCATCAAGCAGTGAAGTAGTTGA
ATCAGAAATGAATTTGTGGGATTATGGAATTGATTCTAATTGGGGATT
TGAAATTCCTGAGGTTTTGTTACCTGGCTTGCAGGAATTTATGGTCAC
AATGGAGAAATTTAAATGTGACAATAAAATTTCTGTGTAG

SmbHLH67 ATGAACAATCTAAACTCACTCATGAACCAGCACCAAATGTCGTCGCT
CCATGAAATTCACGATCACTCCCAATTCGATCCGCCGTCGTCGCACG
ACGAGTTCCTCCACCAAATGCTATCCACCACTTCCCCGGCCTCCGCC
TTCCCTTGGGGCGGCGACGACCAGCAGCATCAGATCAGCAGCGCTA
GAGCCATGCTGCTCCAGCAGCATCAGCTGCTGCTCTCCCGCAGCCTC
GCCGCCGCGAACGATCTCCGCTCTCCGACGGAAGCCGACCCCGGCT
TCTTCCCCCTCCCCCGAGACGATCGCGACGACGTCGTCGACGGCCC
CTCCTTCAAGCCTGCTATCGCGGAAAATGATGCATCCATTAAAGCTCT
ATTCAATGGCTTCACGGGTTCCCTAGGTCAAACAACAAATCAGGATC
AGCATTTCCCTCACTCTCAGACTGATAGTTTTGGAGTCTCGGCGTCT
GCGGCGGCGAGCCAGCCGTCGGCGGCTGGACAACCTCGGCAGGCG
GGTGAGGGCCAGGGCGAGGGCAGGCCACCGACCCTCACATTGAGG
AGGGAGCGAATTGCGGAGAGAATGAAGGCTTTACAGGAGCTTGTAC
CTAATGCTAACAGGACTGACAAAGCCTCAATGTTGGATGAGATCATC
GACTATGTCAAATTCCTACGGCTCCAAGTCAAAGTACTGAGCACGAG
CAGATTAGGCGGAGCTGCAGCTGTCGCCCCACTACTTGGTGATGCAT
CCTCTCAGGGAAAGAAGGGAGAGAAGGAAGGAATGGCGACGGCGG
AACAGAAGGTGGCGAAGCTTCTGGAAGAAGACATGGGGAAAGGGT
CTTTGCCTGATGCCTATTTCTCTGGCCACCTCCATCTCCTCCTCCTCC
ACCCAAACCTCTACTTAACCATCTCTGCTCCTTCAAATATGCTACTTT
CCATGCACTTCATTTCCTGCACATTTTTTATTACTATTCCCTTTTTGAA

GCCTAACTTCATCAAACATCGCAACTACTGA

SmbHLH68 ATGGAAAACTGCTATTGGCCGCATGATGCACCGCCATGGCTCCTATC
CCCCGCCGCCGCCGCCGACGACAGAGCCGCCTCCGCTTCTAGAAGC
CACAGCGAAGCAGAGAAGCGGCGCAGGGACCGAATCAATGCGCAG
CTCTCCACTCTCCGGAAGCTCATCCCCAAATCCGAAAAGATGGACA
AGGCAGCTCTGTTAGGGCACGTGGTGGATCACGTGAAGGAGCAGAG
GCAGAGAGCGAAGGAAGCGAGCAAGAGCAGCAGCAGCATGCCGAG
CGAGATCGACGAAGTGATCATCGACCAGCACCACGAATCGGAGCAC
ATCAAGGCATCAATCTGCTGCGACGATCGCCCCGAGCTCTTCGCGGA
GCTCAAGGCGGCGCTGAAGCCGTTAGAAGCCACCATTCTCGAAGCC
GAGGTCACGAGCTTGGGAGGCAGAATCAAGGCCAATTTCATCATATC
TGCTGCAATGGCGAACCAACACACGCTCAAAATGGCCTTGACCCGA
CTCCTCATCTCGTCCGGATCCGGATCGTCCGGTTACGGCACCCGGAG
CAAGAGGCAGAGGTTCTTCTATCCCACTTCGCGTTGA

SmbHLH69 ATGGATAGGTTTGATGATGAAAACGGAGGGATCCCATTTCCCCAATAT
TATGCAAGTGAATTTGGTTTGATCGATTTCATGGATGAAGCCAATGTG
GATCAGTTCATCGATCTCATTCGTGGAGAAAATATTCAAAATGATCAA
CCTATTTTCAACTTGTTCGATCAAGACTACCAATACTGCCGCGGAGAT
GAGAGCATTCATGTCGCCGCCGCCGTTGAGGACTGCCATTTCCTTCC
ACCACCGCCGCCGCAGGCGGAGCTCTTCGATTTCGACGCAGGCCAT
GAATTCGGGCTGCTGAATGCATGCGAGATTACCCAGGCGGAGGAGG
AGGAACTTCTCATGGATGAGGATGATCAGTCGTCGGGCTCAGGCAC
CACCACCACCGCGACCGGCTCGAGAAGCGCTTCCGGCAAGGCTGAC
AGATCGAAAACATTGGTGTCGGAGCGGCGGAGGAGAGGCCGCATG
AAGGAGAAGCTCTATGCCTTGCGCTCTTTGGTTCCTAACATCACTAA
GATGGATAAAGCTTCGATAGTGGGCGACGCGGTGTTGTACGTGCAA
GACCTGCAAATTCAGGCGAGGAAGATAAGGGCTGAGATCGCAGGTT
TCGAGGAGAAAACTCAACATAAAAAAACTACTCAAAATGGTAGCAA
ATCCAATTCAACAACCTGCTTCTACCCCACAACGAAGAAGATTTTTA
AGATGGAGGTGTCGCAAGTAGAGGATAGAGGGTTTTACGTGAGAAT
TGTGGCAAACGGAGGCCACGGCGTCGCCGCCCTCCTCTACCGTGCT
CTTGAATCTCTCACCACCTTCGACGTCCGGAGCTCCAGTTTGGCTGC
ATCTTCTGATCAGAATTATGTCTTCACCTTCACTTTGCATACGATGGA
AGGGGGAGTGGAGGTGAACTTGCAGAGTGTGAAGGTGTGGATTGCT
AGCGCTTTTCTCAACCAGGGATTTGATTTTCAGACATCATAA

SmbHLH70 ATGTCGGATCTTTACGGAAGCAATGAATCTGAAGACATGTCGTCTTT
CCTTCAAATTCTTCTGCAAAACTCGTCATCTTCTGCCGCCACCAGCG
ACAGCGCTGCGGCCGCCGGATGCTTGTTCGGCGGAGGAGGGGCGGT
GGCGGAGTCCTCCACTAGCATCAGTTTCTCCGATCCCAGCTGCTTCT
TCGCCAGAGAATCTGGTGGGGTGAGAGGCAAGAATCTGGCTTCCAC
TTGTGAGGGAGGTGATGCTTGTGATGTACCGGTGAATCCAGTCCCAC
CGCGTTCTTCAAAGAGAAGTAGAGCTGCTGAAGTCCACAATTTATCA
GAAAAGAGGAGGCGAAGTCGGATCAATGAGAAACTGAAAGCACTT
CAGAATCTGATTCCAAATTCTAACAAGACTGACAAGGCATCTATGTT

GGATGAAGCCATTGAATATCTGAAACAGCTGCAACTACAAGTTCAGA
AAGGGTTGAGTTTACATCCCGGATACTCCCTGGGGTCACTGCAGTCG
ATGCTAGCACCCTCAGGCGGTCTGGACATAGACGAAGGCGATCCAC
TGCTGCATGCAAATAGAGGGCTCTCTAGGGACCAAGACGTCTTTATG
CAAAGCTCTATTGCACCCACGAGCCACGGCCCCTCAACCCTGCCAA
CGCTCATGCCAAACACTACCAGTGCTTCAATGCTCCCTAGTTATGCA
CCACCGTTGCAGAACCGTTATGGACTGCTCAACCATATGGCCTCCAC
AAAGGACATTTGCAGGGATGACACGTTGTCTCGGTTGCAGCTTGATA
TAAGCTGCTCGGGCAACAATTCGTCGCCGGGGGTGTCCTCATAG

SmbHLH71 ATGTCAAGTAGTAGCAGGGAAAGAAGTTCAAGAATTACAGAAGATG
AGATCAATGACCTCATCTTGAAGCTGCAGCCGTTGTTGCCAGATTCA
AGGTCTAATACAAGGGTTTCAGCATCAATGATTTTAAAGGAGACATG
CAATTACCTCAAGAAATTGCACAAGGAGGTGGATGATCTAAGTGAG
AGACTGTCTCAACTTCTGGCTTCTGGAAGTTTCGATGCAGATGCTAT
TAGGGCACTCCTCCGACGTTGA

SmbHLH72 ATGCCTCTTTCAGAGTTTTTGAGAATGGCTAGAGGAAAACTTGAATC
TGGCCATCAGAAACCGGCTCCGGCCGATATCTCATCTAGGCCTGGGA
ATGAACTAGTTGAACTAGTTTGGGAGAATGGCCAGGTTGTGATGCAA
GGCCAGTCCAGTAGGGCTACAAGAAGCCCTACTCTTAACAATTTATT
GTCGAACACGTCTAAAGCTCGTGATGCTGCTACCACTGCAAGGTATG
GGAAATTTGGTGGAGTTGATTCTATCATGAATGATATGGTGCCAGTGG
TACCCTCAGGGGACATCGATTTAGGTCAAGATGATGAGATCGCTCCG
TGGTTAAGTTATCCCATTGATGATGCTCTAGGGCAAGATTATGCTTCC
GAGATCTTACCCCATATATCTAGTGTCACGGCTAATGGATCGCCTGCA
CAGAATAGCTTTGTATCAGCGGACAAGAGAAGTAGTTGCGAACAGG
CCGTTAATAATTTGCAGACTGGGGCTAGTAATGTGAAGGTCCCTTCT
AGATCTTCTTCTAAAGATCGTCTTTTTGGTTCATGGCTGCCTCAACAG
CATCGCCAGACATCAGACGCCTTAGGATCTGGTGTCACTGATAATTC
GAGTGACCATCTGGATTCTGTATTCGGTAATCCAGGTCAAAGTAGAG
ATACAGTTAATGGCTCAGCTAGTACGGTGATGGATAGGCGTAGTATTC
CACCACCTGCTAATTGTTCGAATTTCCTGAACTTCTCCCATTTCTCAA
GACCAGCTGCTCTTGCCAAAGGAAGCCTCTCAAACTCTGATGGGAT
TCCTATGTCAGTATCATCAGTTGTGGAGAGGGTTGAAACTAAGGATA
AAGCCAATTGTAGTAATCCAGTCAAATCGATACGCATCGAGCAAGTT
AAAAGCATGCCAAAAGACAACGACTCCCATGGTTCTTGTACGCTTC
CCGTGGTAGGTTCTAGGGAGCAAGTGATTAAGGAACCTCTGGAAAG
AGCTGATAATTTATTCAAAGAAACTTCCATCAAGAACGACAAGCCTC
TGATTCTAAGTAACAACGAAAGTTCAGCAAAAGGAGCCCCTGATGG
TGAGAGGATTGTTGAGCCTATGGTTGCTTCTTCTTCTGTGGGCTCTG
GAAACAGTGCTGATAGAGTTTCTTGCGAACAAACACAACATTCTAA
AAGAAAATTCTGCGATATTGAGGAGTCCGAATGCCGGAGTGATGATG
TTGAAACTGAATCTGTTGATGCCAAGAAAGCAACTTGTCTACGAGG
ATCTAAGAGAAGCCGAGCTGCAGAAGTGCATAATCTTTCTGAAAGG
AGACGGAGAGATAGGATCAATGAGAAGATGCGTGCTTTACAAGAAC

TCATACCAAATTGCAATAAGGTGGATAAAGCTTCAATGCTCGATGAA
GCCATTGAATATCTAAAGACCCTCCAACTACAAGTACAGATTATGTCC
ATGAGTTCAGGGTTATGTATGCCTCCTCCGATGATGTTTCCGACAGGA
ATGCAACACATGCATCCTGCTCATGTTCCACATTTCCAACCAATGGGT
GTTGGAGTGGGTATGGGTATGGCATTTGGGATGGGTATGCCGGACAT
GAATGGTGGATCCTCTGGCTGTCCTATGTATCCCGTTCCTCCCCTACA
AGTACCGCACTTTTCTTCTCCAATGCTGGGGCTCGCCAATTTCCAGC
GGATGCCTGGCCATAACCATCCAGTATTTGGGCATCCTGGTCAAGCA
TTTCCCAATCCAGTCACAAAACCACCGTTCGTTCCTTTAGCTCCGCG
CCCTCCTGTAACTTCTGCCATGGGATCAGGTGCTTTGAGGAATGGAA
GTAATGGTGAAATTCTTAGTACATCTCAAATTATGAAGTCCGGGGATC
CAGTGACGACCACGAATTCACAGTCAATGTGCAGTGCGGAAGCTAG
GAGCTCAGTCCACAACAAATCCAATCAGCCTACAAAAGAAGTAGTA
GATCAATGTGCTGCTGTGCAAGATAACGAACGGGCCACAGACGCGG
GATCTTCTGCAGCTCATTTGTCTGCTACCACTACGGACATTAACAAA
GAACCAGGAGGCGTGGAAGTGTTGGCCGAATGGCATTCCCCTGTTG
ACGACGTCTTCCTCTGCTCGCCTGGTAGGACATGA

SmbHLH73 ATGGAGGCTGTTGAGGCATTTCTTGATGGAGAATGGGAGTCATTGAG
CAAACTGTTTTCTTGTGAGGATTCAGATGCTGTGATGCATTGCAACA
GCGACAACTTGTTTCCATACTCTTGGAATAATGGAGGTGAATTTGAT
GCGAATTTGTATAATAATAATGTTGATCATGGTAGCTTTCATTCTGTTT
CACAAGAAAGCAGCAGCAGTGATCAGAATGAGAGGTCCCTTTTGGA
TCACTCATTCCCTGATGATCTCATGGAGGAAATCCTCCATTTGAAAGC
ACAGATGCTGTGCAATGATGACTTGGACAATGCTGATGATTCTTGCA
ACCTCAAGAGGAAACAAGCTCACAATTCATCACAACCATCCAAGAA
GAGGCCTCGCGTGGCGAAGAAGGCCAAGAGGGCTGCACAAGCACA
AGCACAAGCCACCAACAATGATGAAGAAATGAACAATGGTGGAGG
AGGAGGAGCAACTGTTAATGCTGTTGGCCAGAGCTCAAGCATCTGC
AGCTCTGAGGGGGAAGAATCCGAGTCGAAGGGCTCCACCTCCATGC
CCAAGAACAAGGCTACCAAGACCCCAGCAACTGATGCCCAAAGCCT
TTATGCAAGGAAAAGAAGAGAAAGGATCAATGAGAGATTGAGAATC
TTGCAGAATCTTGTTCCTAATGGAACAAAGGTTGATATTAGCACAAT
GCTAGAAGAGGCAGTCCAATACGTGAAGTTTTTGCAGCTTCAGATCA
AGTTGCTAAGCTCTGATGATAAGTGGATGTATGCCCCAATTGCTTACA
ATGGGATGGACATGGGCCTTTATCAGAGGATTGCTCCAAATCTATGA

SmbHLH74 ATGCAAAATCAACACGTCCATTCCTTCACCGAGAACTGCTGGTGCGA
TGTAATCGATTACAGCAGCCTCCTGGACGACGACGGCGCGCCGCCG
CCGGCCGTCGTTTCCGAGAGTCCGCCGCTGCCGTCGGAAATTGTTTG
TATGTCAATTGATGCCACACACAGTGAAGTTACAACACTTGAGGAAA
GAGGCTCCTCAAGAAAGAGGGGACGTTCTGATCAAAATGGTGGGAT
AGGAGCAAAAGCATGTCGTGAGAGAGAGCGCAGAGGAAAATTGAA
TGAAAGATTCTTAGAACTCTCCGCCATTTTGGGACGCGAAAGACCTT
TTAAAACCGAAAAACTGGCCATGCTTAGTGATGCCATCAGACTCCTA
AACCAGCTCAAAACTGAATCTATGGACTACATGGAGATGAACACAA

GGCTCCTCGAAGAGATCAAAATTTTAAAGGCTGAGAAGAATGAGCT
TCGCGAAGAGAAAGCAAAGCTAAAGGCAGATAAAGAAAGGATACA
GCAGCAGTTGGAAACCATGAATATTCCTTCAATTGGGTTCACGGCAT
CACATCCACCTATGTACGAGGCTGAGGTCAAGAAGGTTCCGATGCTT
CCCAGCTATGGATTTGTTCCGATGTGGCAGTATTTGCCGCCATCTGTC
CGAGACACGACTATTGATCATGAACTCAGGCCTCCTGCTGCTTGA

SmbHLH75 ATGGAGATTTCAGGCTTCAGAAACTTAGCTGATTTGGTTAATCAACA
TGTGAACGATGCTTTACCTGTAGATTTCCAGTCTTCATTTTATGAAGG
TTTGAGTCACAACTTTGAGCAAGCATGTGAATATGACGACAGGCCAT
TGAAGCAGTTCAGGACGAGTTCACACACGCCTGCAACCACTCTTCA
GTTTGACGTTATTCCAACTTGTGATGGAGTAGTTTGGATGAACCCCA
AAGATGAAGCGTTGGTCGCGTCTAAGCCAATCCCTTTCCACCAGCAT
CACAGTCACAACAAGCTTGCGGACCATTCTTTAGCAGAGAGAAAGA
GGAGAGAGAAGATCGGCAAGAGGTTTGCAGCCTTGTCTGCTCTAGT
CCCTGGACTCAAGAAGATGGACAAGGCTTCTATTCTGGAAGATGCG
ATTGAGTACATGAAGCTGCTGGAGGAGAAAGTAAAGGGGCTCGAGG
AGAAAGCTAGGAAGAGAAGCATGGAATCTGCAGTGGTGTTCACCAT
CAACAAAGACGACGAAGACGAAGATGACTTCCCTATAATTAGGGCA
AGATTCTATGATAAAGAGATGCTCATTAGTATTCACTGTAAGAGAGG
GAGAAGGGTGTTTGAGAAAATTGTTGGTGAAATTGAGAATCTGCAA
TTTTCTATTGTTAACAGCAGCCTCATGACTTATGGAGACTCTTCTCTC
AGCATAACACTAATAGCCCTAGCCCAGCAGAAAAATGACGAAGAAG
ACAGCATGAGCATGGAGCAGCTTGTCAGGAATTTACGAGGTCTTCTC
AAAATAATCTTGTGA

SmbHLH76 ATGGGTGAAGAGGTTGAGCGGGAGGGGTTGTGGGATGACGACCAAT
CATGGGAATTTCCAAAATTAGCAACTGTCGAAGAAGATTGCGGCATA
AAGTCGGCGGACTCTGACGGGCAGGTGGCGGTCAAGGATAATGGAT
CGCCGCCAACACCGCCAGAGAAGGGCAAGAAGAGGAGCGCCGCCG
CAGGAGGAGAGTCGGAGCTGCATATTTTGACGGAGAGGGAGAGGA
GGAAGAAGATGAGAGACATGTTCTCCAATCTTCATGCCTTGCTCCCT
CACATCCCTCCAAAGGCGGACAAGTCGACAATAGTTGATGAAGCTG
TGAAGTACATAAAGAGAGTGGAGCAGAGTGTTTTGGAACTGGAGAA
ACGAAAGCAATCCTTAATGACGCAAAACCAGGATTTGAAGACATGG
AATTGTGGAAACGTAGTCCTCAACGTGTGTGGGGCAGATGCTCACAT
CAGCGTGTGCAGCATAAGAAAGCCCGGCCTGCTCGCCGCCCTCTGC
TTTACGATGGAGAGGAACAATTTGGAGGTGGTTTCTGCTCAGGTCTC
ATCATGCATCTACATCATCCATGCCCGGCCGGCATATGGAGGGTCAGA
CCAGTTCCAGCAGGCATTCGCAATTGAAGAAAATTTCAAGCAGGCA
GCAGCAGAAATAACGATGTGGCTTAATTCCTAA

SmbHLH77 ATGTTAGCGATGGGTGAAGAGCTTGGGGGTGAGGGTTTGTTGTGGG
ACGATGACCAATCATGGGAATTTCCAAAATTAGCAAAGTCGGCGGA
CATCAGCAACTTAGGCGGCCAAAGGATAAAGGAAGCGCCGCCACCG
CCAGAGAAGGGCAAGAAGAGGAGCGCCGCCGCAAGAGGAGAGTC
GGAGCTGCATATTTTGACGGAGAGGGAGAGGAGGAAGAAGATGAG

AGACATGTTCTCCAATCTTCATGCCTTGCTCCCTCACATCCCTCCAAA
GAGAAAAGAGATGAGTGAGGAGAGGGAGACATTGGAGGGTGTAAC
CCCCGGGGACGAAGTGCGGAGTGATTGCCAAAGAGGCACAGACAA
GGAACGGCTCCGATTAAGACTTCCAAGCGGAGGGGCACAGGAATGA

SmbHLH78 ATGCAGCCTATCAGCGGAGAATTTGGCGCCATGAACAATCTCAACGC
GCTGCTTAACCAGCACCAAATCTCATCCCTGCACGAGCTCCACAGTC
ACGGCCAACTGCCGCAGATGCTGCAGTCTGTTTCGCAGTTCGATCCG
GTCGCGTCGCACGACGATTTTCTCGAGCAGATGCTCTCCTCCGTGCC
GTCCTCCGCCGCCTACCCATGGGAGGACGACCACTCACAACAGCAG
ATGGAGGAGCATTCCGCCGCCGTTCTGGCGTCTAAGCTGAGGCAGC
ACCAGATTAGCGGCGGCGCTGCTAAGGCGTTGATGTTTCAGCAGCA
ATTGTTGCTCTCCAGAGGCCTTGCCGCCGGTAATGGCGGTCTCTGCT
CCCCCACCGGATCGGATGGAGCTCTTCTTCCCATGGCGCAAGGCCAT
CATAACGACGGTGTTGATGGAAATGGTTTCAAGTCGATACAGGCAAA
TGATTTATCCGTTAAAGCTTTATTCAACGGATTCACTGGATCCCTTGG
TCAAACCTCAAATCAATCGCAGCATTTCCACCATCCTCAGGACCAGA
ATTATGGTGCTGCCGGAACAGCTACACCGTCAACGGCAGCGCAGGC
GATGAATCAACCTGCAGTGAGCGGTTCAAGCGGCGGAGGGGCTTCT
GGACAGCCGCGGCAGAGGGTGAGGGCCAGGAGAGGACAGGCCACT
GACCCTCACAGCATCGCGGAAAGATTACGGAGGGAGAGGATTGCGG
AAAGAATGAAGGCACTGCAGGAGCTTGTACCCAACGCAAATAAGAC
CGACAAAGCCTCGATGCTGGATGAGATAATCGACTATGTCAAATTCC
TACAGCTCCAAGTCAAAGTTCTAAGCATGAGCAGATTGGGCGGTGC
TTCAGCTGTTGCGCCATTAGTTGCTGATATATCTTCTGAGGGGAGGA
GCAGTAACGGGACACAGACGGCGTCATCATCAAACAACAACGAGG
GGATGACGGTGACGGAGCACCAGGTGGCTAAGCTGATGGAGGAGG
ACATGGGCTCCGCCATGCAGTATCTTCAAGGAAAGGGGCTGTGTCTC
ATGCCCATTTCCCTCGCCACGGCCATCTCCACCGCCACTTCTCACTCT
AGGAACCCCCTCGCCGCCGCCAACAGCCTCAAAATCAGCGGCATCT
CCGACGCTCGAGGCCCCACTTCGCCCAGCCTGTCTGTTTTGACCGTC
CAGTCCGCCACCGTGGGGGGTAACCTTAACAGAGAACCGTCCCTTA
AAGACGTTTCCAAGCCATAG

SmbHLH79 ATGGAAATTGCAGGATTTATTGATGAAGAATGGGAATCATTGAGCCA
AATCTTCTCAAGCGTTGAAAATGTGGATTTGTTGCAGCATTATGGCG
AACCTTCATTTCTCTCTCAGGAAAGCAGCAACGAAGCCCTTTTCTTC
AACATTGATGAAGATCATCATCAGCCGCCGCCTTTGGACACCAACTC
ATTTGATGAGACCATGATCATCAACATGCTGCCCGATCACGATGTAAT
AATGGAAGAGTTTCTACGTTCAAATCGGCCTTTGGATTCTTGCCAGA
ATAAGAGGAAGTCTGAGGAGCATCAACATGATAAATCTCCCAAGAA
AAAACCAAGAAATCAGCAGCAGAAAAATACAAAAAAGGGGAAGAA
ACAAGTCCAAGATAAGAATATAACTGAAGAAGAAATAAATAGTGGA
GGAAATAGACAGAGCTGCAGCAGCTACAGCTCTGAAGATGATGACT
CAAATGCTTTGATTAATGAAAAAATCAACAACTCCAAAGCCAGAGC
TAGTAGGGGATCTGCAACTGATCCACAAAGCCTCTATGCAAGAAGA

AGAAGAGAGAGAATCAACGAGAGATTGAAGATCTTGCAGAATCTTG
TTCCCAATGGAACAAAGGTTGACATTAGCACAATGCTGGAGGAAGC
TGTGCAATATGTGAAGTTTTTGCAGCTTCAAATTAAGTTGCTGAGCT
CTGATAATATGTGGATGTATGCCCCACTTGCTTACAACGGAATGGACC
TCGGCATTTATGATAATATTTGTCCAAATCTGAGGCAATAG

SmbHLH80 ATGATGAATGGTTATGCACATCACGTGGATTCACTCGATGAGAATTGC
TGGACCGACTTAATCGATTACAGCAGCCTTTTGGACGACGCCGTCCC
GGCCGCCGGCCTTTATTGGAACCCTCAGCCACCGGCGCAAAAGTGG
TTAGAGCGAGGTTTGAGCTCATGCACAGCTTTTATGTTTTTAAACTCT
GAGCTGCTTATAGGTGTTCCTAGAGCTCATAAGGCGGTTAATTATCAC
TCAGTTGTTAAGATTCCAGACTATAGTCATGGTGAAATTATTCTGTGT
GGAACTACATCACATGTCAGTGACTCTGCAGAAGAGCGAGAAAGCC
GAAAAAAGAGGGGACGCAGCGATCAATGTGGTGGAACAGGAAACA
AAGCATGCCGTGAGAGAATGAGGAGAGAAAAATTGAATGACAGGTT
TACAGAATTGTCTGATACTCTAGAACCTGGAAGACCTGCAAAAACG
GACAAACTGGCCATACTAGGTGATGCCATCAGAGTTCTAAACCAGCT
AAAAACTGAATCGGAGGAGTATAAAGAGATGAACGAGAAGCTTTTA
GAAGAGATCAAAACTTTGAAGGCGGAGAAAAACGAACTTCGTGAA
GAGAAGCTGGTGCTCAAGACAGATAAAGAGAGGATGGAGCAGCAA
CTGAAAAGCATGACCGTTCCACCTACCGGGTTCATGCCTGCACATCC
ACCGGTATACCCTGCTGCCCCGAACAAGATGCCAATGTTCCCCGGCT
ACAGTCTCGTCCCGATGTGGCAGTATCTACCACCGGCTGCTCGTGAT
ACGTCTCAAGATCATGAGCTCAGGCCTCCTGCTGCTTGA

SmbHLH81 ATGCTCAAACCTGGAGGTTTTGAGGGCAGGCTCCAACTCTCTCTCTG
GTTCATACCTCTCAATCTATACACCTTGACAACATTGCAAGAACTCTC
TCTACCTAACAACCAACTCTCCGGCGCAATCGACGGCGCCATCGTCA
ACCTCTCAAACCTCATCATCTTAGAGCTCCATGTGAACCAACTATGG
GGTGAGCTACCTTCTAACATAGGCTTGCTTTCCAAATTGCAGCAACT
GCAACTCCACTCCAACAACCTCAACGGCACAATACCTCCCTCTTTAA
CAGACTGCACCAACCTCACCACTCTCCTCCTCCGCAACAACCTCTTC
TCCGGCCAAATCGCCGCTCTCGATTTCTCCAAACTGCAACGCTTACA
AGCCCTCGACCTCGGCAACAACACCTTGGTCGGAGGCATCCCGGAG
AGCCTCTGCCTGTGCAAGTCCGCCACCGCGATCCGCCTCGCCTTCAA
CCAGCTCACCGGAGAAGTACCTCCCTGCATGGCCTCCCTCCACTCTC
TCATACACCTCTCTCTCTCCGACAACCTCCTCTCCAACGTCGTCGGA
GCCCTGACCACTCTCCGGCACTGCGAGAATCTCGCGGTGCTCTTCCT
CTCGAGGTGCTTCCACGACGAGAGGATGCCCGACGACGACGACCTG
CTGCATCTCGACAGCTTCAAGAATCTGCAGATTCTAACCCTAGGAGG
ATGCAAACTCAGAGGCGAGATCCCGACGTGGATTTCGAAGCTGAGG
AAGGTCAAAGTTCTCAACCTCTCCTTCAACAAGATCTCCGGCCCGAT
TCCGGCGTGGCTGGGATCAATGCCGAGCTTGTACGTGCTCAACTTGA
CGCAGAATTTCTTGTCGGGAGAAATCCCACGTGAGATCGGGCGGCT
GCCGGCGTTGATCTCCGACAACGGCAGCACGGACTTGAGCCACCTG
GCGCTGCCGTTCTTGTTCGACAAGCTTCAATACAACCGCCTCTTCAA

CCTGCCGCGCGGCCTCAAGCTGGGGAACAACAGCCTGAGCGGCAG
CATTCCGGCGGAGATTGGGCAGCTGAAGCTGCTCATCATATTGGATC
TCAGCAGCAACGAGCTGAGCGGCGGCATACCTGAGCAGCTCTCGAA
TCTGACCAACTTGGAGAGGTTGGACATGTCGGGGAACCATCTGTCG
GGGGAGATACCGGCGGCGCTGACGAAGCTGCATTTCTTGTCGGCGT
TCAGCGTGGCCAACAATGATCTTCAGGGAGAGATTCCCAAAGGGGG
TCAGTTTGAGACGTTTGGGGCTGCTGCGTTTGAAGGGAATCCGAAA
CTGATAAACGGGAGCCACGTGGTTGCGGTGGCGCCGCCGCGTGTGG
AGGAGGAGGAGGAAGAAGAAGAGGGGTTGTGGCATAATACTCGCTT
ACCTTTTGGGTTGGGATACTTTGTAGGAGTAATCCCTTCTTCATCAAT
GGCGTTGAGCTACTACACCTTTCAGCAGCCAGACTACTCCGGCGATG
ATCCAGAGCTCCAATCTCTGTTGAACCCGGACGGCGACTCCTTCTGC
AACTCTCTTCTCTGCGACGATCTCACTTATCATGCTAATGGACTTCCC
TTGGATTTGGATAATCTCACAACTGCAACCCAACCCCCACCCTTTCT
ATTGCCGCACCAACAACAACAATCCCAGGCGCCCTTGTTCCACTTCC
CCAACCGCCACAAGCTCTACGATTACTCGCTGCCGGAATTTGTCTTG
CCGCCGCCGTTGCCTCAGTTCCCTGCCGCGGATTTCAGCACCAGGAT
CTGTGAGAACGCCAAGAACGAAACGAGCTTATCCGCGCAGAGCATC
GCAGCCAGGCAGAGGCGGCGGAAGATCACAGTGAAGACGCAGGAG
CTCGGGAAGCTGGTTCCCGGCGGGCATAGGATGAACACGGCGGAGA
TGCTGCAATCCGCCTACAACTACGTCAAGTTCTTGCAGGCGCAAGTT
GCTCTCCTTGAATTCCTTGGTTCGCATCATCAGGAGGTACCATTCGA
AGGTGAAGAAGAGCTCCAGAATCTTCTAGAATCTCCTTTGATTCAAG
AAAAGCTATACTCCACTCAACATTGCTTGCTTCCAAACAAGTTGGCG
GAACAAGTTCCATTACTCAAATCCAATCCACATTTGCTGGAAAAGGA
TCATTGA

SmbHLH82 ATGGCGAATCTCTACGATACTTCTACTAACAATTATTACTCTCCTCAA
GAGCCAGACGAGATTTCTCTTTTCCTCCAGCAAATTCTGCTCCGTTC
CAATTCTTCGCCGTCGCATTCATTGCCGCGGCAGCACTCTCATCTCGT
ACCGACCTCCGATGGCATCGCGGCGGAGCAGCACGTGGCAATTGGT
CAGATCTCCGCGTTGAACTCGTGCTCCACTTGCGGCTTCGCGAATGT
ATCGCTCGAAAACTTGGAGAATGAAACGGATGATTGTGACTATGAG
AGCGAGGAGGCTGTTGAAGCTGCGGTGGGAGAGACGGTGGCGAAG
GCGCGGTGCTCTACTAAGAGGACTAGAGCAGCTGAGGTGCATAATCT
CTCTGAAAAGAGGAGGAGGAGCAGGATTAACGAGAAAATGAAGGC
ATTGCAAAATCTAATTCCAAATTCAAACAAGACTGACAAGGCTTCGA
TGCTCGACGAAGCTATCGAGTATCTCAAGCAGCTCCAGCTCCAAGTA
CAGATGTTAACAATGAGAAATGGGTTAAACCTTTACCCTATTTGCCTG
CCGGGTATGCTGCAGTCCAATCAGGTTTCTCAAATGAGGGCGGATGT
CTATGCCACAGATAAATACTCAAATGTAAATATGATGAAAGAAGTTAG
ACTGGACCAGAATATCTCGGCTGATTCTCTTCTCGGCCTACCTGAAA
ACTGTGTTAAGCAGGAGCACGTGTCCGATTTCTCAAGTATGATTGGT
TCAAAAACTTCAATGGAAGTCGAATCATCCATGCAGCAGCATCACTT
CGGAGCATTTCAATTCTCCAGATCATCATTTAAAAAAACTGAGAAGG

| | |
|---|---|
| | TGTTGCCACGCCAGCCTTTTATGGAAGATTGCCCTCAAACTAGCTCT<br>GCTGGAGCAAAAACGACATCTTCAATCCGTTTGGACACACAAACGT<br>CCGATTTGAAGGCTAATATTTTGGAAGGATGCGTTGCTGGAGCAGAC<br>ATTCTACTTACAAACTATGTTAGTAACCCAGTCTTCTCATCTCATGAC<br>AGTTGA |
| SmbHLH83 | ATGGATCAACTCAATCCCAACTCGCTTTTTCTGCCCGCGCCTGATGC<br>GTGCGTCCCGCCGACTGGCCCGACTCGCCCCAATCCCGATTGTAGAA<br>TGGAACCAGAAACAAAGGACACTGTAAGTGCTCGGAGAGTTCAAA<br>AAGCTGATCGTGAGAAGTTGAGGAGGGATCGTCTCAATGAACAATT<br>CATGGAGCTGGGGAACGCCCTCGATCCCGATAGACCTAAAAATGAC<br>AAAGCCTCTATTCTTTCGGATACGATTCAAATGCTCAAGGATTTGACT<br>GCTCAGGTTGAAAAACTAAAATCTGAGTATGCTGCACTCACTGATGA<br>GTCACGTGAGCTGACCCAGGAAAAGAATGATCTCAGAGAAGAAAA<br>AGCATCTCTTAAATCCGATATTGAGAACCTTAATGGTCAGTACCTACA<br>GAGAGTGAGGGCCATGTATCCATGGGCTGGAATGGATCATTCTGTTG<br>TCATGCATCCCTCATCTTATCCCTTTCCAATGCCCGTCCCTGTACCAA<br>CTGGACCTATTCCCCTGCATCCATCCTTGCAGCCTTATCCTTTCTATGG<br>GAACCAAAACCCTGCAGTTGTTCCTAATCCTTGTTCTACGTATGTTCC<br>TTATATGACCCATAATCCAATGATTGAACAACAATCAACACAACATGT<br>CTCACAAGTCATGCAGCAAGGTACCAGGCCTCATGCTTCAACCAAA<br>CAAGACCCTAGAAATAAATCATCAGATGTGGAGAGTAGAATAGAGA<br>AGAGTGATGACTCAAATGACATCACAACAGATCTCGAGCTTAAAAC<br>ACCTGGATCAACATCCGAACAGGATTCATCCTCGAAGCAAAGAAGA<br>GCAAAAAGGGTTGTGAGGAAGGAAACAAGCGTTACAGATGGAACT<br>TCTTCTAGTGGATGTTCATCATCTCACAGCATGCAGGCTATCTCATCT<br>AACAGTATTGCCGGTGGCAAAAGAACAGACGACTGA |
| SmbHLH84 | ATGGATCCGCCTTTGGTAAATGAGGCGTCGTTTACCGCCGCCGCGAA<br>CCCCAATTCGTACAGTTTGGCGGGGCTGCTTTCCTTCTCCGGCAGCG<br>GCGGGCTGGGGCTCAGAATGGGGAACTTGGGGCGCAGCGGCGGAG<br>GAGACGCGTCTCTCGAGGAATCGACGGTTACGGAGCAGAGCGGGA<br>GCGGGAATGGCGGCGGCCCGAAGCGGAGGAGGGATGTTGGGAGCT<br>CTTTTGATGACGACTCCTCCAAAATTGTCTCCACCAGCAGTGCTAAT<br>CAGGACGTGGTATATACCCAAAAATTATTTGATTCCGGAGATCAATTG<br>AAAACGGAAACAGAAACACATTCGGGGAGCAGCAAGCCTGTTGAG<br>GAGAAAAGTAAACCCGAACCGCCTAAAGACTATATTCATGTTAGAGC<br>AAGACGGGGTCAAGCTACTGATAGCCACAGTTTAGCTGAAAGAGCT<br>CGGAGGGAAAAGATCAGCGAAAGGATGAAAATCCTGCAAGATTTGG<br>TACCGGGATGTAATAAGGTTATCGGAAAAGCTCTTGTTCTTGATGAA<br>ATAATTAATTACATCCAGTCCCTACAGCGTCAAGTTGAGTTTTTGTCG<br>ATGAAGCTTGAAGCAGTTAACTCGAGGTTGAGTCCCACATTAGAAG<br>GGTTTCCCTCCAAAGATCTAGCAGGACCTGCATTTGATGCCAACGGG<br>ATGATATATGGACCACAAACACCGGGAGTGTACGCCAGAGGATCGC<br>AGGCGGAGTGGCTTCACATGCAGGTTGGAGGTGGTTTTGAAAGATC<br>AACATGA |

SmbHLH85 ATGTTGGCTATTTCTCCTCAAATGTGTTCATATGGGTGGGTTATGGAT
GATCCCATGATGAGCCATGAACAAGAGAATTTGAGCTACTTTTCTAG
AGAGAGAACAGAGACGACCTCAGGTTCCATTGATCCCCACTCCTCC
CCCTCCTCCAAAATCCCACTCGATTTCAACGACGATTTCTTGAATGG
AGATGGAGATAAGAAGGTGAAGAAACTCAACCACAATGCAAGCGA
GCGTGATCGCCGCAAGAAAATGAACACTCTGTATGCCAACCTTCGCT
CTCTCCTCCCTCCTGAGGATCACTCCAAAAAGCTGAGCATTCCAGCC
ACCATCTCGAGAGTGCTGAAATACATACCTGAGCTGCAGAGAGAAG
TGGAGAGACTGATGCAGCAGAAGGAGAGATTCATCTCCAAGATTTC
AATGGCGGCCGAAAACTCATCTCTTGAATTCAAGAATCACACAGAG
AAACCAACTCAACGAAGCTCGAATTCAGCAGCTTCTGCAACTAGAA
TCAACGACGGCCAAGTCGTTATTCAGATCTCCATGCCCAAATCAGAA
AAGGGTTCCATTTCTGAAGCAATCTCGAGATTGGAGGAAGAAGGTT
TTCTCATGGTGAATGCTTCTTGTTTCGAATCATTTGAAGGGAGGCTTT
TCTACAATCTGCATTTTCAGGCACAAGAAGGTCAAGTGATAGATGCT
GAGAAGTTGAAGGAGAAAGTTTGGCCTTTGATGTAG

SmbHLH86 ATGGATCCACAAGGCCCCGCCATGATGAATGAAGGCGGGGTTTATAA
CTTCGCTGAGATCTGGCCGGCTTTTCAGATGAATGCCACTGCGACGT
CGTATGGATTGGGCTTGGATCCGATGGTGATGGACCAGAGATCGAAT
CACAGTCCACCAAATCACAATTCTAGGAAGCGCCGCGAAGATGATG
ACTGTGTCAAGGGAGGAGCTTCTACTAGCAATAGCAATGGCAACAG
CAATAATACTACTAATAATAATAACAACAATGTTATGAGTGAAGGAGA
TAATAAGAGATTGAAGGCGGTTGGATCCAACCATGCTCGTGAAACTA
AAACAGAAGGGGAAGTGAATTCGACTAAGGCGTCAGAAGCACTTG
CAAAACCAGCTGATCCATCAAAGCAAGACTACATCCACGTACGAGC
AAGAAGAGGTCAAGCTACTGATAGCCACAGTTTAGCTGAAAGGGCT
AGGAGAGAAAAGATTAGTGAGAGGATGAGAATTCTTCAAGATTTAG
TTCCTGGTTGTAATAAAGTTATAGGCAAAGCATTAGTGCTCGATGAG
ATTATAAACTATATCCAATCATTACAACGCCAGGTTGAGTTTCTGTCG
ATGAAGCTTGAAGCAGTCAATGCAAGAGTGAGCATTGATGGATACC
CTTCAAAAGATTTCTCTCAGCAAACGTTTGATAGTTCTGGCATGGCA
TATGGTTCTCAAAGTACAAGAGAATACGGTAGGGGTTCTTCACCAGA
ATGGCTACACATGCAGATTGGTGGCGGTTTTGAACGATCATCGTGA

SmbHLH87 ATGGTTGAAGAAGCCGAGCATGACAGCAGCTTGTTGTGGAACGACG
ATCAATCATGGGCCTTTCCAGTTCTTCCGGTTCACGATAATGGCGGC
AAGCTACTCATAGACGGCGGCAAGATATTATTGTCGGAGGGCACCGC
CGTTTCTGAGCAAGCGGCGAATGACAAGGGGAAGAAGAGGAGTGG
TTGTGGCAGCGGCGAGTCTGATGATCACGATCACGATCACGATCACG
AGCTGCATATATGGACGGAGAGAGAGAGGAGGAAGAAGATGAGGA
ATATGTTTGCCACCCTCCATTCTTTGATTCCCCACCTCCATCCCAGGG
CAGACAAGTCGAGCATAGTTGATGAAGCAGTGATCCACATCAAGAA
TATGCAGCAAACTCTGGAAGATCTTGAAAAGCAGAAGGAGGAAAA
GCTGAAAGGTGGGCAATCCCGAGAGGCATTTCTAGCTGAGCAGGGA
TCCACAAGCCAAAGCCAGCTGCAACCCAATGCGAATGCGAATGCCA

ACGGTTTCTTCTTCAAAACATGGACTTCTCCGAATGTAGTGATGAAT
GTATGTGGGAATGATGCCCACTTCAATATTGTTTGCAGCCCTATAAAG
CCCGGCCTAATGACTTATGTTGTGTTTCTCATGGACAAGTACAACCTC
CACCTCGTCTCTGCTCACGTCGCCTCCGATCCCACCGCGCGTACCTA
CATGCTTCATACCCGCGCAAATGGAATTCCGCAGCAGTTCCCTGAGG
CGGCATCATTTCTTGTGGAGGAAACGTACAAGCAAACTGCAACTGA
GCTCATGCTTTGGATTAATTCCTAA

SmbHLH88 ATGGAAAGTGTGGGAACAATGTGTGAAGGGGAATGGAGCTCTAGCT
TCAACGGGATGTGTTCCGACGAGGCGGATTTCATGGCACAACTGCT
GGGGAGCTGCTCCGTCCCGGTCTCATCTTTCTGGGCTGCAAACGAC
GAGACAAACATGTTTTCCTTTTCACTAGGAAATAACAGCTTCATTTTC
CCCTCTTTGAGTAGCAACACCACCACTTTGATGCCAATGGATTACTC
TCTCATGGATGCTAATGCCACGACTCTTATGGAAGGCGAAGACGACT
TCCTAAACCACGACCACAGCGACGCCTCCGACAACAAACTTCTTCA
AGAATCATCACTCATCACCACCCACATATCCAAGAAAAGGCCTCAAA
TTCCTACAGGATCCCAAAGAAGCAAGAGGTTCAAGAAATGTGCAGA
TGAAAATGATGACGCCAACAATGCTAATGGTAGTAATGTGGTGCTTC
ACAGACAGAGCTCATCAAGTGGGTGCTGCTCAGAAGACGAATCCAA
CGGGGTGAAGTCGAGCTCGAGCTCGAGGAGAAGTGGAGCTCAGGA
CGTGAAGGGCAAGGCAAGGGCTAGTAGGGGATCAGCTACTGATCCA
CAGAGCTTATATGCTAGGAAAAGAAGAGAGAGAATCAATGAGAGAT
TGAGAATCTTGCAGAACTTAGTGCCAAATGGAACTAAGGTTGATATT
AGTACAATGCTGGAAGAGGCTGTCCAATATGTCAAATTCTTGCAGCT
GCAGATTAAGCTTTTGAGCTCAGATGATCTATGGATGTATTCTCCTATT
GCCTACAATGGAATGGATTTAGGGCTTGATTTGAAGATTCCCTCTCCA
AAACCACAATTATGA

SmbHLH89 ATGGAAAGAAAGAAAGAGGAACTTATTTGGGGGACGTCCCAAAAA
GGAAAAAGAGGAACTTATTTCAGGGACGGAGGGAGTGGCTACCTTA
AAACGCATGACTTCTTGCAACCATTGGAACGGGTGGGGAAGCATGT
AACAACGACGAAAGAAGATATTAAAGCTGGGGTGGCTGCTGTTGAT
AGGCCTCCAGCGCCAGCTCCTCCAGCCTCAGTGGAGCATCTTCTTCC
TGGTGGCATTGGGACGTACAGCATCTCTTATTTCAATCAACTGGTGTT
AAAGCCTGAGGGGAGCTTGTTTACTGCTCAGCCAACCAGGAATGAT
GAAAACTCAAACTGCAGTTCTTACTCGGGAGGTAGTTTCACTCTGTG
GGATGAATCTGCGGTTAAAAAGGGAAAGACAGGGAAGGAGAATATT
GCCACAGAAAGACATATTCAGCGAGGTATCACTTTCTCATCTTCTCA
GTCAGTGACTTTTTATTCCATTTTTTTTTCCAAAGACGATGATGATGA
TGAAGAAGAAGAAGAGTTCACCATCAAGAAAGAGCCATCACCCCA
CTCAAAAGGTAATTTGTCTGTGAAAGTTGAAGCAAAGACTATAGATC
AGAAGCCTAATACACCACGGTCTAAGCACTCAGCTACAGAGCAACG
AAGGAGGAGCAAGATTAATGACAGGCATAGGCTGAGAGAGATCATT
CCTAATAGTGAGCAGAAGAGAGATAAGGCATCGTTCCTCTTAGAGGT
TATCGAGTACATTCAGTTTTTACAAGAGAAAGTAAACAGATATGACA
GTTCCTATAACATCTGGAATCATGAGCCAGCCAAAATGATGCAATGG

GATTCTGCTCCAGCGTCAGTATTTGGCAAGAAGTTCGATGAGAGCAA
AGCTGCTGTCTCCCCCAGCCTACCAATCAGCGGGCAAAACCTAGTA
GACTCAGATTTGAGCACTGCTACTACTTTGAGAGAAAGAGTGGACA
CAAGCATTCCAGCTTCCCCGATTTCACCAAAACAGGCATCCGATCTG
GAGAAAGCTATGACATGGCCTCCATCTCAGCTTCGATCATGTACCAC
TGACACTAAAATGACAGAACATGAACTGACCATTGAAAGTGGCACC
ATCAACATCTCTAGTGTCTATTCCCAAGGGCTGTTGAATACTCTTACA
CAAGCGCTACAGAGCTCTGGAGTGGATCTATCCCAGGCCAGCATCTC
AGTACAAATCGATCTGGGGAAGAAAGCAAACGGCGTCACACATTCT
TCTACCTTTATCTCCAAGGAGGATGAAGTTGACACCACCTTACCGCG
TTCTGCAACGGCTAGCACGGGGAAGGAATTCGGGCGTGCTGCGAAG
AAACTAAGGACTAGCTGA

SmbHLH90 ATGCTATCAAGAGTGAATAGCATGGTCTGGATGGACGGCACCAAAG
GAGACACCGACGAGCACACATCTTCCTGGCCCCAAAACGACGACGC
CGACAACAACAACAAGGACCAAATGGAGATCGGCGCCCTCTCCACC
TTCAAATCCATGCTCGAAGCCGAAAACGAGGACTGGTACATCACCA
GCACCGGCGCCACCTCCGCTGCTGCGCCCATGCACGACATCTCATTC
TCCCCGACCTTCACCGAAGCCGCCGTCAACAACCAGCTGCTGCTCC
AGCCGATCGATTCCTCCGCCTCATGCTCCCCGACATCCGCCTTCAAC
AACAACCTCGACGCCGCATCGCAGGTGAACTACTTCCTGCAGCCCA
AGCCCTTGATCAACCATCCAATCCAAAACAACGCTTTAGATGCGAGC
TTTGATTTGGGATGCGATGGTGGATATCTCGAGAGTGCCCTAAATAG
GGGCGGAGGGATTCTCAATTGCGGATTTCCGATGGGGAACATAGGGT
TTGGGCTCGGAGAGGGATCGGCGAGCTCGTTGTTCAACAGATCGAA
GATTCTGAAGCCGCTGGATCACAATTTCGGATCAATGGGATCTCAGC
CGACGCTGTTCCAGAAGAGGGCGGCTTTGAGGAAGAATCTCGGGAG
TAATTTGGGGTGCTTGAGCTTGGATAGGAGCCAGATTTATGAATCGG
AGATGAAGAGGAAAGGCAGCAGTGGGGATGACGTGGAGGATTTGA
GCATCGATGGCTCCAGTTTCAACTATGATTCCGATGATCAATTCTTGG
ACAGCAGTGGCGCTGTGAGCAAGGGGGAGTCCGAGAGTGTTAGGA
ATGGCGACGGCAAGGGGAAGAAGAAGGGGCTGCCGGCGAAGAATT
TGATGGCGGAGCGCCGCCGCAGGAAGAAGCTCAATGACAGGCTCTA
CATGTTGAGGTCAGTGGTGCCAAAGATTAGTAAGATGGACAGGGCT
TCAATACTAGGGGATGCGATAGATTACTTGAAGGAGCTCCTGCAGAA
GATCAACGACCTCCACAACGAACTGGAGGCCATCCCGGGGGCGGGG
GCGTCCACCCCCACCCAAAACACGAGCTTCTACCCGCTGACGCCTA
CAACTCCTGGAATCCCTGCCCGGATCAAGGAAGAGCTCTGCCCGAG
CGCCTTTGCAAGCCCGTTGTCCAGCCCCACCGGACAACCGGCTAGG
GTTGAGGTGAGACTAAGGGAAGGCAGAGCAGTTAACATCCACATGT
TCTGTGGCCGGAAACCTGGGATTCTGCTCTCCACTTTGAGGGCACTC
GACAATCTTGGGCTTGACATACAGCAAGCTGTCATCAGCTGCTTCAA
TGGCTTCGCACTCGATATTTTCCGAGCTGAGCAATGGGGAGAAGGG
CAAGAGCTGAACCCTGACCAAATCAAGGCAGTGCTCCTCGATTCAG
CTGGCTTCCATGGCATGCACTGA

SmbHLH91 ATGCTAGTGCAAAGCTTCATAGAGAGGCTTAGGCCCCTTGTGGGGAT
CAAGGGCTGGGATTACATCGTGCTGTGGAAGCTCAGCGATGATCGC
AGGTCGATTGAGTTGATGGATTGCTGCTGTGCCGGAGGAGACAACG
CCGACGAGCTCGGCTTCGAGGCTTCTTCTTCTACCCTTCCTTGTAGA
GATGTCATGTATCCTCATCCAAGGCTCAAGTCATGTGATTTACTTGAC
CTCATTCCTTCATCCATGGTGTTGGATTCTGGAGTTCATGCACAAACC
TTGTGCTCAAACCAAGCTAGGTGGCTCAACTACTCACACAGCTCAG
ATTCAAGCCTCTCATCACAAGATGATATTGGAACTCGAGTTTTGATCC
CGTTGTCCGTGGGATTAGTGGAGTTGTTCGTGAATAATCAGGTGGGT
GAAGACGAAGGTGTGGTGGATCTGATCAGGGTGCAATGCAGCATCT
TCCTGGAGCATCAGACAACGATGAGTAACTCCGGGACCACAGACTC
CTTCTCCTCCGGTCAAAAGGATCCAATGAGCCTCTTCCAGCAGCCGG
TTTCGTCTCCCAATGAGAAGATGATGGAGTTGCCTAACGACATCTCC
ATTGACAGGATCCATCTCTCCAACGGCATATTTCTGGAGGGGGAGTG
CATCTTTACTCCGTCTATGGAGAATGCGTTTCACGATCAGATGCAGG
AGGAGTGTGAGAACAATCGATCCGATGACTCGGATCCTAATGATCAA
GATGAAGATGATCCAAAATACAGGAGGAGGACAGGGAAAGGGCCC
CAGTCAAAGAACCTTGAGGCCGAGAGGAAGAGGAGGAAGAAGCTC
AATGACAGGCTCTACGCCCTTCGTGCATTGGTGCCCAATATTTCTAAG
TTGGATAGAGCTTCTATTCTTGGAGATGCAATCGAGTATGTGAAGGA
GCTGCAAAAGCAGGCGGACGACCTGAAACTCGAGCTGGAACAGCA
GTCTGACGACGAGGGGAGCGCAAGAAGGACAGACGAGTTCCCACA
GATTCAACAAAAACGCGGCCCTAAACGCGAGCAGGAGAGTCTTGCT
AGTGGCTATCACAACAACAACAACGCCTCCAAACAGAAGCATGAGA
GCGATCAAAAAGTGCAGCAAATGGAGCCCCAAGTGGAGGTGTACCA
AGTGGATGGGAAGGAGTTCTTCGTGAAGGTATTTTGTGAGCATAAAA
GTGGTGGATTTGTGAGATTGGTGGAGTCTCTCTGTGCAATGGGACTC
GAAGTATCTAATGTTAACACAACCAGACACACTTGCTTGGCTTCTAG
CATCTTCAAAGTCGAGAGGAAGAATGAGGAGACGGTGGAAGCTGAT
GATGTGAAGGAGTCGTTGCTGGAGCTGACGAGAAATCCGTCGGGAA
TGTGGGGTGGTCACGGCGGAGCTGCAGAATCGGAAACCAACAATGA
CGAGATGCACCACTCCTTGTGCACTTCCCACCATCATCAACACTAA

SmbHLH92 ATGCTTCCTATTTCGAGCGATGGGGTATCTGAGGATCCTTCCATATTTT
TGGAGCTAGAAGATCTGCTTGCCGATTGTCCCATTCCAGATGGCAAT
CAAAATACTGTGAAAAAGTCGTCGGCCAGCAGCCAAGTTGAAGAG
AACAATCAAGACTCTAAGAAGACTGCTCATAGATTTACTGAAAGGC
AGAGAAGGCAAGAAATGTCAGCCCTTTATGCTTCACTCAGGTCTCTT
CTTCCCCTCCAATATGTCAAGGGGAAGCGCGCTGTATCTGATCACAT
GCACCAGGCTGTGAATTATGTTAACGATATGAAGAAGAACATTGAAC
AACTGCAGAAAAGGAGAGACAAATTGAGGAATATTACCACTTCTGC
TGATCCAACCACTCCTCGTTGTGTGAAGATAAACCTTTTTAAAGATG
GGATGGAGATCTTGATTACTCATAGCCTCAGCAACAAGAGTTTCCCC
CTTTCAAAGGTGCTTGCATATTTGCTTGATAGACAGCTCAACGTAGTT
CACTGCGTTTCTACCACCACAGCACCCTCAACTTTCCTCCAAACAAT

| | |
|---|---|
| | TCATATCGAGCTTAATGATTCGAGTAGCGTTAATCTACCTGAGCTACA<br>AGAGCGGCTGGACAATTTGATAAGATTTGCTTATACGGCAGCTGACG<br>ACAGCTAG |
| SmbHLH93 | ATGTTCTCTTTCCAGCCATGTGATGAGCTGCCTTTCCCTATCCAAACC<br>ACTCCTCAAAACTATCAGATTTCGGAAGGAAATTTAATTAGTTTCAG<br>TGCGACCACCACCAAAGAAGAGCAGCTGATCGGAGCTTCAAACTCC<br>CGATCGCCGAAGCAAAAGGGTCAGATCAGGTATGGATCATATAATAG<br>GAGGGTTTTGCACAGAGATATTGAAAGGAAGAGGAGGGAAGAAAT<br>GTCCAAGCTTTATGTTTCTCTGCGCACCCTTCTTCCCATGGAGCACA<br>ACAAGGGGAAAAGCTCTGTAAGCGATCAAATGGAGGAGGCTACGA<br>AATACATAAAACAGATGCATGAGAAAATCGAAAAATTGCGAAGTCA<br>AAGAGACAAGCTCAAGAAATTACACAATTCCTCTTCTTCGTCTTGTG<br>GCACAAACGTAGAAAGTTCATATGATAGAAATGGATTGCATAACAGT<br>GTGGCGGTGCAGCGGGTTATGGATGGATTGGAGATCCTCATCACCAA<br>CAAAAGCCCTCAGTTTGAGCTTTCTCACGTGCTTGCACAACTCTCTG<br>AAAGAGAGCTAGATGTGGTTAGCTGTGTTTCCACAACAACAAACGG<br>TGGATTTCTCCACAAAATCCTTATTAAGGCCAGCAATAGGACGTGCC<br>TTGATGTGTGGGGGCTGCACGATAGATTAAGTGACACTATCAAGTAA |
| SmbHLH94 | ATGACTATGGAAGCACTTTCTTCAAGTGAGCTCTTGAATTTCATCATG<br>TATGACACAAACGTCTCCGCCCCATTGAGCTGCAACAACAATGACTC<br>CTCCGAAGCCAGCCCTCGCGAGATTGGGAGCTGCTCCTCCAGGCCG<br>CGGCCGACCCACCCGCCGCCGCCGGCCGAAACGGCCAGCGTAGCG<br>CTGCAGGGCCGCAAGAAGCGGCGGCGGAGGCCCAAGGTGTGCAAG<br>AACAAAGAGGAAGCCGAGACTCAGAGAATGACTCACATTGCCGTCG<br>AAAGAAACCGCCGAAAGCAAATGAATGAGCATCTCGCTGTCTTGCG<br>CTCTCTCATGCCCGACTCTTATGTTCAACGAGGTGACCAAGCCTCAA<br>TTGTTGGTGGTGCAATAGAATTTGTGAAGGAGCTAGAGCACATTCTG<br>CAGTCACTAGAAGCCAAGAAATTTGCATTATTGGAGCATGGCAATGT<br>GACAAACAAGGAGCATCATGATGATGAAGAAATTAGAAATGATGATA<br>GGAATTTAGGCAGCCCATTTGCTCAATTCTTTGCATACCCACAATTCA<br>GTTGCTCCCACAGCCAAGTTAGTAACAAATACACATCTCAGAGCAA<br>GGCAGCCATTGCTGACATCGAAGTTACCCTAATCGAAACCCACGCTA<br>ACATCCGAGTGCTGTCGCGGCGGAGGGTGCGGCAGCTCTCGAAGAT<br>GGTGGCCTCGTTCCAGTCCATCTTCCTGGCCATCCTCCACCTCAATGT<br>GACCACGCTCGACACCCTCGTGCTCTACTCCATCAGCGCTAAGGTGG<br>AAGAAGGTTGCCAACTCAATTCAGCAGACGACATAGCCGGAGCAGT<br>TCACCACATGCTCAGAATAATTGAGGAGGAAGCCATCCTAGGTTGTC<br>ACACCTAG |
| SmbHLH95 | ATGAGTTCCGCGACTCAAGCCCAGAGTAGAGCTCCGGATGCAGCCC<br>AGAGCCGAGCTACAGAGGCAACACAGAGCAGAGATCCAGTGCATG<br>TTCCCGTTGGTCCGGTGACGAGAGCTAGAGCCAAGACTTCGTGCGG<br>CTCCACTTCCTCCGAGAAGAGCAAGAAGAAGAGGGGAGTGGCCGC<br>CGTGAAGCTGTCGACGGACCCTCAGAGCGTGGCGGCGAGGCAGCG<br>GCGCCACCGCATCAGCGACAGGTTCAAGATTCTGCAGAGCCTGGTG |

CCCGGCGGCTCCAAGATGGACACGGTTTCGATGCTGGAGCAAGCCA
TACAGTACGTCAAGTTTCTCAAGACTCAGATTTGGCTGCAGCAGGCC
ATGATCAACTTAGTTGATGGCTTTGATTGTTACGCGGTGGATCGCTTG
CTGCCGGAGTGTGGCCAGTTGCCAGAATCTTGGTTTTCCGGTCAAG
AGAATTGGGCTTCTGATGCGTCCATGCACAGCTGA

SmbHLH96
ATGGCTGATTATCATCAATTGATCTTGAAAGAGTCGTTCGATCCCGAC
GTGGAGTTGATCAACAATCTCTCAGCCTCAGGGCTATTCCAAAATCA
TGATGATTTCAATTTCCACAACTCAATTTTACCAACTCATCATGAGTT
TGATGATCATAAACAAAGGAATAAGACGAAAGCCAGCACCCCTGCA
GAGAGCACTTGTTCATATCAATCGCATCATACTGCCAATCAGCAGCG
GCAGCGGCGTGGAGGAAGAGCAAAGAAAGAAGAAGAAAAAGGAG
GTGAAGTGATTCATGTAAGAGCCAAGAGAGGCCAAGCCACCGATAG
TCACAGTTTAGCTGAACGAGTTAGAAGAGGAAAAATTAATGAGCGA
TTACGGTGTTTGCGAGATATTGTTCCTGGATGCTACAAGACGATGGG
CATGGCAGTGATGTTGGACGAGATCATCAACTACGTGCAGTCGTTGC
AAAATCAAGTGGAGTTTCTATCGATGAAGCTGACAGCAGCTAGTTCC
TTTTACGACTTCAACTCAGACACAGATGCCATCGAGGCAATGCAGA
GAGCAAATGCAATTGAAGCAATCAAGGCTGGGAATGTAGCAAACCC
TTGTGCTCATCTACCCCACAATTTCGGCTACTATCCATGA

SmbHLH97
ATGTCGAGCAGAAGAGCAAGAGCTTCCAGACTTACAGAAGATGAGC
TTAACAACCTCATCTTCAAACTACACGCATCCTTGCCACATCATTCTA
ACTCAACCTCTAATAAAAAGACAAGTGCAGCAAAGATTTTGAAAAG
GACATGCAATTACATAAAGAAGCTGCAGAAGGAAGTGGATGATCTA
AGTGATGGACTTTCTCAAGCTTTGGCTTCTGGAGATATCAATGCCGC
TGATGCAGACACCATTACCACATTTTTACAGCTCTAA

SmbHLH98
ATGTTTCCTAATTCGAGCGATGGAATTGACGATGCTTCGATATTTTTG
GAGCAAGATAAATTACTGGAAGATCTGCTTGCAGATTGTCCCATTTT
AGATGGCAATGACAATCCTAAAAAGAGTGTCAAAAAGAGGCCCATT
GAAGCTGAAAATAACAGCAATGGCAGCAGCGAATCCAAGAAGACT
GCTCATAGATTTACTGAGAAGCAGAGAAGGCAAGAAATGGCAGCCC
TTTATGCTTCTCTCAGATCCCTTCTCCCTCTTGAATACATCAAGGGAA
AGCGCGCTATATCTGATCATATGCACCAGGCCGTGAATTACATCAACG
ATATGAAGAAGAAAATCGAAGAACTGAAACCAAGGAGAGAGGGTT
TGAGGAATGTAGTAGGAACTGCTGATGAAGCTGAGAGTCAAACCTC
AAATGCTACTGATTCCTCGCCTTACTGTGTTAAGATAAATGTATTCAC
AGATGGGGTTGAGATCTTGATTAGTAGTAGCCTCAATAAAGGATGTT
TCCCTCTTTCTATGGTGCTTGCCGATTTGCTTAATAGACAGCTCGATG
TAATCAACTGCGTTTCTTCAAGAGGCGACCAATGTTACCTCCACAAA
ATTCACATTCAGGTACAAACAAAGAGCTATCACATATAG

SmbHLH99
ATGTTTCCTATTTCGAGTGATGGGGTATCTGAGGATCCTTCCATATTTT
TGGAGCTAGAAGATCTGCTTGCCGATTGTCCCATTCCACAGGGCAAT
CAAAATACTGAGAAAAAGAGGCCCAGAAAATTTGCATCGACGAGAA
GCCAAGATGACTCAAAGAAGACTGCTCATAAATTTATTGAGAAGCA
GAGAAGGCAAGAAATGACAGCCCTTTATGCTTCACTCAGATCCCTTC

TTCCTCTCCAATATGTCAAGGGTAAGCGCGCTGTATCTGATCACATGC
ACCAGGCTGTGAATTATGTTAACGATATGAAGATGAACATTGAACAA
CTGCAGAAAAGGAGAGACAAATTGAGAAATATTACCACTTCTGCTG
CTGCTGATGATGATGGTGATCCAAACACCACCGTGAACGGCCATGAT
TGTGTGAAGATAACCCTTTTCAGAGATGGGATTGAGATCTTGATTACT
CATAGCCCCAATGACAACATTTTTCCCCTTTCAAAGGTGCTTGCAGA
TTTGCTTGATAGACAGCTCAACGTCGTTCACTGCGTTTCTACCACCA
CATCACACTCAACTTTCCTCCAAACAATTCATATTGAGGTGGCGACA
GAGAACGTGGCGGTGCCAGCGGGATTTGAGAGAGAGAGAGGCGCG
ACATCAGGCAGCGCGGCGCAGCCTCTCTTTCCCGGCGAAGAAGGCC
ACCGCCGACCGCCTCTCTTCCCTCTGAGCCCGAAACTCGGCGCCGC
CTCTCTCAAATCGCCGCCTCCATCTGAGCCCGAAACCCTAAGGTATC
GTCTCCCTCAAATCGCCGCCTCCCTCTGA

SmbHLH100 ATGAAATCTGGAAAAGGCCATCAAGAAGAGGAAGAGGAGGAGGAG
GAAGAGTTTGGTGCGAAGAAGGACGCCACATCATCCGCTAATAACA
GCAAAGATGGAAAGAACAGTGAGAAAGCTAATGCTATAAGGTCAAA
ACATTCAGTCACGGAGCAGCGCAGGAGGAGTAAGATCAATGAGAGA
TTTCAGATATTGAGAGAACTTATACCACATAGCGATCAGAAGAGAGA
CACTGCTTCGTTTTTGTTAGAGGTGATTCAGTATGTACAGTTTTTACA
AGAGAAGGTACAGAAGTATGAGGGGTCATATCAACCTTGGAATTCA
GAGCCCACAAAGCTGATGCCATGGAGAAACAGTCATTGGCGCGTTC
AGAATTTTGTTGGTCAGCCACAGACGACAAAAAATGGTCCTGGTCC
TGGTTCTTCTTTTCCTGGAAGATTTGATGAAAATAGTATAGGAGTCCC
CATGACCCTGCAACCGGGCCAGCCGAATCCTATTGAACCCGATCATG
TTATGGATGCTCAAGGTAGACCGATAGATCTACACACTGAACTTTCTA
ACAAATCAATGAGCATGCCATTGCCTATTCCAGTTTCCATGGGAAAT
GACGGCCCATTCTCCCACACGTTACATGGGCCACCACCATCAGAGGC
GCAGTCATCGGACTGTCCTAGCACTGCTGATGCACTTAACCTTCAAG
ATGAACTAACAATCGAGGGGGGCACAATCAGCATTTCCAGTATTTAC
TCTGAAGGATTATTGGCTGCGTTGACACAAGCACTTCAAAGCACGG
GCGTGGATCTCTCTCAAGCCAGCATCTCGGTCCAGATCAATCTTGGA
AAACGAGCAAATAGAGGAGCCACAGCATCGACTGCTAAGGATCATA
TAATTCCTACGCCATCCAGCAACCAACCAGTTGGGACATTTCACGAC
GCCACTAGTAGTGAAGACTTAGATGTAGCTCAAAAGAGGCGAAAGA
TTTGA

SmbHLH101 ATGGATCCGCGCGGGGTGGAGATGATGAGCGGCGGCGGCGGAGGA
GGAGATGTCTATAACTTCGCGGAGATCTGGCCGCGGTTTCAGATGAG
CGCCAATGCGGCGTCGTACGGCTTGGGGTTGGATCCGATGTTGACGG
ATCAGAGATCGAACGACGATTCGCGGAAGCGGCGGGATGCAGACGA
GCATTGCGCCAAGGGAGGGGGAGCTTCCACCAGCAGCAGCAATGGC
GGCAGCTGCAACAACAATAACAACGTCTCGGCAGTGGGTGAACTTA
AAGTGGAAGGGGAAGGGAATTCCGGCAAGGGGGCGGCCGCGGTGG
AGAGACGTTCGAAGCTAGACGAACTGCCTAAGCAAGACTACATCCA
TGTTCGAGCAAGACGAGGTCAAGCTACCGATAGTCATAGTTTAGCAG

| | |
|---|---|
| | AAAGAGCTAGGAGAGAAAAGATTAGTGAGAGGATGAAAATTCTTCA<br>GGATTTGGTTCCTGGTTGTAATAAGGTGATTGGCAAAGCTCTAGTGC<br>TTGATGAGATTATTAATTATATTCAATCTCTACAACGTCAGGTTGAGTT<br>CTTGTCAATGAAGCTTGAGACAGTAAATTCAAGAGGGGAAATCGAT<br>GAATATCCTTCAAAAGATTTTGCTCAGCAGACGTTTGAAGCTCCGGG<br>TTTGGCCTTTGGTTCTCAGAGCACGCGTGAGTACAGTAGGGGCTCGT<br>CACCGGAGTGGCTACACATGCAGATTGGCAGCGGCTTTGACCGGAC<br>ATCATGA |
| SmbHLH102 | ATGCTTCCTATTTCGAGCGATGGGGTATCTGAGGATCCTTCCATATTTT<br>TGGAGCTAGAAGATCTGCTTGCCGATTGTCCCATTCCAGATGGCAAT<br>CAAAATACTGTGAAAAAGTCGTCGGCCAGCAGCCAAGTTGAAGAG<br>AACAATCAAGACTCTAAGAAGACTGCTCATAGATTTACTGAAAGGC<br>AGAGAAGGCAAGAAATGTCTGGCCTTTATGCTTCACTCAGGTCTCTT<br>CTTCCCCTCCAATATGTCAAGGGGAAGCGCGCTGTATCTGATCACAT<br>GCACCAGGCTGTGAATTATGTTAACGATATGAAGAAGAACATTGAAC<br>AACTGCAGAAAAGGAGAGACAAATTGAGGAATATTACCACTTCTGC<br>TGATCCAACCACGAATGCCGGTGATTCGCCTCGTTGTGTGAAGATAA<br>ACCTTTTTAAAGATGGGATGGAGATCTTGATTACTCATAGCCTTAGCA<br>ACAAGAGTTTCCCCCTTTCAAAGGTGCTTGCATATTTGCTTGATAGA<br>CAGCTCAACGTAGTTCACTGCGTTTCTACCACCACAGCACCCTCAAC<br>TTTCCTCCAAACAATTCATATCGAGGTAATATCAATTTATTCTACTAGT<br>CTATTTTCTTTCTGCAATAGTAGATTTCACTATCATGAAATACAATAG |
| SmbHLH103 | ATGTTTCCCAATCATCACGAGTTAGGTTTCGGAACCCCAACCACTAC<br>TTCTCAAGGATTCCAAATCGGGCAGGATACGGATTACAATTTCTTGG<br>AAAATAATTTAATTAGTTTCTCAATCGGCACCAACGACACAGAAGTG<br>AGCAGAAGGAAAGATTTGGAGTTACATGGAAAAACGAAAAAGGAG<br>AGCAGCAGCAAGAAGGTTATGCATCGAGAGTTGGAGAGGAAGAGG<br>AGGCAAGAAATGTCACACCTTTATGCTTCTCTCAGCTCCCTTCTTCCT<br>CATCATCAGATCAAGGGAAAACACGGTGTTTGTGATCAAATACAAG<br>AAGCTGCGAGTCACATAAAGAAGATGGAGAAGAAGATTGAAGAATT<br>GAAAATTCGCAGAAACAAGTTGAACGGAGACGTGGAAACTTCGAAT<br>GGCATATATAATAATGTGATAATAAACATAGTCGCGGATGGGATGGAG<br>ATTATCATTTCCCATAGCAGCGAAAGATCTGATTTAGGGCTATCACGA<br>GTCCTTGCTGAGTTACAGTGGAGAGAGTTTGATGTGATTAGCAGTGT<br>CTCGACCAGAATGAAGGACAGATTTGTCCACAAAATCCAGGTTGAG<br>GCCCGGGATATCTCAAACCTTGATGTGTTGATGCTGCGAGATAGATT<br>GACTGAGGCCATCAAGTAA |
| SmbHLH104 | ATGGCGTTAAGCTACTACTCCAACTGGACCTCTTTTCAGCAGCCAGA<br>CTACTCCGGCGATGATCCAGAGCTCCAATCTCTGCTGAACCCGGACG<br>ACTATTTCGCCGACTCCTTCTGCAACTCTCTTCTCTGCGACGATCTCA<br>CTTATCATGCTAATGGACTTCCCTTGGAATTGGATAATCTCACAACTG<br>CAACCCAACCCCCACCCTTTCTTTTGCCGCACCAACAACCCTTGTTC<br>CACTTCCCCAAACGCCACAAGCTCTACGATTACTCGCTGCCGGAATT<br>TGTCTTGCCGCCGCCGTTGCCTCAGTTCCCTGCCGCGGATTTCAGCT |

GTGAGACCGCCAAGAACGAAACGAGCTTATCCGCGCAGAGCATCGC
AGCCAGGCAGAGGCGGCGGAAGATCACAGTGAAGACGCAGGAACT
CGGGAAGCTGGTTCCCGGCGGGCAGAGGATGAACACGGCGGAGAT
GCTGCAATCCGCCTACAACTACATCAAGTTCTTGCAGGCGCAAGTTG
CTCTCCTTGAATTCCTTGGTTCGCATCATCAGGAGGTACCATTCGAA
GGTGAAGAAGAGCTCCAGAATCTTCTAGAATCTCCTTTGATTCAAGA
AAAGCTATACTCCACTCAACATTGCTTGCTTCCAAACAAGTTGGCGG
AACAAGTTCCATTACTCAAATCCAATCCACATTTGCTGGAAAAGGAT
CATTGA

SmbHLH105 ATGGATCCACAAGGCCCCGCCATGATGAATGAAGGCGGGGTTTATAA
CTTCGCTGAGATCTGGCCGGCTTTTCAGATGAATGCCACTGCGACGT
CGTATGGATTGGGCTTGGATCCGATGGTGATGGACCAGAGATCGAAT
CACAGTCCACCAAATCACAATTCTAGGAAGCGCCGCGAAGATGATG
ACTGTGTCAAGGGAGGAGCTTCTACTAGCAATAGCAATGGCAACAG
CAATAATACTACTAATAATAATAACAACAATGTTATGCACTTGCAAAA
ACCAGCTGATCCATCAAAGCAAGACTACATCCACGTACGAGCAAGA
AGAGGTCAAGCTACTGATAGCCACAGTTTAGCTGAAAGGGCTAGGA
GAGAAAAGATTAGTGAGAGGATGAGAATTCTTCAAGATTTAGTTCCT
GGTTGTAATAAAGTAAGTATCTGCAAAGCATTAGTGCTCGATGAGAT
TATAAACTATATCCAATCATTACAACGCCAGGTTGAGTTTCTGTCGAT
GAAGCTTGAAGCAGTCAATGCAAGAGTGAGCATTGATGGATACCCT
TCAAAAGATTTCTCTCAGCAAACGTTTGATAGTTCTGGCATGGCATAT
GGTTCTCAAAGTACAAGAGAATACGGTAGGGGTTCTTCACCAGAAT
GGCTACACATGCAGATTGGTGGCGGTTTTGAACGATCATCGTGA

SmbHLH106 ATGCTTATTGCGGCTGTTGGGCATCTTTATGCCTTCCCTTACAAAGAG
TATGCTGGTGCAAATATAGGCTCAAATCATGGCTGGAGTGATTCAGA
ACTCTTTATAATATGTATGCTTGGTGTACTTGAGGGCATCTGCCGACT
TTTGCTGGTGGATAGACATGGTTCTGCTCCTTTATGGCAAACATGTTC
TATTGGAAACTGTAATACATGCTTAGCTTGGCCTATCTGCATGAGTGA
AATGATGCATAGATCGAACGACGATTCGCGGAAGCGGCGGGATGGA
GATGAGCATTGCGCCAAGGGAGGAGGAGCTTCCACCAGCAGCAAC
AATGGCGGCAGCTGCAACACAATATACGTCTCGAATGAACGAAGGA
ATGAAGGTGATAGTAAACGGATGAAGGCAGTGGGTGAACTTAAAGT
GGAAGGGGAAGGGAATTCCGGCAAGGGGGCGGCCGCGGTGGAGAG
ACGCTCGAAGCTAGACGAACTGCCGAAGCAAGACTACATCCATGTT
CGAGCAAGACGAGGTCAAGCTACCGATAGTCATAGTTTAGCAGAAA
GAGCTAGGAGAGAAAAGATTAGTGAGAGGATGAAAATTCTTCAGGA
TTTGGTTCCTGGTTGTAATAAGGTCCGAGCTGCTATCAAGGTCAAGG
CATCAGTCATCATCTGTTTCACTTCTTCCAGGAAGAGCTGCTAG

SmbHLH107 ATGCCTCTGTATGAATTTTTGAGATTGGCTATAGAAAAGCTTGAATCT
AGCAAGCAGAAGCCATGTTCAGTTGATCCCTCTTCAAAAACAGAAA
ATGAGTTATTTGCTAGGGATGGTAGAAGCCCCACCCATCTCAGTTTG
CAGCCTGATACCCCAAGTGTCTGTGATGATGCCACCTTAGGAAATTG
TACGACTGGAAGGGCGGGAAAATTTGGTGGTGTCGAGTATATCTTCA

| | |
|---|---|
| | ATGATATTTCACCAGAGTTGGATTCAAGTCGAAAGGATGAAACGGCT<br>CCTTGGATAAGTTATCCCAAAGATGATACTCTGCAAGATTACTCTTCT<br>TTTAGTGCTCATCCACTGCTTCCATCAGCTCCTTCCTTACTAAATTGC<br>CAAGATGCTAGATTTTGGAATAGTGAGGAGGGTAGAGATTTGGTGGA<br>GAGTCGTAATACAGAATTACCGGTTGAGGAGATAGTTTCAGCTCCAA<br>GATGCAGTGCTACTGTCAAACGCGTGCTTATAGACCAGTTGAATATC<br>ACGGAGAAGGATGTAGACTTACATGTTCCGGCAGCAGTAAACTCTA<br>GAGAATTGTTTGTTGATGAAAGGACCAAGAATATCAGCCTAGATACA<br>TTCATGAAAACTGACAAAAGTCTAATTCAAAGTAACACCATCGAATC<br>AACTAGACCTCTAGATGGGAAGACAACAGTTGAGCCTATTGTTGCTT<br>CTTCTTTTGTAAGCTCCGGAAACAGTGGTGATAGGAATTTCAATGAA<br>CAGAAGCGTTACACCAAAGGAAAATCTCATCACAATGAGGACTCTG<br>AATGCCAAAGTGATGAACTACAAAATGAATCTGTTGGTTTGAAAAA<br>ACTGAGTCCAGCTAAAGGAGGTAGTGGATCGAAGAGAAGGCGGGC<br>AACAGAAATGCATAATCTTTCAGAAAGGAGGCGAAGAGATAGGATC<br>AATCAGAAAATGCATGCTTTACAAGAACTTATACCCAACTGCAATAA<br>GGCGGATAAGGCTTCTATGCTTGATGAAGCCATTGAATATGTAAAGA<br>ATCTTAAACATCAAGTACAGATTATGTCGATGGGATATGGATCATGCG<br>TGGCACCATTTATGTATTATGCAGCTATACCTCATTCTTCATACGCCGG<br>AGTGAGAATGGGAAACATGAGTGGGGGTTCTCCTCAATGTCCTGTTT<br>TCCATGCACATTTTCCTACATCGGTCGTAAATTGCCCAGAATTTCCCT<br>CTCCGATTCATGGACCACGCTTTTTTCCTCTAACTAAATCTCAACAAA<br>CTTTAGTAACAAATCACGATCAAGTGCCATGTATGATTACGAACAAG<br>ATACTCAGCAGCAAGCCGAGAAATGAAGGCCCAAATCAGTCCCCTA<br>CAGCACGTGAGAGTAATTGA |
| SmbHLH108 | ATGGAAGAACAAGATCGCAATGAATATTTGCAGTTTTCGCCACTGGT<br>AATGGATTGGATAGCAAGTAATGCATCTGGTTCACTTAGTAACTTTGA<br>CTCCGACCAGCACTATATCAAGGCAGCAGGGAGTGGGCCTTGTCAG<br>TGGATGCATTGCCAGCCAACACCTCAAAACTATGTTCAGTTTCTTTC<br>AGAGACAGGGCACACTGAAATCCAGAAACAACTGTCATATGATGTT<br>TCAGCACAATTTCCTACTTTGGGACTACCAAATCCTTCTCAATTGGA<br>AGAATACCTGAGGCTGCATAATGGTGGAGATTTTGTGACCTCAGTCA<br>CAGGCTTGGAACAACTTGGTGTAGAGGAACAGAAGTTGAATGAAG<br>AGTTTAATCATGCATTTGAAACTTATTCACAATCATGTGCTGCTGCGT<br>ACAAAGATCCTTCTAAACTCAATAGGAAAAGATCAAGAGAGAGAGC<br>TTATGCTACTGATCGTATTCGCAAATTGAGGATATCACGTTGGCTAGA<br>TGCTTTACAAGAATTGGTTCCTTCTCCAGAAAGGGGTGGTCAAGCA<br>GCCTTGCTGGATGGGGTCATTGACTATATCAAGTATTTGCAATATCAA<br>ATGAAGGATCTATGTCGAAACCGATTGGGAGGTGGATCAACTTCTAA<br>TTCTGTTATTTTCCTTGAGGAACATGGGCATTACTTTGTTCAGGATCA<br>GATGCTCAATGGTCCTCTGGAAGAGATGATAGGGAAGTTGATTAGTG<br>CTTATCCTTCAGCAGCTACTGAACTGCTGCAGAGTAGAGGTCTCATC<br>GTGTTGCCTATGACTTTTGCAGAAGAATTACTTGAATCTACGGACAT<br>GCTGGATATGCAAGGTTGA |

SmbHLH109 ATGCTATCAAGAGTGAATAGCATGGTCTGGATGGACGGCACCAAAG
GAGACACCGACGAGCACACATCTTCCTGGCCCCAAAACGACGACGC
CGACAACAACAACAAGGACCAAATGGAGATCGGCGCCCTCTCCACC
TTCAAATCCATGCTCGAAGCCGAAAACGAGGACTGGTACATCACCA
GCACCGGCGCCACCTCCGCTGCTGCGCCCATGCACGACATCTCATTC
TCCCCGACCTTCACCGAAGCCGCCGTCAACAACCAGCTGCTGCTCC
AGCCGATCGATTCCTCCGCCTCATGCTCCCCGACATCCGCCTTCAAC
AACAACCTCGACGCCGCATCGCAGGTGAACTACTTCCTGCAGCCCA
AGCCCTTGATCAACCATCCAATCCAAAACAACGCTTTAGATGCGAGC
TTTGATTTGGGATGCGATGGTGGATATCTCGAGAGTGCCCTAAATAG
GGGCGGAGGGATTCTCAATTGCGGATTTCCGATGGGGAACATAGGGT
TTGGGCTCGGAGAGGGATCGGCGAGCTCGTTGTTCAACAGATCGAA
GATTCTGAAGCCGCTGGATCACAATTTCGGATCAATGGGATCTCAGC
CGACGCTGTTCCAGAAGAGGGCGGCTTTGAGGAAGAATCTCGGGAG
TAATTTGGGGTGCTTGAGCTTGGATAGGAGCCAGATTTATGAATCGG
AGATGAAGAGGAAAGGCAGCAGTGGGGATGACGTGGAGGATTTGA
GCATCGATGGCTCCAGTTTCAACTATGATTCCGATGATCAATTCTTGG
ACAGCAGTGGCGCTGTGAGCAAGGGGGAGTCCGAGAGTGTTAGGA
ATGGCGACGGCAAGGGGAAGAAGAAGGGGCTGCCGGCGAAGAATT
TGATGGCGGAGCGCCGCCGCAGGAAGAAGCTCAATGACAGGCTCTA
CATGTTGAGGTCAGTGGTGCCAAAGATTAGTAAGATGGACAGGGCT
TCAATACTAGGGGATGCGATAGATTACTTGAAGGAGCTCCTGCAGAA
GATCAACGACCTCCACAACGAACTGGAGGCCATCCCGGGGGCGGGG
GCGTCCACCCCCACCCAAAACACGAGCTTCTACCCGCTGACGCCTA
CAACTCCTGGAATCCCTGCCCGGATCAAGGAAGAGCTCTGCCCGAG
CGCCTTTGCAAGCCCGTTGTCCAGCCCCACCGGACAACCGGCTAGG
GTTGAGGTGAGACTAAGGGAAGGCAGAGCAGTTAACATCCACATGT
TCTGTGGCCGGAAACCTGGGATTCTGCTCTCCACTTTGAGGGCACTC
GACAATCTTGGGCTTGACATACAGCAAGCTGTCATCAGCTGCTTCAA
TGGCTTCGCACTCGATATTTTCCGAGCTGAGCAATGGGGAGAAGGG
CAAGAGCTGAACCCTGACCAAATCAAGGCAGTGCTCCTCGATTCAG
CTGGCTTCCATGGCATGCACTGA

SmbHLH110 ATGAACAGTGGTTTGCCGGAGATGTTGCATTGCCTCAGCTCGACGTC
GGAAAACTGCGAAATGAGCGTGCTCGAGCGGCAGCGCTCGCGCCTC
AAGTGGCAGCAAGAGCAGCTGAGCCAACAACAGCCACCAATCCTA
CCTTACTTCAATGGCAACGACCAGCTCAGCTACTTAGGCTTCCACGG
CGATCAAGACCTCTGCGAGCTGGTGGTGAACGGCGGCGCCATGAAG
CCGGACCCCGGCATCGAAAACGACGACTTCCGGAGGTTTCTGAATT
ATGGCAACGGTGGGGACTTGGAAATGAGCTGCAATCTCCCTAGAAC
TGTCAGCTGCCCGCCCAATGTGGCGGCCAGCGTCGACTCGGCCGCC
GGGAAAGAGAGCTGCAAGAAGAGGAAGGCTGACAAAAGCCACAA
CCTCAAGATAGCTGCAGAGGAGAAAAAGATGAAGGGATGCGCAGA
GGAGGAATCTAAGATGACAGAGCAAAACAGCAACAGCAAAAGCAC
CACCACCACCATTAGCACTTCAGCTAACAAGAACAGCAAAGGAATG

| | |
|---|---|
| | TCATCTGCTAATGCGAAGAAGGTCGCCGACGCGCCCAAGACCGATT<br>ACATCCACGTCCGGGCGCGTCGCGGCCAGGCAACTGACAGCCATAG<br>CTTGGCTGAAAGAGTTCGACGCGAGAAAATAAGTGAGAGGATGAA<br>ATACCTGCAAGATTTGGTCCCCGGGTGCAACAAGGTCACCGGGAAA<br>GCCGGTATGCTCGACGAGATCATCAACTATGTGCAATCCCTGCAAAG<br>ACAAGTAGAGTTTTTGTCAATGAAATTAGCTGCAATCAATCCAAGGC<br>TGGATTTTGACATCGAAAACTATCTACTTAAAGAGATTTTTCCTGCCT<br>GTGCTCCGGCAGTCGAGGCCTCGTCAACCGGCATGATCGATCATTGC<br>CACCTGCAGTTCAATACACCAGTACAAGGAGTTGCGGGTTCGGGTC<br>TAGAAATGGCTGTGGATCCAATGGATGCTACGCTTAGGAGAACCATA<br>AGTGCCCCTGTAACAGTCCCACAAACATTTCTTGATCCATCCAGTTT<br>GAATCAAATTCAGCACGTAACATGGGAGGATGAGTTGCAGAATCTCT<br>ACAGTATGGAGTACCAACAAGGAAGATCTGCATCATTTATTTCTCAG<br>CAAATTACAGGTTACCTCGAGGCAAACCATGTAAAGCTGGAGATGT<br>GA |
| SmbHLH111 | ATGGATTACTCTTCCTTTTCAAACCCTAGTTCGTGCGGCTCCACTTCC<br>TCCGAGAAGAGCAAGAAGAAGAGGGGAGTGGCCGCCGTGAAGCTG<br>TCGACGGACCCTCAGAGCGTGGCGGCGAGGCAGCGGCGCCACCGC<br>ATCAGCGACAGGTTCAAGATTCTGCAGAGCCTGGTGCCCGGCGGCT<br>CGAAGATGGACACGGTTTCGATGCTGGAGCAAGCCATACAGTACGT<br>CAAGTTTCTCAAGACTCAGATTTGGCTGCAGCAGGCCATGATCAACT<br>TAGTTGATGTTGATGATGCTGCTCCCACAATTTCCAGTTTCCAACCCA<br>ATAATAGCGCTAGCGCACTCCCTCCGCTTAATCATCAGGGCTTTGATT<br>GTTACGCGGTGGATCGCTTGCTGCCGGAGTGTGGCCAGTTGCCAGA<br>ATCTTGGTTTTCCGGTCAAGAGAATTGGGCTTCTGATGCGTCCATGC<br>ACAGCTGA |
| SmbHLH112 | ATGGATCATCAAAACCCTAGTTGTTCGAGAGCAGACAGAAAAACCA<br>TAGAGAAAAACCGGAGAAATGAGATGAAGGCCCTCTACACCAACCT<br>CAACTCTCTAATTCCTCCTCAACCCCATTCCCGACCTAGGGAAATGG<br>TTTCGTTGCCAGATCAGCTGGAAGCAGCGACGAACTACATAAAGAT<br>GCAGCAGGCTAAGTTGGAGAAGCTGAAGCAGAAGAAAAACTGCCT<br>TGTTTGGAGCAAAGGAGGGCCTAATAATTTACCAAACATCGATTTTC<br>GTGTATCGGGTTCAGCCCTAGAAGTGGTGCTCATAACTGGACTTAAT<br>TGCCAATTCATGTTCACTCGAATCATACATTTGCTGCATGAAGAAGCC<br>GCTGAAGTTGTCGCCGCTACTTTCTCTGTACTCGACAACACCGTTTT<br>CCACACTATTCATGCCAAGATTGGAGATCAGTCTGCACAGAATCATG<br>GAGCTGCGAGGATTTCCGAGAGATTGAAGAAATTTGCGTACGACTG<br>TATTTAG |
| SmbHLH113 | ATGAAAATGTTGTCTTACCTGGAGCCTTGTGTTTGGCAGGCAAATTT<br>TGCTGGACTTGATTATGAGCTTAACAATCATCAACAACAACAAGATT<br>CAATGAATCAAGAAATGGGAGCAACATCTGCAGATCAGAACAGCAG<br>CCAAATCGTAGAATACATGCTGCATAATCCACCACAACACCACCAAC<br>CATCATCCAACTTTTGCGGGTCGAATTCATTTGACAAGTTGAGTTTTG<br>CAGATGTGATGCAGTTTGCAGACTTGGGCCCCAAACTGGGGTTGAA |

TCAAGGCAAGACATCCGAGGAGGAAGGCGGGATCGACCCCGTCTTC
TTCCTCAAATTCCCGGTCTTGAACGACCATCACAAGGTGCAGCAAG
ATGACGAGAACCAACACCTCTCTTTGAGAGAAGATGAGCAAGGTGG
TGACGATGATGGAGATCGAGTGGAGGAGAATGCGCAGCTGAGATTT
GTTGGTGAGAATCTTGAAAAAGTAGAAGGGAAGAGCAAGAGGAAG
AGGCCTCGAACTTCAAAGACTATTGAAGAAGTTGAGAGCCAAAGAA
TGACTCATATTGCTGTCGAAAGGAATAGAAGGAAGCAAATGAATGA
ACATCTTCGTGTCTTGAGGTCTCTCATGCCTAGCTCCTACGTTCAAAG
GGGTGATCAAGCATCTATAATTGGTGGAGCGATTGAGTTTGTGAGAG
AGCTGGAGCAGCTCCTCCAATGCCTGGAGTCGCAGAAGAGGCGGCG
GCTGTACGGGGATGGGGGGAGGCCGGCCGGAGATCCGTCGTCCATG
GGTGTGCAGCAGCCTCAGATGTTCCCTCCAATGGCTATTCCAAATGA
TCAAGGCTTTGAAACTGCAGGATTGCAAGAAGAAAGCGCAGAGAG
CAAGTCATGTTTAGCAGATGTGGAGGTGAAGCTTTTAGGGTTTGATG
CTTTGGTCAAGATTCTGTCGAGGAGAAGGCATGGACAGCTCATCAA
AACCATTGCTGCCCTTGAAGATTTGCACCTCACTATTCTGCATACTAA
TATTACTACTATTGAACAAACTGTTCTTTATTCTTTCAATATCAAGATT
AATGGAGAGGCAAGGTTCACAGCTGATGATCTGGCAAACTCAGTTC
AACAAATTTTCAGTTTTATCCATGCAAACAATAGCATATAA

SmbHLH114 ATGGACATTGAGTTCATCAAGAATGGCGGCGAAGATCAGATGGAAAT
GATGCTGATGCAGATGGAGAAGCTCCCCGATTTATCGGGGGCCTACG
CCGACGTCAACGACCTTCCCATAATCGACTTCGCCCCTCACAATCAA
CTTACCGACTTCCAAAACCTACATGCTTCCTCGCCGCCGCCGCCGCT
CCACCCGGCCTCCACGATATCCTTCAGCGGGCAGTTCCAGCCGGGG
GAGGCGGCGCAGAAGCGGAGCTCCATGGCGGCGATGCGGGAGATG
ATATTCCGGATAGCGGCAATGCAGCCGATCCACATCGACCCGGAGTC
GGTGCGGCCGCCGAAGCGGCGGAACGTGAGGATATCGACGGACCC
GCAGAGCGTGGCGGCGCGCCACCGCCGGGAGAGGATAAGCGAGCG
GATCAGGATCCTGCAGAGACTGGTCCCCGGCGGGACCAAGATGGAC
ACGGCGTCCATGCTCGATGAGGCCATCCATTACGTCAAGTTCTTGAA
GAATCAGGTGCAGTCGCTCGAGCGGGTCGCCGCCAACCGCCCGCCG
CCGCCGGGGATGGGCTTCCCGGTGCCCATGTCGAGTGGGAGCTATTT
CCCGGTCGCCGGGAAGGGTTATCATCAGGCGGCCAACGTGCAGCAT
CTGGCGGAATGA

SmbHLH115 ATGGCTCTGGAAGGTGTGATTTACCAGCAAGATCCACTCATCTATGG
CTGCATAGATTACAACTACTGTACGGAATTGGGCGTTGAAGAGAAGT
TTAATGTGGAACAGAGTAGTGCAATTGGATATTGGGAGTCATCTCCG
CCTCTCAACGCCACTTCTTCAGCGGAGGCCCGCCGCAAGAGGCGCC
GGAGCAAGACCTTGAAGAACAAGGAGGAGCTGGAGACCCAGAGGA
TGACTCATATCGCCGTCGAGAGGAATCGCCGCCGCCAGATGAACGA
CTACCTCGCCGTCCTCCGCTCCTTGATGCCTCCTTCCTACGCTCAAAG
GGGAGATCAAGCATCGATTGTTGGTGGTGCGATCAATTTTGTGAAGG
AACTGGAACAACTACTTCAATTTCTGGAAGCTTACAAGTTGATGGAT
AGACAAGACATGGGCAGCAGCAGTGACAAAGTATTTCGTAATTTGTT

GAGCTTTCCTCAATACTCTGGGAAGTCGGCGGACATTGAGGTGACG
ATGGTGGAGACTCATGCAAGCATCAAGATCTTAGCAAAGAAAATGC
CTAAACAGCTCCTCAAAATGGTGGCTGGATTTCAATCATTTTGCCTC
AACATTCTCCATCTTAACATCACCACCCTTGATCAATCTGTTCTATACT
CTTTCAGTGTTAAGGTATGTTTTTATTTCACCCACTCATTAAAGACAA
AATTGAAAAATCTCACTCTACATGATATGTAG

SmbHLH116 ATGGGCACTCAAAATTTGTCCGACTTTTTCAAAGAATCCGAGATGAC
CGATGATATTTTCAGCATTCTCGAAGCTTTCGAAAATGCTTCCGATTG
CAATACTTTGAGTTCCAAAAAATCACCTTTTCTAGAATCTGAGATTG
ACGCCGCCGCCGAAGCTGCTTCGCCCAAATGCAAGAGGCAGAAGCT
GAGCGCCGCCGCCGCCGTGGAAGATGGGCAGCCACGAATGTTGTCT
CACATTTCAGTAGAGAGGAATCGAAGAAAGCAAATGAATGAGCATA
TCTCCGTGCTTCGTTCTTTGATGCCTTGCTTTTATGTTAAAAGAGGTG
ACCAAGCATCCATAATAGGAGGAGTTGTGAACTACATCAAAGAGCT
GCAACAAATTCTGCAGTCAATGGAAGCCAAGAAGCAAAGGAAAGC
CTACGCCGATGTTCTGAGCCCTCGGCCCGGACCTCTCAGCCCGATGA
GGCCCGCATTGAGCCCTATCCTAAGCCCGAGGACCCCCCAACCGAG
CAGCCCGTACAAGCCTAGGCTGGGCTTCCTCAATTCGCCTTCCCTCC
CGAGCCCGGCCGAGCTCTCCCCGTGCAATTCTTCGACCAACGACAG
TGTGAATGAGCTCGTGGCCACTTCAAGATCGGCTGTGGCTGAAGTG
GAGGTCAAATTCTGTGGCCCGAATCTACTGCTGAAGACGGTGTCGC
ACTCAATCCCGGGCCAGGTTGTGAAGATTGTCTCGGCCCTCGAAGA
CCTTGCACTCCAGATCCTCCAAGTCAATGTCAACAAGCTTGATGAGA
AGATGCTCCATTGTTTCACCATTAAGATCGGAATCGAGTGTCAACTTA
GTGCACAAGAATTGGCTGAGCATATTCAACAGACATTCTGCTAA

SmbHLH117 ATGGCCATAATCCAAGCCAGCGCCTTCTACGATCAAGATTTCGGTTT
GCTCCACACTTCATCAAACTCCTCCCCCGAGGACCATATTTACGGGT
CCAGGCCCGTTTATCGGGAGCCCGAGAGCCCGGCAAAGAATTTCAG
CATCAGCAATTCCTCTACGGTTTCCAGCCCTAGCAGCACCAATTCCA
ACGCCGGCCATTCTGTCATCAGCTTCAAGCCCGAATTCGGCAATTTC
ATCCATGTCAACGCCGGATCGTTGCTCAGCTTCGATCAGAGTAACAC
CAATTTATTGATCGCTGAAGAAGACGACTATCCCATGTGGGAAGGTG
ATCGTGATTTGAATTACCAGAGCCACCTCAGCGCCGCCGCCAATAAT
CATGGCCCGCCTGAAACTTCCGAGTACGAGGGCGGACCGCACTTGA
ATGATCAGGATCACGAGACTAGTGGAGTTACTAATGGTGGTCGGGAA
ATAAATAAACGCCCATCTACGGGAGAGAATCTGCAAGCGGTGAAGA
AGCAATGCGTCGTTGCTGCCAAGAAGGCGAAACCCAAGGCTGCAGC
AGAATCCAAAGATCCACAAAGTGTTGCAGCTAAGAATCGAAGGGAA
CGGATCAGTGAACGCCTAAAGATACTGCAAGAACTCGTTCCTAATGG
TTCCAAGGTTGATCTTGTTACCATGCTAGAAAAGGCAATCAGCTATG
TCAAGTTTCTTCAACTGCAAGTGAAGGTATTGGCTACTGATGAATTT
TGGCCGGCGCAAGGTGGAAAAGCTCCGGACCTCTCTCAAGTTAGAG
AAGCCATTGATGCGATTCTTGCATCACAAAGAGACCGGAAATCTACC
TCCAAATGA

SmbHLH118 ATGGAGTTGTACAGAAATGATGAGCATGGTTTCTTGGAAGAGTTACT
AGAAATGGAGTCCTTCACAAATCAAGAAAGCTTGCCTAGTCTTGCTT
GTATCCCTTCCTACCAACTATCACCGCCCGACTTCAGCTTCATCCAAA
CGCTCCCTTATGATTTTCAAAATCCCGAACCCCACAGTTTGAACACG
AGCCCTCAGAAGCCGGCGTCGAAGAACCTCATGGCGGAGAGACGG
CGGCGGAAGCGCCTCAACGACCGCCTCTCCATGCTCAGATCAGTTG
TTCCTAGGATTAGCAAGATGGACAGAGCATCTATTTTGGGAGACACC
ATAGACTACATGAGAGAACTGCTGGGAAGAATCAACAACTTGCAGG
AAGAAACGCATTTATTCAAGGATGTCAAACCCAACGAAATTTTACTC
AGAAATTCGCCCAAGTTTCAAGTGGAAAGAGGAAATCCGGATACAA
GAATTGAGATTTGCTGCGGCGGGAAGCCGGGATTGTTGCTGTCAAC
GGTGACTACATTAGAAGCATTAGGCGTCGACATTCAGCAGTGTGTTA
TTAGCTGTTTTAATGACTTTGCACTGCAAGCCTCTTGCTCAGAGGTC
TCTCGTTTCTTCGTCTTTTTTGTGTAA

SmbHLH119 ATGTTCAGGAGGAGGCCATCAGCGGGATACCAGTCAGAACAACAGC
TACGGCTGCTCTCGCAGTCACTAGGGCACTACGGCGCCTCCAGCGTC
CCCTTGGGCCTCCACGCGGAGCTGCAGAAGCTCTCCGCCCAAGAAA
TCATGGACTCCAAGGCATTGGCCGCGCCCAAGAGCCACAGCGAAGC
CGAGAGGTGGCTGAGAGAGAGAATCATCAACAATCATCTTGCTAAG
CTGAGAAGCTTGCTCCCCAACACAACCAAAACGGACAAAGCTTCAT
TGCTAGCCGAGGTAATTCAGCACGTGAAGGAGCTGAAGCGGAAGAC
ATGCGTGCTAGTGAAGACGAATTTGGTTCCGACAGATATAGACAAAT
TAAAGGTGGATAACGCGAGCGACAAGGAGGGGAAATCGGTGATCAA
AGCCTTGATTTGCTGCGAGGATTTTAGCATAAAAAAATTTGGTTGAAA
TCTGCTGA

SmbHLH120 ATGGCAGCCTTTTCATCAACATCATTTCACCACCACCATCCTTTTCTC
CTCGACTCTCTTTTCCACCCAAACGCAACCACCATTGATGCTATTGC
CAACAACAATCATCTCTCCTCTCACAACTTCAATCCTTCTTTTCTCCC
AAACATGGATAACAATTCCTCTTCCATTGTGACTGATCATAAGCATGA
AAGCAATGACCAAATCACTCAAAACTTCATCACTCCCATGGACAATA
AGAAGAGGAAATGCAAATCCCGCTCTTCTGCAAATTCTGCTCAATCC
AAGGATAAGAGAGAAGTGAAGGGAAAGAAGCAGAAGAAAATAAAA
GATGATGAAGAAAAGAAGAATAACTCCAAAGAAATTAAAGCTGCAG
AAGCAGCAGGCTACATACATGTAAGGGCTAGGAGGGGCCAAGCTAC
TGATAGTCACAGTCTTGCTGAGAGGGTAAGGAGAGAGAGAATAAGT
GAAAGAATGAAGCTGCTACAGACTCTTGTTCCTGGTTGTGACAAGG
TAACTGGGAAGGCCCTCATGTTGGACGAAATTATTAATTATGTTCAAT
CCCTCCAAAATCAAGTTGAGTTTCTCTCAATGAAGCTTGCTTCTGTG
AATCCCATGTTCTATGACTTTGGGGTGGACTTGGAATCATTCATGGTT
AGGCCTGATCAGGATCTTAGTGACTTGCCATCCCCAACAAGCCCACA
TGCAGAATCATTAATGGTTGCCAACAACTATAATTTTCTTGAAAATCC
TCTTCTGTTTCAACAAGCTCAACTTCCATGTGTCCTCCCATACCCACA
GACCTTGTGGGAAGTGGATGAGCATAGACAAAAAAATTTCAATCAG
TCAGGATTCAACAACAGCCTGTTTTCATTCCATTAA

SmbHLH121 ATGGAATTGCCTGTTGGAACTGAAGGAAGAAAGGCGACTCATGATT
TTCTCTCACTATATTCGTCATCAGCTGCACATAACCAACAAGATCCGT
CTCCCTCGCAAGGTGGCTACCTTAAAACGCATGACTTCTTGCAACCA
TTGGAACGGGTGGGGAAGCATGTAACAACGACGAAAGAAGATATTA
AAGCTGGGGTGGCTGCTGTTGATAGGCCTCCAGCGCCAGCTCCTCC
AGCCTCAGTGGAGCATCTTCTTCCTGGTGGCATTGGGACGTACAGCA
TCTCTTATTTCAATCAACTGGTGTTAAAGCCTGAGGGGAGCTTGTTT
ACTGCTCAGCCAACCAGGAATGATGAAAACTCAAACTGCAGTTCTT
ACTCGGGAGGTAGTTTCACTCTGTGGGATGAATCTGCGGTTAAAAAG
GGAAAGACAGGGAAGGAGAATATTGCCACAGAAAGACATATTCGCG
AGGTATCGGGTGTTAATGTGGTTGGAGGGCAATGGGCGCCATTCCTG
GAAAAGCCATCACAATCCTCTTCTAACCATAAGCACAACACCACAA
ATTTCAGCACTTTCTCATCTTCTCAGCCATCATCATCTCAGAAGAACC
AAAGTTTCATGGATATGATGACATCAGCTAAGAATGACCAAGAAGAC
GATGATGATGATGAAGAAGAAGAGTTCACCATCAAGAAAGAGCCAT
CACCCCACTCAAAAGGTAATTTGTCTGTGAAAGTTGAAGCAAAGAC
TATAGATCAGAAGCCTAATACACCACGCACTCAGCTACAGAGCAACG
AAGGAGGAGCAAGATTAATGACAGGCATATTTCAGAGGCTGAGAGA
GATCATTCCTAATAGTGAGCAGAAGAGAGATAAGGCATCGTTCCTCT
TAGAGGTTATCGAGTACATTCAGTTTTTACAAGAGAAAGTAAACAGA
TATGACAGTTCCTATAACATCTGGAATCATGAGCCAGCCAAAATGAT
GCAATGGAGGAACTGTCACGTGAGTGAAGGTTTTGTAGAACGTGCA
CGAGGAACAAGCAGCGATTCTGCTCCAGCGTCAGTATTTGGCAAGA
AGTTCGATGAGAGCAAAGCTGCTGTCTCCCCCAGCCTACCAATCAG
CGGGCAAAACCTAGTAGACTCAGATTTGAGCACTGCTACTACTTTGA
GAGAAAGAGTGGTTCAACTGCCCGAATCAACCTCTAAGGCAGCGAC
AATTCATTCGCCAATGCAGCCAAATATCTTCACTCTTGGTGGTAGGAC
AACAAGCATTCCAGCTTCCCCGATTTCACCAAAACAGGCATCCGATC
TGGAGAAAGCTATGACATGGCCTCCATCTCAGCTTCGATCATGTACC
ACTGACACTAAAATGACAGAACATGAACTGACCATTGAAAGTGGCA
CCATCAACATCTCTAGTGTCTATTCCCAAGGGCTGTTGAATACTCTTA
CACAAGCGCTACAGAGCTCTGGAGTGGATCTATCCCAGGCCAGCATC
TCAGTACAAATCGATCTGGGGAAGAAAGCAAACGGCGTCACACATT
CTTCTACCTTTATCTCCAAGGAGGATGAAGTTGACACCACCTTACCG
CGTTCTGCAACGGCTAGCACGGGGAAGGAATTCGGGCGTGCTGCGA
AGAAACTAAGGACTAGCTGA

SmbHLH122 ATGAATCACTGCGTTCCCGAATTTCATGAAATGGAGGACGACGATTC
CATTCCCACGCCTTCCACTTTCTCTAGACCCAAAAGGACTGCGACAG
GTGAGGAGGAGATCATGGAGCTGCTATGGCAGAACGGTCAAGTAGT
GGTGCAGAGCCAGAACCAGAAACCGCCCAAGAGGACCGACTCCTG
CGGCGGCAGCGGAGAGGTCGTGATTCCGCCGGAGAGGGAGATCCG
ATCCACCGCCGAGGAGCACCAGCATTTGTTTATGCAGGAGGATGAG
ATGGCGTCTTGGCTTCAGTATCCACTCGATGATTCCTTCGATCGCGAT
TTCTACGCCGATCTCCTCTACTCCGCTCCGCCGCCGCCGCCACTCATC

ACCGCCACCGCCAACGCGCAGCCTAGGGCGGCAGCGGAGATCCGCC
CCCCGCCTATCCAGACGCCGGTTGCCAGACCTGACAATCCGCCGCG
GGTGCACAATTTCGTGCATTTCTCGAGGCTCCCGATCAGGCCGAGGA
TGGCGCCGCAAGAATCGTCGACGGTGGTGGAGTCAAACGAGACTCC
GACGGGCGCACGGGAGTCTAGGGTTTCTCACAAGGTGACCGACAGC
AGGCCGCCGGTGCACGCGGATACCTCCACGGTGAGAACTAGAGACA
CGTCGGGTGCCGCGGGTACTTGTGACCTCACCGTCACCTCGTCGCCC
GACGGCTCCCCCGCCAGCTTCAGCGGAGAGACGCATCAGCCGCAGC
AGAAGCCGGCGGCTGATCGGAAGCGGAAAGTAAGAGAAGCCGACG
ATAATGAATGTCAGAGTGAGGATATCGAGTTTGAAGCTTCCGAAGGT
AAAAAACAAGGCCGTGGTTCATCATCGACAAAGAGATCACGTGCTG
CAGAAGTTCACAACCTGTCGGAGAGGAGGCGACGGGACAGGATAA
ATGAGAAAATGAGGGCACTACAAGAGCTCATCCCACGTTGCAATAA
GTCAGACAAAGCTTCAATGCTGGATGAGGCTATCGAATACTTGAAAT
CACTTCAATTACAAGTGCAGATGATGTCGATGGGTTGTGGGATGGTT
CCCATGATGTACCCGGCGGGCATGCAGCAGTACATGCCTGCAATGGG
GTTGGGCATGGGGATGGGGATGGGGATGGACATGGGAATGAGTCGT
CCGGTGGTCCATTATCCACCTATGATGGCCGGCTCAGCCATGCCGAAT
CCTGCAGCTGCGGCAGCTCAAATGGGTCCTAGATTTGCCCTGCCACC
ATTTCATATGCAACCAGTTCCTGTACCTGATCCTTCAAGATCACAACC
TACTAACCACACAGATCCCGTGCCAAACTCCACTACTCCTCAGAATC
ACAACCACCCAAGGTTTCCAAGCTTTTCGGATTCGTATCAACAGTAT
CTTGGTTTCCATCCCACACAACTACCAATACCACAGGCAAGATTGAT
CTCTATAAATCAAGGTGTGGTGCAGCATGACGACAACAAACCAAGC
ACCAGCAAAGACATCGGCAACCCTCATAACCAGCAAACTGGATGA
SmbHLH123 ATGCATTGTGAGAATCAGTATGGTTTCAACATTGAGTCTGATGGTGG
AGAGTTTGTGAATCAATTGTTGCATAACTCAAGTAAGAATGTTGAAA
ATAGAAGCTGTTTGTTGATGCCTGTTAAGCAGTCTTTGGTGTTGGAT
GGTGAGAAGGGGGAGTTAGTGAAGGCTTCGGGGAGAATGGGGAAG
AGAATTGGGGTTTCGGAGACGAAGACAGTAGAGGCTTTGAGGAGCC
ATAGTGAGGCAGAGAGGAGGAGGAGGGAAAGAATCAATGCACACT
TGGAGAGCCTTCGGGGGCTGGTACCGAACAATGAAAAGATGGACAA
AGCGACCCTACTAGCTGAAGTCATCAGCCAAATAAAACAGCTGAGG
ACAACCGCGTCACAAGCCAGCGAAGGCCTGCACATCCCGATAGACA
CGGATGAATTAAAAGTCGAAACACTTGAGAATCATACTGGTGATGGC
ATGTTCTTGCTTAGGGCTTCGCTCTGCTGCGAACACTTGCCTGATCT
GCTGTCTGATGTGAGGCAGGTGATCAACAGCCTTCCTATTCGAGTGC
TGAAGTCCGAGATATCTACGTTGGGAAGCAGGGTGAAGATTGCGTTC
TTGATCACGACAGTTGAAGGGAACGATTCTGTTAGGGAATTTGTGGT
CGGTTCAGTTCGTGCAGCTTTGAGCAATGTTCTTGAGAAGGTGTCTG
CATTGGCGGAGTCTGCTGAGCAACTGTTTATCCCTCGTAAGAGGCAG
CGGGTTTCGTGTCTCGATTCTTCGTCGTACTTGTTCGAGTGA
SmbHLH124 ATGTCGAGCCGAAGATCAAGATCAAGGCAATCTGGATCTTCAAGAAT
CACAGATGATCAAATCAACGAACTCGTTTCCAAGCTGCAACAGCTTC

| | |
|---|---|
| | TTCCCGAGATGCATAATAGGCGCTCAGACAAGAAGTCAGCAACCAA AGTGTTGCAGGAGACATGCAACTACATTAGAAGCTTGCACAGAGAA GTTGATGACTTGAGTGAGAGGCTGTCTGAATTGCTTGAAAACGCCG ACACTACTCAAGCTGCTCTTATTAGAAGCTTACTTATGCAGTAG |
| SmbHLH125 | ATGTCTAGCAGAAGATCGCGTTCGAGGGCGGCGTCGGGATCCTCGA GGATAACCGACGATCAGATCGCCGACCTCGTCTCAAAATTGCAGCA ACTCATCCCCGAGATCCGCAGCCGCCGTTCCGACAAGGCTTCGGCTT CGAAGGTGTTGCAGGAGACGTGCAACTACATAAGGAACTTGCACAG AGAGGTGGATGATCTGAGCCATCGATTGTCGGGGCTGCTGGAATCGA CGGACGGCGACAGCGCTCAAGCCGCCATTATTAGGAGCTTGCTATTG TAA |
| SmbHLH126 | ATGGACAACCACCAAAAGAAGTCTTATGCCACCACTAGCGGTGGCG GCGCTTTCGGAATGTGGGAACCCAGCAGTAATCCTATAATGTTTTCA GTCGCGCAGTCGAATCGGGGCAAGAACGTTGGCGACGGTCTTGACG CCATCGAATCTCAGTTTCAGTTTAGCATATCGCAAACAGCGCAAGAC ATGGCATCAATGGAGAAGTTGCTCAACATCCCACAGGATTCTGTAAC ATGCAAGATTCGAGCAAAGCGCGGCTTTGCTACGCATCCTCGTAGCA TTGCGGAACGGGAGAGAAGGACACGAATCAGCGGCAAGTTGAGGA AATTGCAAGATCTTGTTCCAAACATGGATAAGCAAACTAGCTACGCC GACATGCTGGATCTAGCAGTGCAACACATAAAAACCCTTCAAGATCA GGTTGAGGCATCCGAGATCTGCAGCAACCTCAGCGAAACCACAACG ACGACAGAAGGTCCGAGCCCTCTCCGGGCAGGCACGACCCGTGGA GGAGAGGGGGAGGCGCCGGGAATAGAAATAGTGAAGGAGAGGGGA AGGGGCCGAAGAGAGAGGGAGGTCGGCGTCGCGGTGGAGGGAGGT CGGCGTCACGGTTCACCTCTGCATCGCCGCCAAAGCTCCTCGTCAG AGGCGCCACGAGTGAAGAGATCTGCGGCTGTAAGGCGCGAAGCTC GCGAACTGTCTCGCCGTTGCTAG |
| SmbHLH127 | ATGGAGAAAACTTGTGGTTATTCAAAAGCAAGAGCAAACTGTCAAG ATTCATCATCATCATCTCATATATCAGCCATGCAAGCAATGAAGAAGA GGAAACGAGCAGCACCTCATTCACATTACAAGAGGCGTAGAGATAA TAAAATCAACAAGAGATTGCGGGTACTACGAAACCTCATACCTAATT GCACCCAGATGGATAAAGAATCAGTCCTTGATGAAGCTATTGTGTAT CTAAAATGTCTTCAGCTTCAATTACAGATAATGCCAAGTTTTGGTGG ATTATGTGTCCCAGCAACACCCATACAGTTGTTTGGTTTACCTTTGGG TGGATATGGAACGATGGCACAACAACCATGA |

**Table S2.** Gene features and structure of *SmbHLHs*

| Gene name | Accession number | Gene length (bp) | cDNA length (bp) | Protein (aa) | Mw (Da) | p*I* | Exon no. |
|---|---|---|---|---|---|---|---|
| *SmbHLH1* | KP257434 | 1985 | 930 | 309 | 34239.7 | 6.0 | 10 |
| *SmbHLH2* | KP257435 | 2071 | 813 | 270 | 28311.8 | 6.0 | 6 |
| *SmbHLH3* | KP257436 | 1261 | 843 | 280 | 31109.1 | 5.8 | 2 |
| *SmbHLH4* | KP257437 | 3079 | 1032 | 243 | 37450.3 | 5.7 | 7 |

| | | | | | | | |
|---|---|---|---|---|---|---|---|
| *SmbHLH5* | KP257438 | 685 | 618 | 205 | 23366.6 | 5.3 | 2 |
| *SmbHLH6* | KP257439 | 1197 | 930 | 309 | 34841.1 | 8.4 | 4 |
| *SmbHLH7* | KP257440 | 787 | 717 | 238 | 27152.7 | 5.3 | 2 |
| *SmbHLH8* | KP257441 | 1864 | 1251 | 416 | 46178.3 | 7.0 | 9 |
| *SmbHLH9* | KP257442 | 1531 | 960 | 319 | 34832.7 | 7.0 | 3 |
| *SmbHLH10* | KP257443 | 4778 | 714 | 237 | 26728.7 | 9.2 | 9 |
| *SmbHLH11* | KP257444 | 1319 | 462 | 153 | 16679.6 | 9.6 | 4 |
| *SmbHLH12* | KP257445 | 785 | 714 | 237 | 27436.4 | 8.1 | 2 |
| *SmbHLH13* | KP257446 | 1823 | 1710 | 569 | 62947.9 | 7.2 | 2 |
| *SmbHLH14* | KP257447 | 1605 | 927 | 308 | 34233.5 | 8.6 | 7 |
| *SmbHLH15* | KP257448 | 1214 | 828 | 275 | 30654.8 | 5.5 | 3 |
| *SmbHLH16* | KP257449 | 3647 | 1401 | 466 | 52210.1 | 6.2 | 7 |
| *SmbHLH17* | KP257450 | 1311 | 1125 | 374 | 42207.3 | 5.3 | 4 |
| *SmbHLH18* | KP257451 | 1331 | 1161 | 386 | 43365.4 | 5.3 | 3 |
| *SmbHLH19* | KP257452 | 3010 | 594 | 197 | 21093.8 | 9.3 | 6 |
| *SmbHLH20* | KP257453 | 2701 | 1479 | 492 | 54199.1 | 5.5 | 4 |
| *SmbHLH21* | KP257454 | 618 | 618 | 205 | 22903.9 | 6.0 | 1 |
| *SmbHLH22* | KP257455 | 2474 | 681 | 226 | 25898.3 | 4.9 | 5 |
| *SmbHLH23* | KP257456 | 1288 | 636 | 211 | 24105.8 | 8.2 | 5 |
| *SmbHLH24* | KP257457 | 1313 | 714 | 237 | 25929.4 | 9.2 | 3 |
| *SmbHLH25* | KP257458 | 1802 | 984 | 327 | 36862.8 | 5.2 | 4 |
| *SmbHLH26* | KP257459 | 2091 | 1422 | 473 | 52169.1 | 6.0 | 8 |
| *SmbHLH27* | KP257460 | 3659 | 1011 | 336 | 36568.0 | 5.3 | 7 |
| *SmbHLH28* | KP257461 | 2928 | 672 | 223 | 24401.2 | 6.1 | 6 |
| *SmbHLH29* | KP257462 | 1347 | 1347 | 448 | 49069.2 | 6.8 | 1 |
| *SmbHLH30* | KP257463 | 856 | 687 | 228 | 25858.5 | 7.0 | 3 |
| *SmbHLH31* | KP257464 | 3278 | 1812 | 603 | 67854.3 | 6.0 | 8 |
| *SmbHLH32* | KP257465 | 858 | 696 | 231 | 26201.3 | 9.1 | 3 |
| *SmbHLH33* | KP257466 | 2481 | 1167 | 388 | 41656.4 | 6.2 | 8 |
| *SmbHLH34* | KP257467 | 879 | 717 | 238 | 27196.2 | 9.2 | 3 |
| *SmbHLH35* | KP257468 | 580 | 423 | 140 | 15415.3 | 4.9 | 3 |
| *SmbHLH36* | KP257469 | 1403 | 438 | 145 | 16333.8 | 9.1 | 4 |
| *SmbHLH37* | KP257470 | 1506 | 1479 | 492 | 54549.1 | 5.6 | 2 |
| *SmbHLH38* | KP257471 | 1230 | 1230 | 409 | 44907.9 | 6.0 | 1 |
| *SmbHLH39* | KP257472 | 4407 | 1236 | 411 | 43932.5 | 8.9 | 6 |
| *SmbHLH40* | KP257473 | 1844 | 822 | 273 | 30907.1 | 7.6 | 3 |
| *SmbHLH41* | KP257474 | 2201 | 807 | 268 | 28592.7 | 6.1 | 6 |
| *SmbHLH42* | KP257475 | 2672 | 1473 | 490 | 54450.3 | 7.0 | 7 |
| *SmbHLH43* | KP257476 | 1914 | 1809 | 602 | 65708.4 | 5.8 | 3 |
| *SmbHLH44* | KP257477 | 1236 | 897 | 298 | 34022.7 | 5.0 | 4 |
| *SmbHLH45* | KP257478 | 1889 | 1149 | 382 | 42304.1 | 8.7 | 6 |
| *SmbHLH46* | KP257479 | 1753 | 957 | 318 | 34898.6 | 9.1 | 3 |
| *SmbHLH47* | KP257480 | 2054 | 1110 | 369 | 40519.1 | 6.4 | 8 |
| *SmbHLH48* | KP257481 | 3809 | 723 | 240 | 26367.8 | 5.9 | 6 |

| | | | | | | | |
|---|---|---|---|---|---|---|---|
| *SmbHLH49* | KP257482 | 1310 | 450 | 149 | 16443.4 | 9.8 | 4 |
| *SmbHLH50* | KP257483 | 1147 | 585 | 194 | 21745.2 | 8.6 | 3 |
| *SmbHLH51* | KP257484 | 3403 | 1866 | 621 | 68167.0 | 5.9 | 8 |
| *SmbHLH52* | KP257485 | 1668 | 1071 | 356 | 39170.7 | 6.7 | 6 |
| *SmbHLH53* | KP257486 | 1799 | 1788 | 595 | 65703.0 | 7.5 | 2 |
| *SmbHLH54* | KP257487 | 3784 | 1716 | 571 | 64337.5 | 5.3 | 6 |
| *SmbHLH55* | KP257488 | 1611 | 909 | 302 | 34610.9 | 4.9 | 4 |
| *SmbHLH56* | KP257489 | 714 | 633 | 210 | 22976.5 | 9.2 | 2 |
| *SmbHLH57* | KP257490 | 804 | 621 | 206 | 23026.3 | 8.6 | 3 |
| *SmbHLH58* | KP257491 | 459 | 459 | 152 | 17332.8 | 5.4 | 1 |
| *SmbHLH59* | KP257492 | 1160 | 1083 | 360 | 39840.4 | 6.3 | 2 |
| *SmbHLH60* | KP257493 | 2149 | 1185 | 394 | 43614.7 | 6.2 | 6 |
| *SmbHLH61* | KP257494 | 8663 | 1008 | 335 | 35034.8 | 9.0 | 6 |
| *SmbHLH62* | KP257495 | 1257 | 909 | 302 | 33186.9 | 5.2 | 5 |
| *SmbHLH63* | KP257496 | 1711 | 1248 | 415 | 45886.8 | 5.5 | 6 |
| *SmbHLH64* | KP257497 | 3662 | 1002 | 333 | 37000.0 | 5.1 | 7 |
| *SmbHLH65* | KP257498 | 1609 | 933 | 310 | 34326.6 | 5.6 | 2 |
| *SmbHLH66* | KP257499 | 1980 | 741 | 246 | 27661.7 | 8.3 | 3 |
| *SmbHLH67* | KP257500 | 3403 | 1011 | 336 | 36747.4 | 6.1 | 8 |
| *SmbHLH68* | KP257501 | 741 | 591 | 196 | 21762.7 | 9.2 | 2 |
| *SmbHLH69* | KP257502 | 1568 | 975 | 324 | 36223.3 | 4.8 | 4 |
| *SmbHLH70* | KP257503 | 1989 | 837 | 278 | 29371.7 | 5.7 | 6 |
| *SmbHLH71* | KP257504 | 1322 | 255 | 84 | 9533.9 | 7.9 | 2 |
| *SmbHLH72* | KP257505 | 4751 | 2148 | 715 | 76725.0 | 6.1 | 8 |
| *SmbHLH73* | KP257506 | 1005 | 840 | 279 | 30983.2 | 5.1 | 3 |
| *SmbHLH74* | KP257507 | 1924 | 696 | 231 | 26283.0 | 5.7 | 5 |
| *SmbHLH75* | KP257508 | 1065 | 807 | 268 | 30684.1 | 5.9 | 4 |
| *SmbHLH76* | KP257509 | 1303 | 681 | 226 | 25145.5 | 5.5 | 3 |
| *SmbHLH77* | KP257510 | 1268 | 411 | 136 | 15326.4 | 6.3 | 2 |
| *SmbHLH78* | KP257511 | 3636 | 1275 | 424 | 44699.9 | 6.5 | 7 |
| *SmbHLH79* | KP257512 | 1129 | 786 | 261 | 30127.5 | 5.2 | 5 |
| *SmbHLH80* | KP257513 | 2539 | 831 | 276 | 31265.6 | 6.1 | 6 |
| *SmbHLH81* | KP257514 | 2954 | 2154 | 717 | 79156.6 | 5.4 | 3 |
| *SmbHLH82* | KP257515 | 2658 | 1038 | 345 | 38276.9 | 5.4 | 7 |
| *SmbHLH83* | KP257516 | 2333 | 972 | 323 | 36008.1 | 6.0 | 5 |
| *SmbHLH84* | KP257517 | 2204 | 795 | 264 | 28254.4 | 5.9 | 6 |
| *SmbHLH85* | KP257518 | 1231 | 735 | 244 | 27866.6 | 6.3 | 3 |
| *SmbHLH86* | KP257519 | 2936 | 792 | 263 | 29007.0 | 7.0 | 6 |
| *SmbHLH87* | KP257520 | 1746 | 816 | 271 | 30401.1 | 5.7 | 3 |
| *SmbHLH88* | KP257521 | 1217 | 855 | 284 | 31273.8 | 5.2 | 5 |
| *SmbHLH89* | KP257522 | 3832 | 1368 | 455 | 50375.2 | 7.7 | 11 |
| *SmbHLH90* | KP257523 | 2289 | 1458 | 485 | 52610.0 | 5.3 | 4 |
| *SmbHLH91* | KP257524 | 3107 | 1488 | 495 | 55826.1 | 5.0 | 8 |
| *SmbHLH92* | KP257525 | 1290 | 666 | 221 | 24960.3 | 6.6 | 3 |

| | | | | | | | |
|---|---|---|---|---|---|---|---|
| *SmbHLH93* | KP257526 | 1200 | 699 | 232 | 26488.0 | 9.2 | 3 |
| *SmbHLH94* | KP257527 | 2452 | 894 | 297 | 33315.8 | 6.9 | 3 |
| *SmbHLH95* | KP257528 | 1644 | 495 | 264 | 17995.4 | 9.6 | 4 |
| *SmbHLH96* | KP257529 | 1243 | 645 | 214 | 24274.1 | 6.9 | 6 |
| *SmbHLH97* | KP257530 | 662 | 270 | 89 | 9800.1 | 9.3 | 2 |
| *SmbHLH98* | KP257531 | 751 | 648 | 215 | 24154.4 | 6.6 | 2 |
| *SmbHLH99* | KP257532 | 4838 | 825 | 274 | 30443.5 | 7.1 | 3 |
| *SmbHLH100* | KP257533 | 2908 | 984 | 327 | 35889.7 | 5.7 | 7 |
| *SmbHLH101* | KP257534 | 2174 | 750 | 249 | 27200.1 | 5.9 | 6 |
| *SmbHLH102* | KP257535 | 877 | 660 | 219 | 24802.1 | 8.3 | 2 |
| *SmbHLH103* | KP257536 | 891 | 672 | 223 | 25636.1 | 8.6 | 3 |
| *SmbHLH104* | KP257537 | 778 | 708 | 235 | 26797.3 | 5.1 | 2 |
| *SmbHLH105* | KP257538 | 2932 | 699 | 232 | 25850.6 | 7.0 | 6 |
| *SmbHLH106* | KP257539 | 1729 | 696 | 231 | 25333.9 | 9.1 | 6 |
| *SmbHLH107* | KP257540 | 1935 | 1383 | 460 | 51183.3 | 6.4 | 8 |
| *SmbHLH108* | KP257541 | 2415 | 864 | 287 | 32460.4 | 4.9 | 7 |
| *SmbHLH109* | KP257523 | 2289 | 1458 | 485 | 52610.0 | 5.3 | 4 |
| *SmbHLH110* | KP257542 | 2075 | 1203 | 400 | 44393.1 | 5.9 | 7 |
| *SmbHLH111* | KP257543 | 474 | 474 | 157 | 17313.5 | 7.7 | 1 |
| *SmbHLH112* | KP257544 | 706 | 519 | 172 | 19466.4 | 9.3 | 3 |
| *SmbHLH113* | KP257545 | 1505 | 1158 | 385 | 43467.6 | 5.3 | 3 |
| *SmbHLH114* | KP257546 | 660 | 660 | 219 | 24421.0 | 8.1 | 1 |
| *SmbHLH115* | KP257547 | 1085 | 735 | 244 | 28000.3 | 9.0 | 2 |
| *SmbHLH116* | KP257548 | 1654 | 882 | 293 | 32184.0 | 7.0 | 4 |
| *SmbHLH117* | KP257549 | 1330 | 849 | 282 | 30827.1 | 5.9 | 5 |
| *SmbHLH118* | KP257550 | 770 | 633 | 210 | 24093.6 | 5.5 | 3 |
| *SmbHLH119* | KP257551 | 583 | 471 | 156 | 17457.3 | 9.3 | 2 |
| *SmbHLH120* | KP257552 | 1593 | 882 | 293 | 33416.5 | 7.8 | 7 |
| *SmbHLH121* | KP257553 | 4886 | 1653 | 550 | 60033.6 | 6.2 | 12 |
| *SmbHLH122* | KP257554 | 3519 | 1533 | 510 | 56519.3 | 6.0 | 8 |
| *SmbHLH123* | KP257555 | 2055 | 786 | 261 | 29010.1 | 6.4 | 2 |
| *SmbHLH124* | KP257556 | 754 | 276 | 91 | 10514.8 | 9.0 | 2 |
| *SmbHLH125* | KP257557 | 531 | 282 | 93 | 10322.6 | 9.6 | 2 |
| *SmbHLH126* | KP257558 | 4570 | 714 | 237 | 26122.3 | 9.9 | 5 |
| *SmbHLH127* | KP257559 | 871 | 360 | 119 | 13365.7 | 9.9 | 4 |

**Table S3.** Normal expression sequences of 22 motifs identified in 127 bHLH proteins in *S. miltiorrhiza*

| Motif | Length (aa) | Normal expression sequences |
|---|---|---|
| 1 | 36 | [RK]RGQATD[PS][HQ]SLA[EA]R[AV]RRE[RK]I[SN]E[RK][ML][KR][IA]LQ[DE]LVP[GN][CG]NK |
| 2 | 26 | [MT][DG]KAS[MI]L[DG][ED][AI]I[NE]Y[VLI][KQ][SFE]LQ[LR]Q[VI][KEQ]FL[SE]M |

| | | |
|---|---|---|
| 3 | 26 | H[IL]EAER[RQ]RR[KE][KR][ML]N[DE][RL][LF][AY]ALRS[LV][VL]PNI |
| 4 | 26 | [KR][TRM][DN][KT][AS][SD][IM]LGE[AV][IV]NY[IV]K[EF][LM][QK][KQ][QK][VIL][QE]xLE |
| 5 | 36 | [DE][VI]L[IV][SK]I[CS][CS]xR[RK]PGLL[LS][KS][LVT][VI]AALESLGLD[IV][LVQ][HQ][AC][VN]I[ST]T |
| 6 | 36 | HHQEVPFEGEEELQNLLESPLIQEKLYSTQHCLLPN |
| 7 | 14 | [LK][DAK][EP][LE]P[KP][QK]DYIHVRA |
| 8 | 200 | MVWMDGTKGDTDEHTSSWPQNDDADNNNKDQMEIGALSTFKSMLEAENEDWYITSTGATSAAAPMHDISFSPTFTEAAVNNQLLLQPIDSSASCSPTSAFNNNLDAASQVNYFLQPKPLINHPIQNNALDASFDLGCDGGYLESALNRGGGILNCGFPMGNIGFGLGEGSASSLFNRSKILKPLDHNFGSMGSQPTLFQK |
| 9 | 26 | [AS][IC][AR][EA][RK]QRR[RD]K[IL][NT][VDE]K[FT]QELGKL[LV]P[GP]G[RQ] |
| 10 | 36 | FHFP[KN]RHK[LS]YDYSLPEFVLPPPLPQFPAADFSTRIC |
| 11 | 26 | DSFC[NY]SLLCDDLTYHANGLPL[DE]LDNL |
| 12 | 48 | [IL][DE][GE][YF]PSKD[FL][AS][QG][QP][TA]F[DE][AS][NSP]G[ML][AI][YF]G[SP]Q[ST][TP][RG][EV]Y[AGS]RGS[SQ][PA]EWLHMQ[IV]G[GS]GF[ED]R |
| 13 | 36 | [NS][EN][RG][RG][NS][EC][GN][DN][SN][KN][RN][MV][KS]AVGELKVEGEGNSGKGAAAVER[RC] |
| 14 | 26 | [AH]G[LM]QT[IVL][VA][CLF][IFV]P[TH][DLP][DG]GVVELG[ST][TS][EDR][LC][VI]PE |
| 15 | 26 | MD[KT][AV][SA][VIL]L[DS][ED][TAV]I[EQ][YH][LVI]KEL[KQ][RT][QE]VE[ED]LS[EAK] |
| 16 | 118 | GYLKTHDFLQPLERVGKHVTTTKEDIKAGVAAVDRPPAPAPPASVEHLLPGGIGTYSISYFNQLVLKPEGSLFTAQPTRNDENSNCSSYSGGSFTLWDESAVKKGKTGKENIATERHI |
| 17 | 26 | [YM][YA][SL][NS][WY][TY][ST]FQQPDYSGDDPELQSLLNP |
| 18 | 40 | VYNFAEIWP[AR]FQM[NS]A[TN]A[TA]SYGLGLDPM[VL][MT]DQRSN[HD][SD][PS][PR][NK][HR] |
| 19 | 15 | W[DNST]YAI[FL]W[QKS][LSV]S[RK][SDR][RKQ][SA][GS] |
| 20 | 34 | [DR][EP][NT][VAY][AD][AFL][GS][LP][DE][DEK][VL]TD[AT]E[WM][FY][FY]L[AV][SC]M[SY][FQ][SV]F[NP]R[GT][DE][GQ][GS]P[GP] |
| 21 | 36 | FN[GD]FA[LM][DQ][AIV][FS][RC][AS]E[QD][LWF][GKR][EKQ][GIR][QA][EDIV][LV][DNL][PA][ED][QD]IK[AQ][VA]L[LF][DR][SN][AT]G[FY][HG] |
| 22 | 19 | LAEQVPLLKSNPHLLEKDH |

**Table S4.** RPKM values of 127 *bHLH* genes in various organs

| Gene name | Flower1 | Flower2 | Leaf1 | Leaf2 | Root1 | Root2 | Stem1 | Stem2 |
|---|---|---|---|---|---|---|---|---|
| *SmbHLH1* | 0 | 0.46 | 2.12 | 3.14 | 1.28 | 0.23 | 1.38 | 1.36 |
| *SmbHLH2* | 39.46 | 46.4 | 21.61 | 23.22 | 155.12 | 140.75 | 122.2 | 107.63 |

| | | | | | | | | |
|---|---|---|---|---|---|---|---|---|
| *SmbHLH3* | 0.26 | 0 | 0.44 | 0 | 0 | 0 | 0 | 0 |
| *SmbHLH4* | 81.71 | 86.11 | 16.08 | 10.92 | 16.01 | 13.1 | 38.52 | 26.01 |
| *SmbHLH5* | 0.7 | 1.15 | 0 | 0.41 | 0.64 | 0 | 1.07 | 0.84 |
| *SmbHLH6* | 0.29 | 0 | 0 | 0.13 | 1.97 | 4.86 | 0.53 | 0 |
| *SmbHLH7* | 0 | 0 | 0 | 0 | 0 | 0 | 0 | 0 |
| *SmbHLH8* | 55.9 | 70.21 | 6.22 | 3.82 | 46.4 | 39.21 | 58.46 | 38.5 |
| *SmbHLH9* | 0 | 0 | 0 | 0 | 0 | 0 | 0 | 0 |
| *SmbHLH10* | 0 | 0 | 0 | 0 | 0 | 0 | 0 | 0 |
| *SmbHLH11* | 8.15 | 7.04 | 0 | 1.06 | 2.58 | 0 | 0.59 | 1.13 |
| *SmbHLH12* | 0 | 0 | 0 | 0 | 0.39 | 0.35 | 0 | 0.26 |
| *SmbHLH13* | 10.19 | 7.7 | 21.74 | 63.58 | 78.29 | 136.94 | 59.46 | 77.39 |
| *SmbHLH14* | 29.42 | 26.06 | 7.28 | 6.03 | 9 | 16.17 | 35.89 | 59.34 |
| *SmbHLH15* | 3.35 | 2.44 | 0.17 | 0.17 | 68 | 111.45 | 3.43 | 8.35 |
| *SmbHLH16* | 21.38 | 24.73 | 0 | 0 | 0 | 0 | 0 | 0.1 |
| *SmbHLH17* | 0 | 0.44 | 0 | 0 | 0 | 0 | 0 | 0 |
| *SmbHLH18* | 0 | 0 | 0 | 0 | 0 | 0 | 0 | 0 |
| *SmbHLH19* | 9.61 | 8.22 | 2.51 | 0.85 | 17.68 | 10.14 | 21.97 | 17.65 |
| *SmbHLH20* | 10.32 | 10.32 | 9.78 | 7.04 | 3.47 | 2.46 | 7.2 | 3.68 |
| *SmbHLH21* | 0.79 | 0 | 8.14 | 5.1 | 2.16 | 4.77 | 8.69 | 6.04 |
| *SmbHLH22* | 8.09 | 10.42 | 91.4 | 239.89 | 264.27 | 460.91 | 180.05 | 170.8 |
| *SmbHLH23* | 1.58 | 1.24 | 0 | 0 | 0 | 0 | 0 | 0 |
| *SmbHLH24* | 0.74 | 1.67 | 3.49 | 3.38 | 1.35 | 0.74 | 2.27 | 2.25 |
| *SmbHLH25* | 21.32 | 8.87 | 0.59 | 0.84 | 3.51 | 5.23 | 0.62 | 9.91 |
| *SmbHLH26* | 21.53 | 18.4 | 0.86 | 0.52 | 126.76 | 95.84 | 75.26 | 101.71 |
| *SmbHLH27* | 26.1 | 26.26 | 11.4 | 9.76 | 11.32 | 5.27 | 17.62 | 11.82 |
| *SmbHLH28* | 19.53 | 12.99 | 40.64 | 34.2 | 55.37 | 38.85 | 29.17 | 25.51 |
| *SmbHLH29* | 17.45 | 15.65 | 80.8 | 155.29 | 194.18 | 364.46 | 233.37 | 276.55 |
| *SmbHLH30* | 1.03 | 0.77 | 2.86 | 5.61 | 103.61 | 245.69 | 38.98 | 76.86 |
| *SmbHLH31* | 3.13 | 3.39 | 3.11 | 2.17 | 5.97 | 4.47 | 14.9 | 13.33 |
| *SmbHLH32* | 0 | 0 | 0.29 | 0 | 0 | 0 | 0 | 0 |
| *SmbHLH33* | 5.72 | 3.66 | 4.39 | 2.09 | 12.35 | 13.3 | 12.84 | 9.55 |
| *SmbHLH34* | 0 | 0 | 0 | 0 | 14.87 | 14.59 | 0.31 | 1.08 |
| *SmbHLH35* | 0 | 0 | 0 | 0 | 0 | 0 | 0 | 0 |
| *SmbHLH36* | 0 | 0 | 0 | 0 | 0 | 0 | 0 | 0 |
| *SmbHLH37* | 17.23 | 13.11 | 16.52 | 14.07 | 11.9 | 12.19 | 11.48 | 13.12 |
| *SmbHLH38* | 0.16 | 0.2 | 28.99 | 11.29 | 0 | 0.19 | 0.29 | 0.29 |
| *SmbHLH39* | 4.98 | 5.32 | 17.73 | 15.44 | 0.51 | 0.23 | 30.4 | 17 |
| *SmbHLH40* | 0.36 | 0.89 | 1.1 | 1.11 | 162.03 | 172.81 | 7.44 | 10.24 |
| *SmbHLH41* | 360.5 | 378.56 | 33.87 | 36.34 | 8.15 | 2.55 | 15.5 | 12.71 |
| *SmbHLH42* | 0.11 | 0.14 | 0.1 | 0.2 | 0.15 | 0.27 | 2.37 | 2.03 |
| *SmbHLH43* | 126.22 | 130.8 | 69.93 | 80.52 | 93.9 | 110.61 | 110.47 | 102.79 |
| *SmbHLH44* | 7.87 | 11.35 | 4 | 3.27 | 12.74 | 5.36 | 2.45 | 9.5 |
| *SmbHLH45* | 2.4 | 2.7 | 23.77 | 24.78 | 6.24 | 5.52 | 5.06 | 3.85 |
| *SmbHLH46* | 0 | 0 | 0.82 | 0 | 0 | 0 | 0.3 | 0.27 |

| | | | | | | | | |
|---|---|---|---|---|---|---|---|---|
| *SmbHLH47* | 9.23 | 10.3 | 19.1 | 21.36 | 27.34 | 25.88 | 24.39 | 24.32 |
| *SmbHLH48* | 166.53 | 173.51 | 188.53 | 177.76 | 246.93 | 268.43 | 295.38 | 262.89 |
| *SmbHLH49* | 1.31 | 0 | 0 | 0 | 0 | 0 | 0.61 | 0.59 |
| *SmbHLH50* | 1.01 | 0 | 5.47 | 2.43 | 0 | 0 | 0.23 | 0 |
| *SmbHLH51* | 21.55 | 17.3 | 37.4 | 27.8 | 90.39 | 87 | 26.09 | 51.18 |
| *SmbHLH52* | 15.51 | 14.73 | 17.6 | 15.39 | 16.03 | 14.82 | 14.84 | 11.92 |
| *SmbHLH53* | 13.84 | 9.08 | 5.33 | 16.22 | 98.16 | 124.96 | 40.99 | 67.42 |
| *SmbHLH54* | 0 | 0 | 0 | 0 | 0.15 | 0 | 0 | 0 |
| *SmbHLH55* | 0 | 0 | 0 | 0 | 0 | 0 | 0.35 | 0.34 |
| *SmbHLH56* | 0.41 | 0.17 | 2.24 | 1.08 | 1.48 | 3.27 | 15.65 | 11.86 |
| *SmbHLH57* | 11.78 | 19.84 | 15.4 | 15.13 | 55.16 | 41.43 | 32.84 | 40.83 |
| *SmbHLH58* | 24.62 | 28.5 | 0 | 0 | 0 | 0 | 0 | 0 |
| *SmbHLH59* | 81.95 | 44.83 | 0 | 1.15 | 2.55 | 5.41 | 50.57 | 24.12 |
| *SmbHLH60* | 105.4 | 141.31 | 29.5 | 28.66 | 381.3 | 566.31 | 163.36 | 193.84 |
| *SmbHLH61* | 0 | 0 | 1.45 | 1.2 | 6.05 | 2.62 | 0.79 | 2.53 |
| *SmbHLH62* | 0.14 | 0 | 0 | 0 | 1.53 | 0.51 | 0.64 | 0.51 |
| *SmbHLH63* | 4.73 | 3.89 | 42.33 | 36.22 | 3.87 | 5.32 | 1.88 | 1.99 |
| *SmbHLH64* | 10.46 | 10.83 | 4.68 | 3.8 | 12.1 | 11.63 | 5.18 | 6.25 |
| *SmbHLH65* | 1.46 | 1.5 | 31.23 | 25.56 | 9.28 | 7.47 | 45.23 | 34.54 |
| *SmbHLH66* | 0 | 0 | 0 | 0 | 0 | 1.95 | 6.57 | 14.38 |
| *SmbHLH67* | 9.93 | 8.78 | 0.71 | 0.48 | 7.64 | 7.38 | 13.39 | 17.47 |
| *SmbHLH68* | 26.24 | 27.69 | 8.95 | 9.85 | 36.22 | 13.89 | 65.71 | 88.19 |
| *SmbHLH69* | 0 | 0 | 0 | 0 | 0 | 0 | 0 | 0.89 |
| *SmbHLH70* | 5.8 | 4.77 | 3.7 | 2.5 | 0.53 | 0.78 | 4.01 | 2.09 |
| *SmbHLH71* | 1.41 | 2.35 | 3.23 | 2.83 | 0 | 0 | 0 | 0 |
| *SmbHLH72* | 21.98 | 20.17 | 24.67 | 19.65 | 6.54 | 7.29 | 22.64 | 16.27 |
| *SmbHLH73* | 0.62 | 0.39 | 0.27 | 0.41 | 0 | 0.19 | 0.43 | 0.7 |
| *SmbHLH74* | 5.64 | 1.65 | 3.15 | 5.19 | 46.78 | 37.77 | 1.53 | 1.8 |
| *SmbHLH75* | 1.09 | 1.02 | 0 | 0 | 0.37 | 0 | 0 | 0 |
| *SmbHLH76* | 1.03 | 0.42 | 0 | 0 | 4.22 | 2.07 | 0.31 | 3.09 |
| *SmbHLH77* | 0 | 0 | 0 | 0 | 0 | 0 | 0 | 0 |
| *SmbHLH78* | 39.37 | 24.95 | 4.7 | 3.58 | 12.62 | 16.67 | 1.98 | 1.87 |
| *SmbHLH79* | 0.95 | 1.41 | 0 | 0 | 0 | 0 | 0 | 0 |
| *SmbHLH80* | 92.39 | 87.98 | 40.98 | 36.83 | 93.41 | 53.45 | 68.84 | 58.21 |
| *SmbHLH81* | 46.36 | 20.34 | 2.14 | 1.87 | 7.37 | 7.54 | 9.92 | 10.45 |
| *SmbHLH82* | 4.18 | 2.1 | 0.91 | 0.46 | 1.96 | 4.09 | 4.55 | 4.36 |
| *SmbHLH83* | 61.42 | 62.05 | 13.22 | 12.87 | 29.17 | 32.17 | 39.23 | 37.87 |
| *SmbHLH84* | 37.87 | 43.38 | 4.77 | 4.02 | 1.15 | 0 | 1.64 | 0.68 |
| *SmbHLH85* | 4.94 | 7.98 | 5.44 | 6.34 | 24.62 | 6.19 | 3.31 | 4.97 |
| *SmbHLH86* | 46.78 | 49.04 | 4.69 | 2.9 | 0.1 | 0.18 | 16.93 | 8.13 |
| *SmbHLH87* | 0.27 | 0 | 0 | 0 | 0 | 0 | 0.49 | 0 |
| *SmbHLH88* | 0.49 | 0 | 0 | 0 | 0.33 | 0 | 0.22 | 0 |
| *SmbHLH89* | 11.06 | 5.4 | 0.87 | 1.26 | 16.94 | 15.41 | 15.54 | 13.23 |
| *SmbHLH90* | 0.59 | 0.57 | 3.77 | 3.48 | 0.81 | 0.47 | 0.42 | 0.65 |

| | | | | | | | | |
|---|---|---|---|---|---|---|---|---|
| *SmbHLH91* | 0 | 0 | 0 | 0 | 0 | 0 | 0 | 0 |
| *SmbHLH92* | 0 | 0 | 0 | 0 | 1.93 | 4.28 | 0 | 0 |
| *SmbHLH93* | 36.54 | 51.58 | 0.59 | 0.59 | 62.24 | 71.53 | 7.9 | 14.33 |
| *SmbHLH94* | 17.26 | 14.56 | 25.96 | 19.08 | 51.47 | 30.88 | 10.33 | 12.78 |
| *SmbHLH95* | 0 | 0 | 0 | 0 | 0 | 0 | 0 | 0 |
| *SmbHLH96* | 2 | 1.24 | 1.04 | 0.53 | 0.82 | 2.17 | 7.34 | 6.32 |
| *SmbHLH97* | 5.76 | 3.65 | 1.61 | 1.59 | 0 | 0 | 0 | 0 |
| *SmbHLH98* | 0 | 0 | 0 | 0 | 193.8 | 569.63 | 1.95 | 10.23 |
| *SmbHLH99* | 0.53 | 0.33 | 0 | 0 | 29.19 | 83.51 | 1.21 | 1.43 |
| *SmbHLH100* | 53.72 | 54.53 | 17.9 | 19.64 | 33.6 | 38.39 | 34.29 | 34.66 |
| *SmbHLH101* | 85.35 | 62.45 | 8.63 | 8.2 | 16.82 | 8.4 | 19.51 | 15.97 |
| *SmbHLH102* | 0 | 0 | 0 | 0 | 4.65 | 7.71 | 0 | 0.38 |
| *SmbHLH103* | 0 | 0 | 0.84 | 0.85 | 4.22 | 5.37 | 0 | 0.17 |
| *SmbHLH104* | 0 | 0.8 | 0 | 0 | 0 | 0 | 0 | 0 |
| *SmbHLH105* | 38.65 | 40.26 | 1.67 | 0.38 | 0 | 0 | 6.4 | 5.2 |
| *SmbHLH106* | 0.34 | 0 | 0 | 0 | 0 | 0 | 0 | 0 |
| *SmbHLH107* | 0.51 | 1.01 | 0.18 | 0 | 0.56 | 0.86 | 3.36 | 4.5 |
| *SmbHLH108* | 2.5 | 1.19 | 0.33 | 0.17 | 3.67 | 1.16 | 14.45 | 11.79 |
| *SmbHLH109* | 0.59 | 0.57 | 3.77 | 3.48 | 0.81 | 0.47 | 0.42 | 0.65 |
| *SmbHLH110* | 5.29 | 4.8 | 8.3 | 3.07 | 31.11 | 12.7 | 112.2 | 115.31 |
| *SmbHLH111* | 0 | 0 | 0.84 | 0 | 0 | 0.59 | 0.9 | 0.88 |
| *SmbHLH112* | 1.59 | 1.69 | 15.19 | 17.34 | 0.31 | 0.27 | 20.67 | 15.6 |
| *SmbHLH113* | 0.95 | 0.39 | 17.88 | 23.13 | 0 | 0 | 1.02 | 0.43 |
| *SmbHLH114* | 0 | 0 | 0 | 0.31 | 0 | 0 | 0 | 0 |
| *SmbHLH115* | 0 | 0.48 | 0.51 | 0.17 | 1.59 | 2.11 | 0 | 0.7 |
| *SmbHLH116* | 0 | 0 | 0.48 | 0 | 0 | 0 | 0 | 0 |
| *SmbHLH117* | 4.92 | 3.04 | 0 | 0 | 0 | 0 | 0 | 0 |
| *SmbHLH118* | 2.74 | 3.99 | 0 | 0 | 0 | 0 | 0 | 0 |
| *SmbHLH119* | 0 | 0 | 0 | 0 | 0 | 0 | 0 | 0 |
| *SmbHLH120* | 10.41 | 7.83 | 6.93 | 5.12 | 4.97 | 5.69 | 5.69 | 7.06 |
| *SmbHLH121* | 20.04 | 17.81 | 6.9 | 3.61 | 25.88 | 24.36 | 26.16 | 19.98 |
| *SmbHLH122* | 3.92 | 4.37 | 15.86 | 16.19 | 10.42 | 11.57 | 25.38 | 23.14 |
| *SmbHLH123* | 4.02 | 3.08 | 17.88 | 17.19 | 0.59 | 1.18 | 2.49 | 2.25 |
| *SmbHLH124* | 0 | 0 | 0 | 0 | 0 | 2.64 | 0 | 0 |
| *SmbHLH125* | 0 | 0 | 0 | 0 | 0 | 0 | 1.61 | 2.37 |
| *SmbHLH126* | 0.69 | 0 | 0 | 0 | 7.78 | 3.52 | 0.69 | 0.72 |
| *SmbHLH127* | 28.67 | 30.19 | 19.35 | 13.81 | 0.72 | 0.74 | 10.33 | 3.57 |

**Table S5.** Average RPKM values of 127 *bHLH* genes in various tissues

| Gene name | periderm | phloem | xylem |
|---|---|---|---|
| *SmbHLH1* | 0.250816 | 0.137997 | 0.047584 |
| *SmbHLH2* | 32.178 | 47.4169 | 53.8267 |
| *SmbHLH3* | 0 | 0 | 0 |
| *SmbHLH4* | 14.395 | 10.7774 | 7.11154 |

| | | | |
|---|---|---|---|
| *SmbHLH5* | 56.5685 | 63.1781 | 54.8542 |
| *SmbHLH6* | 18.2229 | 3.5869 | 0.255296 |
| *SmbHLH7* | 0 | 0 | 0 |
| *SmbHLH8* | 14.2532 | 43.8287 | 28.5967 |
| *SmbHLH9* | 0 | 0 | 0 |
| *SmbHLH10* | 0.379571 | 0.45125 | 0.684671 |
| *SmbHLH11* | 0 | 0 | 0 |
| *SmbHLH12* | 18.7707 | 5.43031 | 9.4323 |
| *SmbHLH13* | 14.7268 | 19.3085 | 23.9739 |
| *SmbHLH14* | 4.77885 | 5.42873 | 8.37485 |
| *SmbHLH15* | 5.35023 | 3.12591 | 4.06244 |
| *SmbHLH16* | 0 | 0 | 0 |
| *SmbHLH17* | 0 | 0 | 0 |
| *SmbHLH18* | 0 | 0 | 0 |
| *SmbHLH19* | 1.572 | 2.47373 | 0.69142 |
| *SmbHLH20* | 1.99125 | 2.37782 | 3.14177 |
| *SmbHLH21* | 0.814635 | 0.270925 | 0.294627 |
| *SmbHLH22* | 24.3201 | 48.2763 | 31.2856 |
| *SmbHLH23* | 0 | 0 | 0 |
| *SmbHLH24* | 0.120241 | 0.046024 | 0.34086 |
| *SmbHLH25* | 13.3166 | 12.5834 | 3.03278 |
| *SmbHLH26* | 13.5499 | 23.2158 | 14.4307 |
| *SmbHLH27* | 4.94156 | 3.58277 | 4.34417 |
| *SmbHLH28* | 56.1771 | 34.3278 | 39.4683 |
| *SmbHLH29* | 9.56189 | 20.2728 | 27.4699 |
| *SmbHLH30* | 10.1785 | 1.5877 | 0.661199 |
| *SmbHLH31* | 0.19191 | 2.06858 | 1.31266 |
| *SmbHLH32* | 0 | 0 | 0.084506 |
| *SmbHLH33* | 2.33226 | 3.68129 | 3.69449 |
| *SmbHLH34* | 0.553129 | 0.805356 | 0.520826 |
| *SmbHLH35* | 0 | 0 | 0 |
| *SmbHLH36* | 0 | 0 | 0 |
| *SmbHLH37* | 16.1311 | 17.3671 | 18.9837 |
| *SmbHLH38* | 0.067401 | 0.044682 | 0 |
| *SmbHLH39* | 0.034746 | 0.063796 | 0.130467 |
| *SmbHLH40* | 11.0134 | 30.7627 | 36.464 |
| *SmbHLH41* | 0.784517 | 0.901263 | 0.934977 |
| *SmbHLH42* | 0.180329 | 0.325437 | 0.283512 |
| *SmbHLH43* | 54.3268 | 64.2669 | 67.4107 |
| *SmbHLH44* | 1.42796 | 13.9611 | 11.656 |
| *SmbHLH45* | 4.20752 | 6.70019 | 6.32416 |
| *SmbHLH46* | 0 | 0 | 0.041182 |
| *SmbHLH47* | 42.6189 | 37.9613 | 37.7695 |
| *SmbHLH48* | 118.215 | 140.063 | 114.706 |

| | | | |
|---|---|---|---|
| *SmbHLH49* | 0 | 0 | 0 |
| *SmbHLH50* | 0.057883 | 0.131609 | 0.063847 |
| *SmbHLH51* | 75.5355 | 69.631 | 75.5219 |
| *SmbHLH52* | 16.9012 | 10.627 | 9.24593 |
| *SmbHLH53* | 13.0843 | 20.8803 | 24.3501 |
| *SmbHLH54* | 0 | 0 | 0.005803 |
| *SmbHLH55* | 4.23526 | 0.241854 | 0.098487 |
| *SmbHLH56* | 0.488464 | 0.977531 | 3.33878 |
| *SmbHLH57* | 45.5601 | 36.7096 | 31.507 |
| *SmbHLH58* | 0.0769 | 0 | 0.251613 |
| *SmbHLH59* | 0 | 0.044555 | 0.023503 |
| *SmbHLH60* | 61.7751 | 108.634 | 106.82 |
| *SmbHLH61* | 3.7765 | 5.31943 | 4.00144 |
| *SmbHLH62* | 1.1556 | 1.32227 | 0.530016 |
| *SmbHLH63* | 12.565 | 10.4783 | 9.15835 |
| *SmbHLH64* | 8.64495 | 8.26968 | 9.96751 |
| *SmbHLH65* | 0.582738 | 0.539894 | 6.04038 |
| *SmbHLH66* | 1.55482 | 5.29339 | 4.02166 |
| *SmbHLH67* | 1.01874 | 1.81181 | 0.575405 |
| *SmbHLH68* | 17.7773 | 42.8762 | 51.6843 |
| *SmbHLH69* | 0.052811 | 0 | 0 |
| *SmbHLH70* | 0.270067 | 1.41616 | 1.08235 |
| *SmbHLH71* | 0 | 0.787836 | 0.13912 |
| *SmbHLH72* | 7.87142 | 9.178 | 6.10662 |
| *SmbHLH73* | 0 | 0 | 0 |
| *SmbHLH74* | 189.54 | 91.8689 | 71.9482 |
| *SmbHLH75* | 1.21145 | 1.15065 | 0.308152 |
| *SmbHLH76* | 0.039489 | 0.083629 | 1.30119 |
| *SmbHLH77* | 0 | 0 | 0.024753 |
| *SmbHLH78* | 0.43864 | 9.70756 | 3.21754 |
| *SmbHLH79* | 0 | 0.019143 | 0 |
| *SmbHLH80* | 36.273 | 44.7868 | 33.7649 |
| *SmbHLH81* | 3.45208 | 11.218 | 15.2943 |
| *SmbHLH82* | 0.760464 | 0.768715 | 1.04 |
| *SmbHLH83* | 22.7052 | 23.5549 | 20.8656 |
| *SmbHLH84* | 0.396954 | 0.448977 | 0.398286 |
| *SmbHLH85* | 28.7859 | 39.9442 | 8.35933 |
| *SmbHLH86* | 0 | 0.020107 | 0.031441 |
| *SmbHLH87* | 0 | 0 | 0.034334 |
| *SmbHLH88* | 0 | 0 | 0 |
| *SmbHLH89* | 17.6522 | 16.5592 | 17.769 |
| *SmbHLH90* | 0 | 0 | 0 |
| *SmbHLH91* | 0 | 0 | 0 |
| *SmbHLH92* | 4.01615 | 1.12352 | 1.29232 |

| | | | |
|---|---|---|---|
| *SmbHLH93* | 8.65823 | 6.37651 | 7.20465 |
| *SmbHLH94* | 7.2138 | 16.0075 | 16.1868 |
| *SmbHLH95* | 0 | 0 | 0 |
| *SmbHLH96* | 1.43479 | 0.870781 | 2.46005 |
| *SmbHLH97* | 0 | 0 | 0 |
| *SmbHLH98* | 5.23582 | 6.66513 | 7.65598 |
| *SmbHLH99* | 5.23582 | 6.66513 | 7.65598 |
| *SmbHLH100* | 42.0696 | 40.9834 | 39.6428 |
| *SmbHLH101* | 15.0467 | 13.5355 | 14.3904 |
| *SmbHLH102* | 3.2742 | 1.4283 | 0.261529 |
| *SmbHLH103* | 1.3969 | 0.606834 | 0.322849 |
| *SmbHLH104* | 0 | 0 | 0 |
| *SmbHLH105* | 0 | 0 | 0 |
| *SmbHLH106* | 0 | 0 | 0 |
| *SmbHLH107* | 0.034663 | 0.090716 | 0.020792 |
| *SmbHLH108* | 0.226736 | 0.470946 | 1.53273 |
| *SmbHLH109* | 0 | 0 | 0 |
| *SmbHLH110* | 1.03591 | 4.56445 | 13.6906 |
| *SmbHLH111* | 0 | 0.070317 | 0.268922 |
| *SmbHLH112* | 0.482591 | 0.557549 | 0.059702 |
| *SmbHLH113* | 0 | 0 | 0 |
| *SmbHLH114* | 0 | 0.140976 | 0 |
| *SmbHLH115* | 9.95105 | 3.06414 | 0.993963 |
| *SmbHLH116* | 0 | 0 | 0 |
| *SmbHLH117* | 1.91832 | 0.698791 | 0.047698 |
| *SmbHLH118* | 0 | 0 | 0 |
| *SmbHLH119* | 0 | 0 | 0 |
| *SmbHLH120* | 14.9799 | 54.6215 | 66.1806 |
| *SmbHLH121* | 13.2506 | 12.9863 | 13.912 |
| *SmbHLH122* | 8.64647 | 11.8307 | 9.41128 |
| *SmbHLH123* | 8.96348 | 2.45598 | 1.70543 |
| *SmbHLH124* | 0 | 0 | 0 |
| *SmbHLH125* | 2.72342 | 0.396166 | 0.267471 |
| *SmbHLH126* | 1.68493 | 1.99177 | 2.3213 |
| *SmbHLH127* | 1.14696 | 1.29439 | 0.98035 |

**Table S6.** bHLH binding site prediction for the enzyme-coding genes in the tanshinone biosynthesis pathway

| Gene | E-box | Site |
|---|---|---|
| *DXS1* | Yes | 151(+)CANNTG;305(+)CANNTG;777(+)CANNTG;935(+)CANNTG;1257(+)CANNTG;151(-)CANNTG;305(-)CANNTG;777(-)CANNTG;935(-)CANNTG;1257(-)CANNTG; |
| *DXS2* | Yes | 590(+)CANNTG;1250(+)CANNTG;590(-)CANNTG;1250(-)CANNTG; |

| | | |
|---|---|---|
| *DXS3* | Yes | 60(+)CANNTG;66(+)CANNTG;60(-)CANNTG;66(-)CANNTG; |
| *DXS4* | Yes | 26(+)CANNTG;56(+)CANNTG;99(+)CANNTG;264(+)CANNTG;300(+)CANNTG;26(-)CANNTG;56(-)CANNTG;99(-)CANNTG;264(-)CANNTG;300(-)CANNTG; |
| *DXS5* | Yes | 642(+)CANNTG;1082(+)CANNTG;1134(+)CANNTG;642(-)CANNTG;1082(-)CANNTG;1134(-)CANNTG; |
| *DXS6* | Yes | 276(+)CANNTG;396(+)CANNTG;404(+)CANNTG;787(+)CANNTG;840(+)CANNTG;1135(+)CANNTG;1490(+)CANNTG;276(-)CANNTG;396(-)CANNTG;404(-)CANNTG;787(-)CANNTG;840(-)CANNTG;1135(-)CANNTG;1490(-)CANNTG; |
| *DXS7* | Yes | 5(+)CANNTG;102(+)CANNTG;719(+)CANNTG;1296(+)CANNTG;840(+)CANNTG;1323(+)CANNTG;5(-)CANNTG;102(-)CANNTG;719(-)CANNTG;1296(-)CANNTG;840(-)CANNTG;1323(-)CANNTG; |
| *DXR* | Yes | 849(+)CANNTG;1116(+)CANNTG;849(-)CANNTG;1116(-)CANNTG; |
| *MCT* | Yes | 868(+)CANNTG;1385(+)CANNTG;1460(+)CANNTG;868(-)CANNTG;1385(-)CANNTG;1460(-)CANNTG; |
| *CMK* | Yes | 235(+)CANNTG;515(+)CANNTG;1274(+)CANNTG;235(-)CANNTG;515(-)CANNTG;1274(-)CANNTG; |
| *HDR1* | Yes | 196(+)CANNTG;223(+)CANNTG;527(+)CANNTG;807(+)CANNTG;196(-)CANNTG;223(-)CANNTG;527(-)CANNTG;807(-)CANNTG; |
| *HDR2* | Yes | 119(+)CANNTG;622(+)CANNTG;932(+)CANNTG;1099(+)CANNTG;1360(+)CANNTG;119(-)CANNTG;622(-)CANNTG;932(-)CANNTG;1099(-)CANNTG;1360(-)CANNTG; |
| *HDR3* | Yes | 20(+)CANNTG;175(+)CANNTG;320(+)CANNTG;457(+)CANNTG;805(+)CANNTG;20(-)CANNTG;175(-)CANNTG;320(-)CANNTG;457(-)CANNTG;805(-)CANNTG; |
| *HDR4* | Yes | 472(+)CANNTG;547(+)CANNTG;1017(+)CANNTG;1377(+)CANNTG;1399(+)CANNTG;472(-)CANNTG;547(-)CANNTG;1017(-)CANNTG;1377(-)CANNTG;1399(-)CANNTG; |
| *AACT1* | Yes | 26(+)CANNTG;263(+)CANNTG;983(+)CANNTG;1220(+)CANNTG;26(-)CANNTG;263(-)CANNTG;983(-)CANNTG;1220(-)CANNTG; |
| *AACT2* | Yes | 369(+)CANNTG;817(+)CANNTG;369(-)CANNTG;817(-)CANNTG; |
| *AACT4* | Yes | 313(+)CANNTG;487(+)CANNTG;528(+)CANNTG;1150(+)CANNTG;313(-)CANNTG;487(-)CANNTG;528(-)CANNTG;1150(-)CANNTG; |
| *AACT5* | Yes | 559(+)CANNTG;636(+)CANNTG;655(+)CANNTG;732(+)CANNTG;978(+)CANNTG;559(-)CANNTG;636(-)CANNTG;655(-)CANNTG;732(-)CANNTG;978(-)CANNTG; |

| | | |
|---|---|---|
| *AACT6* | Yes | 944(+)CANNTG;1037(+)CANNTG;1166(+)CANNTG;944(-)CANNTG;1037(-)CANNTG;1166(-)CANNTG; |
| *HMGS1* | Yes | 443(+)CANNTG;528(+)CANNTG;1272(+)CANNTG;443(-)CANNTG;528(-)CANNTG;1272(-)CANNTG; |
| *HMGS2* | No | |
| *HMGR1* | Yes | 155(+)CANNTG;363(+)CANNTG;155(-)CANNTG;363(-)CANNTG; |
| *HMGR2* | Yes | 25(+)CANNTG;385(+)CANNTG;672(+)CANNTG;716(+)CANNTG;25(-)CANNTG;385(-)CANNTG;672(-)CANNTG;716(-)CANNTG; |
| *HMGR3* | Yes | 81(+)CANNTG;279(+)CANNTG;437(+)CANNTG;632(+)CANNTG;867(+)CANNTG;1057(+)CANNTG;1284(+)CANNTG;81(-)CANNTG;279(-)CANNTG;437(-)CANNTG;632(-)CANNTG;867(-)CANNTG;1057(-)CANNTG;1284(-)CANNTG; |
| *HMGR4* | Yes | 552(+)CANNTG;569(+)CANNTG;1055(+)CANNTG;1343(+)CANNTG;552(-)CANNTG;569(-)CANNTG;1055(-)CANNTG;1343(-)CANNTG; |
| *HMGR5* | Yes | 115(+)CANNTG;129(+)CANNTG;283(+)CANNTG;481(+)CANNTG;639(+)CANNTG;834(+)CANNTG;1069(+)CANNTG;1259(+)CANNTG;1486(+)CANNTG;115(-)CANNTG;129(-)CANNTG;283(-)CANNTG;481(-)CANNTG;639(-)CANNTG;834(-)CANNTG;1069(-)CANNTG;1259(-)CANNTG;1486(-)CANNTG; |
| *MK* | Yes | 150(+)CANNTG;961(+)CANNTG;150(-)CANNTG;961(-)CANNTG; |
| *PMK* | Yes | 8(+)CANNTG;69(+)CANNTG;115(+)CANNTG;168(+)CANNTG;539(+)CANNTG;919(+)CANNTG;1425(+)CANNTG;1435(+)CANNTG;8(-)CANNTG;69(-)CANNTG;115(-)CANNTG;168(-)CANNTG;539(-)CANNTG;919(-)CANNTG;1425(-)CANNTG;1435(-)CANNTG; |
| *MDC1* | Yes | 336(+)CANNTG;793(+)CANNTG;883(+)CANNTG;1139(+)CANNTG;1390(+)CANNTG;1417(+)CANNTG;336(-)CANNTG;793(-)CANNTG;883(-)CANNTG;1139(-)CANNTG;1390(-)CANNTG;1417(-)CANNTG; |
| *MDC2* | Yes | 22(+)CANNTG;241(+)CANNTG;773(+)CANNTG;865(+)CANNTG;1140(+)CANNTG;1391(+)CANNTG;1418(+)CANNTG;22(-)CANNTG;241(-)CANNTG;773(-)CANNTG;865(-)CANNTG;1140(-)CANNTG;1391(-)CANNTG; 1418(-)CANNTG; |
| *MDC3* | Yes | 64(+)CANNTG;275(+)CANNTG;876(+)CANNTG;1139(+)CANNTG;1390(+)CANNTG;1417(+)CANNTG;64(-)CANNTG;275(-)CANNTG;876(-)CANNTG;1139(-)CANNTG;1390(-)CANNTG;1417(-)CANNTG; |
| *IDI1* | Yes | 50(+)CANNTG;246(+)CANNTG;682(+)CANNTG;1394(+)CANNTG;50(-)CANNTG;246(-)CANNTG;682(-)CANNTG;1394( |

| | | |
|---|---|---|
| | | -)CANNTG; |
| *IDI3* | Yes | 1(+)CANNTG;78(+)CANNTG;340(+)CANNTG;351(+)CANNTG;377(+)CANNTG;519(+)CANNTG;730(+)CANNTG;1182(+)CANNTG;1223(+)CANNTG;1290(+)CANNTG;1403(+)CANNTG;1450(+)CANNTG;1(-)CANNTG;78(-)CANNTG;340(-)CANNTG;351(-)CANNTG;377(-)CANNTG;519(-)CANNTG;730(-)CANNTG;1182(-)CANNTG;1223(-)CANNTG;1290(-)CANNTG;1403(-)CANNTG;1450(-)CANNTG; |
| *IDI4* | Yes | 235(+)CANNTG;265(+)CANNTG;476(+)CANNTG;1236(+)CANNTG;1349(+)CANNTG;1400(+)CANNTG;1449(+)CANNTG;235(-)CANNTG;265(-)CANNTG;476(-)CANNTG;1236(-)CANNTG;1349(-)CANNTG;1400(-)CANNTG;1449(-)CANNTG; |
| *IDI5* | Yes | 53(+)CANNTG;166(+)CANNTG;213(+)CANNTG;53(-)CANNTG;166(-)CANNTG;213(-)CANNTG; |
| *GGPPS1* | Yes | 315(+)CANNTG;538(+)CANNTG;1123(+)CANNTG;315(-)CANNTG;538(-)CANNTG;1123(-)CANNTG; |
| *GGPPS2* | Yes | 247(+)CANNTG;361(+)CANNTG;479(+)CANNTG;1037(+)CANNTG;1404(+)CANNTG;1440(+)CANNTG;247(-)CANNTG;361(-)CANNTG;479(-)CANNTG;1037(-)CANNTG;1404(-)CANNTG;1440(-)CANNTG; |
| *GGPPS3* | Yes | 996(+)CANNTG;996(-)CANNTG; |
| *GGPPS4* | Yes | 247(+)CANNTG;361(+)CANNTG;479(+)CANNTG;1037(+)CANNTG;1404(+)CANNTG;1440(+)CANNTG;247(-)CANNTG;361(-)CANNTG;479(-)CANNTG;1037(-)CANNTG;1404(-)CANNTG;1440(-)CANNTG; |
| *GGPPS5* | Yes | 844(+)CANNTG;844(-)CANNTG; |
| *GPPS.LSU1* | Yes | 256(+)CANNTG;459(+)CANNTG;507(+)CANNTG;1220(+)CANNTG;1367(+)CANNTG;256(-)CANNTG;459(-)CANNTG;507(-)CANNTG;1220(-)CANNTG;1367(-)CANNTG; |
| *GPPS.LSU2* | Yes | 9(+)CANNTG;458(+)CANNTG;563(+)CANNTG;1226(+)CANNTG;9(-)CANNTG;458(-)CANNTG;563(-)CANNTG;1226(-)CANNTG; |
| *GPPS.LSU3* | Yes | 271(+)CANNTG;376(+)CANNTG;1039(+)CANNTG;1486(+)CANNTG;271(-)CANNTG;376(-)CANNTG;1039(-)CANNTG;1486(-)CANNTG; |
| *GPPS.LSU5* | Yes | 1173(+)CANNTG;1301(+)CANNTG;1366(+)CANNTG;1173(-)CANNTG;1301(-)CANNTG;1366(-)CANNTG; |
| *GPPS.LSU6* | Yes | 24(+)CANNTG;233(+)CANNTG;24(-)CANNTG;233(-)CANNTG; |
| *GPPS.SSUI.1* | Yes | 176(+)CANNTG;856(+)CANNTG;176(-)CANNTG;856(-)CANNTG; |
| *GPPS.SSUI.2* | Yes | 839(+)CANNTG;882(+)CANNTG;839(-)CANNTG;882(-)CANNTG; |

| | | |
|---|---|---|
| *GPPS.SSUII.1* | Yes | 486(+)CANNTG;511(+)CANNTG;780(+)CANNTG;793(+)CANNTG;1415(+)CANNTG;1576(+)CANNTG;486(-)CANNTG;511(-)CANNTG;780(-)CANNTG;793(-)CANNTG;1415(-)CANNTG;1576(-)CANNTG; |
| *GPPS.SSUII.2* | Yes | 446(+)CANNTG;502(+)CANNTG;916(+)CANNTG;1266(+)CANNTG;1293(+)CANNTG;1403(+)CANNTG;446(-)CANNTG;502(-)CANNTG;916(-)CANNTG;1266(-)CANNTG;1293(-)CANNTG;1403(-)CANNTG; |
| *GPPS.SSUII.3* | Yes | 28(+)CANNTG;118(+)CANNTG;264(+)CANNTG;436(+)CANNTG;539(+)CANNTG;630(+)CANNTG;832(+)CANNTG;1190(+)CANNTG;1313(+)CANNTG;1348(+)CANNTG;1440(+)CANNTG;28(-)CANNTG;118(-)CANNTG;264(-)CANNTG;436(-)CANNTG;539(-)CANNTG;630(-)CANNTG;832(-)CANNTG;1190(-)CANNTG;1313(-)CANNTG;1348(-)CANNTG;1440(-)CANNTG; |
| *CPS1* | Yes | 303(+)CANNTG;503(+)CANNTG;512(+)CANNTG;555(+)CANNTG;641(+)CANNTG;739(+)CANNTG;1101(+)CANNTG;1361(+)CANNTG;303(-)CANNTG;503(-)CANNTG;512(-)CANNTG;555(-)CANNTG;641(-)CANNTG;739(-)CANNTG;1101(-)CANNTG;1361(-)CANNTG; |
| *CPS2* | No | |
| *CPS3* | Yes | 36(+)CANNTG;66(+)CANNTG;138(+)CANNTG;917(+)CANNTG;36(-)CANNTG;66(-)CANNTG;138(-)CANNTG;917(-)CANNTG; |
| *CPS4* | Yes | 417(+)CANNTG;895(+)CANNTG;1026(+)CANNTG;1369(+)CANNTG;417(-)CANNTG;895(-)CANNTG;1026(-)CANNTG;1369(-)CANNTG; |
| *CPS5* | Yes | 284(+)CANNTG;849(+)CANNTG;860(+)CANNTG;1152(+)CANNTG;284(-)CANNTG;849(-)CANNTG;860(-)CANNTG;1152(-)CANNTG; |
| *CPS6* | Yes | 187(+)CANNTG;387(+)CANNTG;607(+)CANNTG;1148(+)CANNTG;187(-)CANNTG;387(-)CANNTG;607(-)CANNTG;1148(-)CANNTG; |
| *CPS7* | Yes | 216(+)CANNTG;822(+)CANNTG;853(+)CANNTG;1207(+)CANNTG;216(-)CANNTG;822(-)CANNTG;853(-)CANNTG;1207(-)CANNTG; |
| *CPS9* | Yes | 552(+)CANNTG;591(+)CANNTG;792(+)CANNTG;552(-)CANNTG;591(-)CANNTG;792(-)CANNTG; |
| *CPS10* | Yes | 264(+)CANNTG;555(+)CANNTG;1079(+)CANNTG;1311(+)CANNTG;264(-)CANNTG;555(-)CANNTG;1079(-)CANNTG;1311(-)CANNTG; |
| *CPS11* | Yes | 483(+)CANNTG;502(+)CANNTG;1325(+)CANNTG;1336(+)CANNTG;483(-)CANNTG;502(-)CANNTG;1325(-)CANNTG;1336(-)CANNTG; |

| | | |
|---|---|---|
| *CPS12* | Yes | 582(+)CANNTG;601(+)CANNTG;915(+)CANNTG;582(-)CANNTG;601(-)CANNTG;915(-)CANNTG; |
| *CPS13* | Yes | 582(+)CANNTG;601(+)CANNTG;915(+)CANNTG;582(-)CANNTG;601(-)CANNTG;915(-)CANNTG; |
| *KSL1* | Yes | 183(+)CANNTG;378(+)CANNTG;485(+)CANNTG;501(+)CANNTG;664(+)CANNTG;749(+)CANNTG; 1252(+)CANNTG; 183(-)CANNTG;378(-)CANNTG;485(-)CANNTG;501(-)CANNTG;664(-)CANNTG;749(-)CANNTG; 1252(-)CANNTG; |
| *KSL2* | Yes | 140(+)CANNTG;1005(+)CANNTG;1212(+)CANNTG;1241(+)CANNTG;1420(+)CANNTG;140(-)CANNTG;1005(-)CANNTG;1212(-)CANNTG;1241(-)CANNTG;1420(-)CANNTG; |
| *KSL3* | Yes | 113(+)CANNTG;113(-)CANNTG; |
| *KSL4* | Yes | 332(+)CANNTG;835(+)CANNTG;332(-)CANNTG;835(-)CANNTG; |
| *KSL5* | Yes | 258(+)CANNTG;264(+)CANNTG;282(+)CANNTG;603(+)CANNTG;765(+)CANNTG;1031(+)CANNTG;258(-)CANNTG;264(-)CANNTG;282(-)CANNTG;603(-)CANNTG;765(-)CANNTG;1031(-)CANNTG; |
| *KSL7* | Yes | 377(+)CANNTG;394(+)CANNTG;464(+)CANNTG;1330(+)CANNTG;377(-)CANNTG;394(-)CANNTG;464(-)CANNTG;1330(-)CANNTG; |
| *KSL8* | Yes | 378(+)CANNTG;395(+)CANNTG;465(+)CANNTG;1330(+)CANNTG;378(-)CANNTG;395(-)CANNTG;465(-)CANNTG;1330(-)CANNTG; |
| *KSL9* | Yes | 1206(+)CANNTG;1261(+)CANNTG;1206(-)CANNTG;1161(-)CANNTG; |
| *MDS* | Yes | 1193(+)CANNTG;1228(+)CANNTG;1281(+)CANNTG;1291(+)CANNTG;1395(+)CANNTG;1193(-)CANNTG;1228(-)CANNTG;1281(-)CANNTG;1291(-)CANNTG;1395(-)CANNTG; |
| *CYP76AH1* | Yes | 980(+)CANNTG;1257(+)CANNT;1359(+)CANNTG;980(-)CANNTG;1257(-)CANNT;1359(-)CANNTG |

**Table S7.** bHLH transcription factors related to active compound biosynthesis in 21 plant species

| **Species** | **Gene** | **Active compound** |
|---|---|---|
| *Zea mays* L. | *Lc* | anthocyanin |
| | *ZmR/B* | purple anthocyanin |
| *Arabidopsis thaliana* L. | *AtTT8* | flavonoid |
| | *AtEGL3* | flavonoid |
| | *AtGL3* | anthocyanin |
| | *AtMYC1* | anthocyanin |

| | | |
|---|---|---|
| | *MYC-146* | anthocyanin |
| | *JAM1/2/3* | anthocyanin |
| *Petunia hybrida* Vilm | *PhAN1* | anthocyanin |
| | *JAF13* | anthocyanin |
| *Nicotiana tabacum* L. | *NtAn1/2* | anthocyanin |
| | *OSB1/OSB2* | anthocyanin |
| | *Ra/Rb* | anthocyanin |
| | *OsbHLH14* | anthocyanin |
| | *Rc* | paoanthocyanidin |
| *Ipomoea purpurea* (L.) Roth. | *IpIVS* | anthocyanin, proanthocyanidin, tannin |
| *Ipomoea tricolor* Cav. | *ItIVS* | proanthocyanidin, phytomelanin |
| *Vitis vinfera* L. | *VvMYC1* | anthocyanin, tannin |
| *Malus* × *domestica* Borkh. | *MdbHLH3/33* | anthocyanin |
| *Brassica oleracea* L. var. *capitata* | *BoTT8* | anthocyanin |
| *Gerbera hybrida* Hort. | *GhMYC1* | anthocyanin |
| *Perilla frutescens* Britton. var. *crispa* | *MYC-RP/GP* | anthocyanin |
| *Lilium* × *elegans* Thunb. | *LhbHLH2* | anthocyanin |
| *Dahlia variabilis* Hort. | *DvIVS* | anthocyanin |
| *Diospyros kaki* Thunb. | *DkMYC1* | paoanthocyanidin |
| *Gentiana triflora* Pall | *GtbHLH1* | polyacylated anthocyanin |
| *Antirrhinum majus* L. | *Delila* | red anthocyanin |
| *Nicotiana tabacum* L. | *NtMYC2* | nicotine |
| | *MYC2a/2b* | nicotine |
| *Nicotiana benthamiana* L. | *NbbHLH1/2* | nicotine |
| *Coptis japonica* Makino | *CjbHLH1* | isoquinoline alkaloid |

| | | |
|---|---|---|
| *Catharanthus roseus* (L.) G. Don | *CrMYC2* | terpenoid indole alkaloid |
| *Taxus cuspidata* S. et. Z. | *TcJAMYC* | paclitaxel |

**Table S8.** Amino acid sequences of the bHLH domain in *S. miltiorrhiza*

| Gene name | Amino acid sequences of the bHLH domain |
|---|---|
| *SmbHLH1* | NHSLAERVRRERISERMRLLQELVPGCDKITGKAVVLDEIINYVQSLQ |
| *SmbHLH2* | HSIAERLRRERIAERIRALQDLVPSKTDRAAMLDEIVDYVKFL |
| *SmbHLH3* | VSHITVERNRRKQMNEHLSVLRSLMPCFYVKRGDQASIIGGVVDYINE LQ |
| *SmbHLH4* | SHSLAERARREKINARMKLLQDLVPGCNKISGTAMVLDEIINHVQALQ |
| *SmbHLH5* | IAARQRRRKITLKTQELGKLVPGQRMNTAEMLQSAYNYIKFLQ |
| *SmbHLH6* | DHILAERKRREKLSQRFIALSALVPGLKKMDKASVLGDAIKYMKQLQ |
| *SmbHLH7* | IAARQRRRKITVKTQELGKLVPGQRMNTAEMLQSAYNYIKFLQ |
| *SmbHLH8* | AHSLAERVRREKISERMKALQDLVPGCKVTGKAVMLDEIINYVQSLQ |
| *SmbHLH9* | RQRRRKITVKTQELGKLVPGQRMNTAEMLQSAYNYIKFLQ |
| *SmbHLH10* | SHSLAERARREKISERMKILQDLVPG |
| *SmbHLH11* | SHSLAERARREKISERMKILQDLVPG |
| *SmbHLH12* | KTAHREIERQRRQEMSTLYASLRELLPLkvyicffpfkyyfkealnqSTWQGKRS VSDHMHEAASYIKD |
| *SmbHLH13* | LQNKLADLVErpnssNFCWNYAIFWQLSRSKAgdlVLGWGDGCCREPRD DEESEvtrilKmrledeSQQTMRKRVLQRLHTLFGggdeeNYAFGLDKVTDTE MFFLASMYFSFprGEGGPGRCFGSGKYVWLSDSLKSSVDYCVRSFLAK SAGMQTIVLIPTDVGVVELGSVRCIPESMELVKVVGSSF |
| *SmbHLH14* | SHSLAERVRRERISERMKLLQALVPGCDKALMLDEIINYVQSLQ |
| *SmbHLH15* | QDHLMAERKRREDLRHLFIALSKVVPGLKKLDKASLLEDAINHLKSLE |
| *SmbHLH16* | VLysLCWLPGKAYVDRQHIWLTRANKADSTLFSRTILAESAKIQTVVCIP LLDGVVELGTTQRVEEDIGLIQRVKSFF |
| *SmbHLH17* | SEKRRRVELKGKYESLRLLIPSPSKNDKASVLADAIRYIKEL |
| *SmbHLH18* | SEKRRRVELKGKYESLRLLVPDPSKNDRASIVADAIRYIKEL |
| *SmbHLH19* | HSIAERLRRERIAERMKALQELVPNANRTDKASMLDEIIDYVKFL |
| *SmbHLH20* | MAERRRRKKLNDRLYMLRSVVPKiSKMDRASILGDAIDYLKEL |
| *SmbHLH21* | ARHRRERISERIRILQRLVPGGTKMDTASMLDEAIHYVKFL |
| *SmbHLH22* | VSERNRRRKLNERLYALRAVVPNITKMDKASIIRDAIEYIKSLQ |
| *SmbHLH23* | RRRRKRLNDRLSMLRSVVPRiSKMDRASILGDTIDYMREL |
| *SmbHLH24* | KSHSEAERRRRERINNHLAKLRSLLPSTTKTDKASLLAEVIQHVKEL |
| *SmbHLH25* | LMAERRRRKRLNDRLSMLRSVVPKiSKMDRTSILGDTIDYMKEL |
| *SmbHLH26* | SHSLAERVRREKISERMKLLQDLVPGCNKVTGKALMLDEIINYVQSLQ |
| *SmbHLH27* | SHSLAERARREKINARMKLLQELVPGCNKISGTAMVLDEIINHVQALQ |
| *SmbHLH28* | SIAERERRTRISDRIRKLQELVPNMDKQTNTADMLEEAVAYVKHLQ |
| *SmbHLH29* | LQKKLHHILQtqAEWWAYAILWQTSKDESgriVLTWADGHFQGTKQKNP PSGSLQPERKKVMRGIQALIGeGVDPLEGEVTDAEWFYVMSLAQSIslG DGVVGKAFNSGSLVWLTGANQLRFYNCHRAKEAQIHGMQTMVCIPTF |

DGVLELGSDLIVAENWNLVQQAKSLF

| | |
|---|---|
| *SmbHLH30* | KKTAHRFTEKQRRQEMTTLYASLRSLLPLeYIKGKRAVSDHMDQAANYINDM |
| *SmbHLH31* | QLALAVRSIQWSYAIFWSVSSRQPgMLEWCEGYYNGDIKTRKTVqaaEvnmdQLGLQRSDQLRELYESLSlgetkpqakrpTAALSPEDLTDAEWYFLVCMSFLFnttDQGLPARTLATNQMIWLCNAHRADTKFFSRSLLAKTIVCFPHLGGVVELGTTELVPEDPNLIRHISSF |
| *SmbHLH32* | QRMTHIAVERNRRKQMNEHLQILRSLMPTYYVHRGDQASIVGAAIEFVRELQ |
| *SmbHLH33* | NSHSLAERVRRERISERMRLLQELVPGCNKITGKAMMLDEIINYVQSLQ |
| *SmbHLH34* | KIVHREIERQRRKEMADLHASLRSVLPSkCIKGTRSASDQIHEATKYIRYMQ |
| *SmbHLH35* | SERKRPEKLNDSFQILRSFLPPGTKKDKASVLSNTTEYLSSL |
| *SmbHLH36* | SHSLAERARREKISERMKILQDLVPGC |
| *SmbHLH37* | QEALCKIVEGSDLTYAIYWHVSKSKSgrsALIWGDGHYQESKESVHDScgsyNdlkrVEGDRRKWVLQKLHACFGgleddNVAAKLDQVSNVEMLYLTSMFFVFpfdKPSIPSQSFNSDRCIWVSNLDSCLERYHSRAYLAKLAQFETVAFVPTKSGVVEIGSRKSIPENKSIIKAAKSI |
| *SmbHLH38* | ARQRRERISERIRVLQKLVPGGSKMDTASMLDEAANYLKFL |
| *SmbHLH39* | HNQSERKRRDKINQRMKTLQKLVPNSSKSDKASMLDEVIEYLKQLQ |
| *SmbHLH40* | QRMTHIAVERNRRRQMNDYLAVLRSIMPPSYTQRGDQASIVGGAINFVKELE |
| *SmbHLH41* | SHSLAERARREKISERMKILQDLVPGCNKVIGKALVLDEIINYIQSLQ |
| *SmbHLH42* | HMISERKRREKLNDSFQILRSFLPPGSKKDKASVLSSTTEYLSSL |
| *SmbHLH43* | LQQRLLSLIEdaaHESWTYAIFWQSSVRRCSAGDGYYKGEEDKAKRRtaTspAEQEHRKKVLRDLNSLIAgpqavadeAVDAVDEEVTDTEWFFLISMTQNFvnGSGLPGQALYTSSPVWVTGPERLASSHCDRARQAQGFGLQTLVCIPSPNGVVELGSTEL |
| *SmbHLH44* | LMAERRRRKRLNDRLSMLRSIVPKiSKMDRTSILGDTIDYMREL |
| *SmbHLH45* | SIAERVRRTRISERMRKLQELVPNMDKQTNTSDMLDLAVEYIKALQ |
| *SmbHLH46* | KSHSEAERRRRERINNHLAKLRSLLPSTTKTDKASLLAEVIQHVKEL |
| *SmbHLH47* | SIAERVRRTRISEKMKKLQDLFPNMDKQTSTADMLDLAVQYIKDLQ |
| *SmbHLH48* | SKACREKQRRDRLNDKFMELGALLEPGRPPKTDKAAILVDAVRMVTQL |
| *SmbHLH49* | SHSLAERARREKISERMKILQDLVPGC |
| *SmbHLH50* | SHIAVERNRRRQMNEHLKVLRSLTPCFYIKRGDQASIIGGVIEFIKEL |
| *SmbHLH51* | LRKQLALAVRSTQWSYAIFWSFSAKQSGDGYYNGDIKTRKTVqtvElnsdPLGLQRSDQLRELFESLSlgettpqpkrpTAALSPEDLTDTEWYFLVCMSFVFNKTRQSGYAMLILQTRSSSLVLCLLRQVASLQTVVCFPHWGGVVELGTTELVPEDRSLIEHIKTSF |
| *SmbHLH52* | SIAERVRRTKISERMRKLQELVPNMEKQTNTSDMLDLAVDYIKDLQ |
| *SmbHLH53* | LQNKLSDLVErpnsaNFSWNYAIFWQLSRSRAgdlVLGWGDGCCREPREIEEDSdvtrflRirqedeCQQRMRKRVLQKLHISFGgsedeSYAFGLDKVTDAEMFFLASMYFSYarGEGGPGRCFSSGEHLWLSDAFKSPVDYCVRSFLARSAG |

| | |
|---|---|
| | VQTIVLVPTDIGVVELGSIRCVPESLELVKVVRSSF |
| *SmbHLH54* | SIAARQRRRKITVKTQELGKLVPGgHRMNTAEMLQSAYNYVKFLQ |
| *SmbHLH55* | RSTTLMSERRRRRRMKEKLYTLRSLVPNITKMDKASIVGDAVLYVQEL<br>Q |
| *SmbHLH56* | SHKEAERRRRKRINGHIATLKSMLPNTIKTDKASLLGEAVRRVKEL |
| *SmbHLH57* | KVLKADREKLRRDKLNEQFLELGNLLDPDRPKNDKGTIVSDTIQVLKE<br>L |
| *SmbHLH58* | ARHRRERISEKIRILQRLVPGGTKMDTASMLDEAIRYVKFL |
| *SmbHLH59* | LHTQLQFILQsqTERWDYAIFWKSWRGVDgggrpVLSWGSGYFHGDTVT<br>VPTNTKPDPSESEWFYMASITRSFaaTDDLVFRAHATASNVWLVGPQQL<br>NLCGSERAKEALLHGLTTLAFIPTPYGVVELGSSDLIKENWSLIKLVTNS<br>F |
| *SmbHLH60* | SIAERNRRTRISKNMKKLQDLFPNMDKQTNTADMLDLAVDYIKEL |
| *SmbHLH61* | SIAERERRTRISGKLRKLQDLVPNMDKQTSYADMLDLAVQHIKTLQ |
| *SmbHLH62* | RKRRERINERLKILQTLVPNGTKVDISTMLEEAVEYVKFLQ |
| *SmbHLH63* | VHNMSERRRRDRINEKMKALQELIPHANKSDKASMLDEAIEYMKALQ |
| *SmbHLH64* | DRFQILRDLIPENDQKRDKASFLLEVIQYIQFLQ |
| *SmbHLH65* | KSHSEAERRRRERINNHLAKLRSLLPNTTKTDKASLLAEVIQHVKEL |
| *SmbHLH66* | ERKIIEKNRRNKMKNLYSQLVSHLPPqPSslvegaplpdqidqvvehiKNMKTKL<br>EK |
| *SmbHLH67* | RRERIAERMKALQELVPNANRTDKASMLDEIIDYVKFL |
| *SmbHLH68* | RSHSEAEKRRRDRINAQLSTLRKLIPKSEKMDKAALLGHVVDHVKE |
| *SmbHLH69* | VSERRRRGRMKEKLYALRSLVPNITKMDKASIVGDAVLYVQDLQ |
| *SmbHLH70* | VHNLSEKRRRSRINEKLKALQNLIPNSNKTDKASMLDEAIEYLKQLQ |
| *SmbHLH71* | DEINDLILKLQPLLPDsRSNTRVSASMILKETCNYLKKL |
| *SmbHLH72* | VHNLSERRRRDRINEKMRALQELIPNCNKVDKASMLDEAIEYLKTLQ |
| *SmbHLH73* | RKRRERINERLRILQNLVPNGTKVDISTMLEEAVQYVKFLQ |
| *SmbHLH74* | AKACRERERRGKLNERFLELSAILGRERPFKTEKLAMLSDAIRLLNQL |
| *SmbHLH75* | DHSLAERKRREKIGKRFAALSALVPGLKKMDKASILEDAIEYMKLL |
| *SmbHLH76* | HILTERERRKKMRDMFSNLHALLPHiPPKADKSTIVDEAVKYIKR |
| *SmbHLH77* | HILTERERRKKMRDMFSNLHALLPHiPPKRKe |
| *SmbHLH78* | HSIAERLRRERIAERMKALQELVPNANKTDKASMLDEIIDYVKFLQ |
| *SmbHLH79* | RRRRERINERLKILQNLVPNGTKVDISTMLEEAVQYVKFLQ |
| *SmbHLH80* | KACRERMRREKLNDRFTELSDTLEPGRPAKTDKLAILGDAIRVLNQL |
| *SmbHLH81* | SIAARQRRRKITVKTQELGKLVPGgHRMNTAEMLQSAYNYVKFLQ |
| *SmbHLH82* | VHNLSEKRRRSRINEKMKALQNLIPNSNKTDKASMLDEAIEYLKQLQ |
| *SmbHLH83* | RRVQKADREKLRRDRLNEQFMELGNALDPDRPKNDKASILSDTIQML<br>KDL |
| *SmbHLH84* | SHSLAERARREKISERMKILQDLVPGCNKVIGKALVLDEIINYIQSLQ |
| *SmbHLH85* | KKLNHNASERDRRKKMNTLYANLRSLLPPeDHSKKLSIPATISRVLKYIP<br>ELQ |
| *SmbHLH86* | SHSLAERARREKISERMRILQDLVPGCNKVIGKALVLDEIINYIQSLQ |
| *SmbHLH87* | TERERRKKMRNMFATLHSLIPHLHPRADKSSIVDEAVIHIKNMQ |
| *SmbHLH88* | RKRRERINERLRILQNLVPNGTKVDISTMLEEAVQYVKFLQ |

| | |
|---|---|
| *SmbHLH89* | RSKHSATEQRRRSKINDRHRLREIIPNSEQKRDKASFLLEVIEYIQFLQ |
| *SmbHLH90* | MAERRRRKKLNDRLYMLRSVVPKiSKMDRASILGDAIDYLKEL |
| *SmbHLH91* | ERLRPLVGIKGWDYIVLWKLSDDRRSIELMDCCCAGGDNADELGFEASSSTLPCRDVMYPHPRLKSCDLLDLIPSSMVLDSGVHAQTLCSNQARWLNYSHSSDSSLSSQDDIGTRVLIPLSVGLVELFVNNQVGEDEGVVDLIR |
| *SmbHLH92* | KKTAHRFTERQRRQEMSALYASLRSLLPLqYVKGKRAVSDHMHQAVNYVND |
| *SmbHLH93* | RRVLHRDIERKRREEMSKLYVSLRTLLPMeHNKGKSSVSDQMEEATKYIKQM |
| *SmbHLH94* | QRMTHIAVERNRRKQMNEHLAVLRSLMPDSYVQRGDQASIVGGAIEFVKELE |
| *SmbHLH95* | AARQRRHRISDRFKILQSLVPGGSKMDTVSMLEQAIQYVKFL |
| *SmbHLH96* | SHSLAERVRRGKINERLRCLRDIVPGCYKTMGMAVMLDEIINYVQSLQ |
| *SmbHLH97* | DELNNLIFKLHASLPHhSNSTSNKKTSAAKILKRTCNYIKKLQ |
| *SmbHLH98* | KKTAHRFTEKQRRQEMAALYASLRSLLPLeYIKGKRAISDHMHQAVNYINDM |
| *SmbHLH99* | KKTAHKFIEKQRRQEMTALYASLRSLLPLqYVKGKRAVSDHMHQAVNYVND |
| *SmbHLH100* | RSKHSVTEQRRRSKINERFQILRELIPHSDQKRDTASFLLEVIQYVQFLQ |
| *SmbHLH101* | SHSLAERARREKISERMKILQDLVPGCNKVIGKALVLDEIINYIQSLQ |
| *SmbHLH102* | KKTAHRFTERQRRQEMSGLYASLRSLLPLqYVKGKRAVSDHMHQAVNYVNDM |
| *SmbHLH103* | KKVMHRELERKRRQEMSHLYASLSSLLPHhQIKGKHGVCDQIQEAASHIKKM |
| *SmbHLH104* | SIAARQRRRKITVKTQELGKLVPGGQRMNTAEMLQSAYNYIKFLQ |
| *SmbHLH105* | SHSLAERARREKISERMRILQDLVPGCNKVSICKALVLDEIINYIQSLQ |
| *SmbHLH106* | SHSLAERARREKISERMKILQDLVPGC |
| *SmbHLH107* | EMHNLSERRRRDRINQKMHALQELIPNCNKADKASMLDEAIEYVKNL |
| *SmbHLH108* | RERAYATDRIRKLRISRWLDALQELVPSPERGGQAALLDGVIDYIKYLQ |
| *SmbHLH109* | MAERRRRKKLNDRLYMLRSVVPKiSKMDRASILGDAIDYLKEL |
| *SmbHLH110* | SHSLAERVRREKISERMKYLQDLVPGCNKVTGKAGMLDEIINYVQSLQ |
| *SmbHLH111* | AARQRRHRISDRFKILQSLVPGGSKMDTVSMLEQAIQYVKFL |
| *SmbHLH112* | RADRKTIEKNRRNEMKALYTNLNSLIPPqPHSRPREMVSLPDQLEAATNYIKMQQ |
| *SmbHLH113* | QRMTHIAVERNRRKQMNEHLRVLRSLMPSSYVQRGDQASIIGGAIEFVRELE |
| *SmbHLH114* | ARHRRERISERIRILQRLVPGGTKMDTASMLDEAIHYVKFL |
| *SmbHLH115* | QRMTHIAVERNRRRQMNDYLAVLRSLMPPSYAQRGDQASIVGGAINFVKELE |
| *SmbHLH116* | SHISVERNRRKQMNEHISVLRSLMPCFYVKRGDQASIIGGVVNYIKELQ |
| *SmbHLH117* | AAKNRRERISERLKILQELVPNGSKVDLVTMLEKAISYVKFLQ |
| *SmbHLH118* | MAERRRRKRLNDRLSMLRSVVPRiSKMDRASILGDTIDYMREL |
| *SmbHLH119* | KSHSEAERWLRERiINNHLAKLRSLLPNTTKTDKASLLAEVIQHVKEL |
| *SmbHLH120* | SHSLAERVRRERISERMKLLQTLVPGCDKVTGKALMLDEIINYVQSLQ |

| | |
|---|---|
| *SmbHLH121* | FQRLREIIPNSEQKRDKASFLLEVIEYIQFLQ |
| *SmbHLH122* | VHNLSERRRRDRINEKMRALQELIPRCNKSDKASMLDEAIEYLKSLQ |
| *SmbHLH123* | RSHSEAERRRRERINAHLESLRGLVPNNEKMDKATLLAEVISQIKQLR |
| *SmbHLH124* | DQINELVSKLQQLLPEMHNRRSDKKSATKVLQETCNYIRSL |
| *SmbHLH125* | DQIADLVSKLQQLIPEiRSRRSDKASASKVLQETCNYIRNL |
| *SmbHLH126* | SIAERERRTRISGKLRKLQDLVPNMDKQTSYADMLDLAVQHIKTLQ |
| *SmbHLH127* | HSHYKRRRDNKINKRLRVLRNLIPNCTQMDKESVLDEAIVYLKCLQ |

**Table S9.** Primers used for quantative real-time reversr transcription PCR

| Gene name | Primer name | Sequence (5' to 3') |
|---|---|---|
| SmDXS2 | DXS2-F | CAGCGCCCATATTTCTCATTTCTTAT |
| | DXS2-R | GAAGACTGTCTTTTCCCCCACCAAT |
| SmActin | Actin-F | AGGAACCACCGATCCAGACA |
| | Actin-R | GGTGCCCTGAGGTCCTGTT |
| SmbHLH37 | bHLH37-F | AAGGCTTGAAGATGCTGTTCTA |
| | bHLH37-R | ACCGCTCTCTGTTTGAATGG |
| SmbHLH51 | bHLH51-F | TCGCCATTCTCGGATCACTA |
| | bHLH51-R | GACTCTCGTTCTGTTGCTTCT |
| SmbHLH53 | bHLH53-F | TGAGCGATGCGTTCAAGTC |
| | bHLH53-R | AACGACGACCTAACAACCTTC |
| SmbHLH60 | bHLH60-F | ATCTCCAGAGACTTCCTTCACT |
| | bHLH60-R | CTGTGCTGATGCTGATTGTTG |
| SmbHLH74 | bHLH74-F | ACTGCTGGTGCGATGTAATC |
| | bHLH74-R | AGCCTCTTTCCTCAAGTGTTG |
| SmbHLH92 | bHLH92-F | GCTTGCCGATTGTCCCATT |
| | bHLH92-R | GGAAGAAGAGACCTGAGTGAAG |
| SmbHLH103 | bHLH103-F | CAGAAACAAGTTGAACGGAGAC |
| | bHLH103-R | CAGCAAGGACTCGTGATAGC |
